# Re-design and evaluation of diclofenac-based carborane-substituted prodrugs and their anti-cancer potential

**DOI:** 10.1038/s41598-024-81414-x

**Published:** 2024-12-16

**Authors:** Christoph Selg, Vuk Gordić, Tamara Krajnović, Antonio Buzharevski, Markus Laube, Aleksandr Kazimir, Peter Lönnecke, Mara Wolniewicz, Menyhárt B. Sárosi, Jonas Schädlich, Jens Pietzsch, Sanja Mijatović, Danijela Maksimović-Ivanić, Evamarie Hey-Hawkins

**Affiliations:** 1 Department of Chemistry and Mineralogy, Institute of Bioanalytical Chemistry, Leipzig University, Deutscher Platz 5, 04103 Leipzig, Germany; 2 Department of Immunology, Institute for Biological Research “Siniša Stanković” - National Institute of the Republic of Serbia, University of Belgrade, Bulevar despota Stefana 142, Belgrade, 11108 Serbia; 3 Department of Chemistry and Mineralogy, Institute of Inorganic Chemistry, Leipzig University, Johannisallee 29, 04103 Leipzig, Germany; 4 Department of Radiopharmaceutical and Chemical Biology, Institute of Radiopharmaceutical Cancer Research, Helmholtz-Zentrum Dresden-Rossendorf, Bautzner Landstraße 400, 01328 Dresden, Germany; 5 Institute for Drug Discovery, Leipzig University, Brüderstraße 34, 04103 Leipzig, Germany; 6 Department of Chemistry and Mineralogy, Institute of Organic Chemistry, Leipzig University, Johannisallee 29, 04103 Leipzig, Germany; 7 Faculty of Chemistry and Food Chemistry, School of Science, Technische Universität Dresden, Mommsenstraße 4, 01069 Dresden, Germany

**Keywords:** Cyclooxygenase inhibitors, Carboranes, Non-steroidal anti-inflammatory drug, Cancer, Drug repurposing, Drug discovery and development, Drug development

## Abstract

In this study, we investigated a novel anti-cancer drug design approach by revisiting diclofenac-based carborane-substituted prodrugs. The redesigned compounds combine the robust carborane scaffold with the oxindole framework, resulting in four carborane-derivatized oxindoles and a unique zwitterionic amidine featuring a *nido*-cluster. We tested the anti-cancer potential of these prodrugs against murine colon adenocarcinoma (MC38), human colorectal carcinoma (HCT116), and human colorectal adenocarcinoma (HT29). The tests showed that diclofenac and the carborane-substituted oxindoles exhibited no cytotoxicity, the dichlorophenyl-substituted oxindole had moderate anti-cancer activity, while with the amidine this effect was strongly potentiated with activity mapping within low micromolar range. Compound **3** abolished the viability of selected colon cancer cell line MC38 preferentially through strong inhibition of cell division and moderate apoptosis accompanied by ROS/RNS depletion. Our findings suggest that carborane-based prodrugs could be a promising direction for new anti-cancer therapies. Inhibition assays for COX-1 and COX-2 revealed that while diclofenac had strong COX inhibition, the re-engineered carborane compounds demonstrated a varied range of anti-cancer effects, probably owing to both, COX inhibition and COX-independent pathways.

## Introduction

**Fig. 1:**
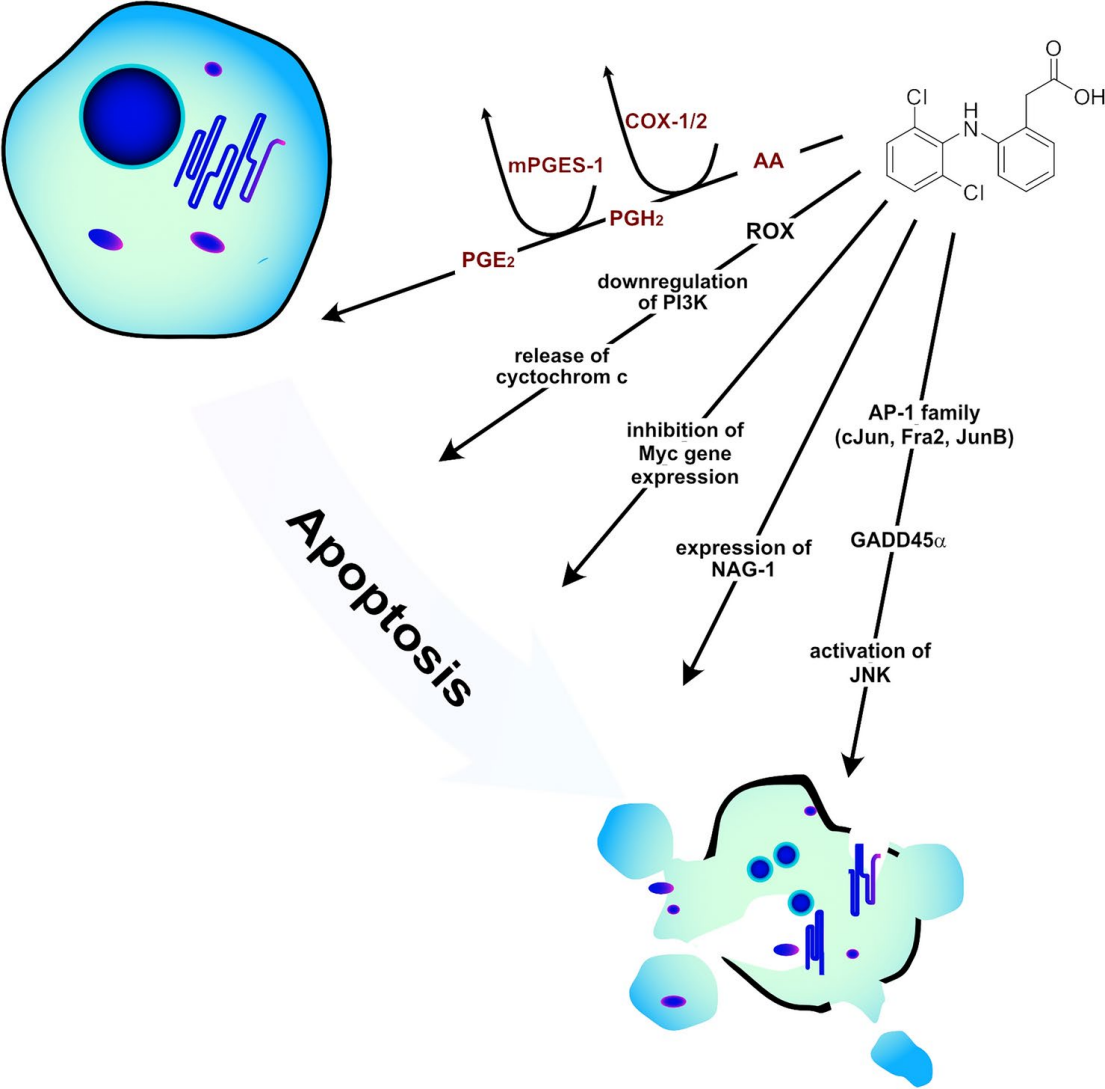
Overview of COX/$\hbox {PGE}_{2}$-dependent (red) and -independent (black) pathways for induction of apoptosis by diclofenac.

Drug repurposing, often termed drug repositioning, involves re-evaluating existing drugs for new therapeutic uses beyond their original medical indications^1^. This strategy is promising as it can accelerate the deployment of therapies drastically by bypassing some of the lengthy phases of drug development. Notable examples for this effect are the anti-malaria drugs chloroquine and hydroxychloroquine, and the anti-ebola drug remdesivir that have been evaluated and proven effective for the treatment of severe coronavirus disease 2019 (COVID-19) despite the raging pandemic^2^. The medicinal field that also particularly benefits from accelerated drug design strategies is oncology. Notable examples of repurposing of drugs in oncology (ReDO) include metformin, originally developed as an antidiabetic and bexarotene, which was initially approved for cutaneous T-cell lymphoma and has now been investigated for its effectiveness against lung cancer^3,4^. Another promising example is diclofenac, commonly known as a nonsteroidal anti-inflammatory drug (NSAID) for pain management^5–7^. Its potential as an anti-cancer agent has been explored across various solid tumors such as colon adenocarcinoma^8,9^, neuroblastoma^10^, and ovarian cancer^11^, as well as in hematological malignancies including lymphomas^5,7,12^. This divergence in the application highlights the drug’s adaptability and efficacy across a spectrum of distinct cancer types, leveraging both common and unique mechanisms of action inherent to solid and liquid tumors^7^. Clinical trials, extensive research and literature support diclofenac’s anti-cancer capabilities, attributed to its multiple pathways that can be categorized in those dependent on cyclooxygenase enzymes (COX) and COX-independent (Fig. 1)^7^.

Like most NSAID, diclofenac binds to COX, thereby blocking the production of prostaglandins. In this context, the function of prostaglandins, particularly $\hbox {PGE}_{2}$, is especially critical^13,14^: $\hbox {PGE}_{2}$ is produced when arachidonic acid is converted to prostaglandin $\hbox {H}_{2}$ ($\hbox {PGH}_{2}$) by the COX isoforms COX-1 and COX-2, and then is further processed by microsomal prostaglandin E synthase-1 (mPGES-1)^15^. As regulation of mPGES-1 and COX-2 are impaired in various types of cancer, high levels of mPGES-1 and $\hbox {PGE}_{2}$ are present and are linked to the chronic inflammation that contributes to a tumor-promoting microenvironment rendering COX, and especially COX-2 a strategic target for anti-cancer therapy^16–21^. Inhibiting COX and thus the production of prostaglandins was shown to cause apoptosis, cell cycle arrest, inhibition of metastasis and angiogenesis, as well as an increase in radio- and chemotherapeutic treatment sensitivity^21–26^. Additionally, diclofenac’s anti-cancer actions could also be attributed to several COX/PGE_2_-independent pathways^27^: These include down-regulation of Myc gene expression and glycolysis and inhibition of cellular lactate efflux decreasing cell proliferation^28–31^. Furthermore, pro-apoptotic activity was shown for diclofenac *via* inhibition of $\beta$-catenin through high-level expression of peroxisome proliferator-activated receptor-$\gamma$ (PPAR-$\gamma$)^32^. In other studies, diclofenac induced apoptosis caused by increase in intracellular reactive oxygen species (ROS), with subsequent inhibition of Akt phosphorylation via a PI3 kinase (PI3K) pathway^33^. In leukemic HL-60 and THP-1 cell lines, apoptosis was triggered by GADD45$\alpha$ expression and subsequent JNK activation, which in turn was caused by an upstream activation of AP-1 family transcription factors, including c-Jun, JunB, and Fra-2^34^. Finally, diclofenac’s pro-apoptotic action was associated with increased expression of NSAID activated gene 1 (NAG-1), which is linked to apoptosis in various cancers^35^.

**Fig. 2:**
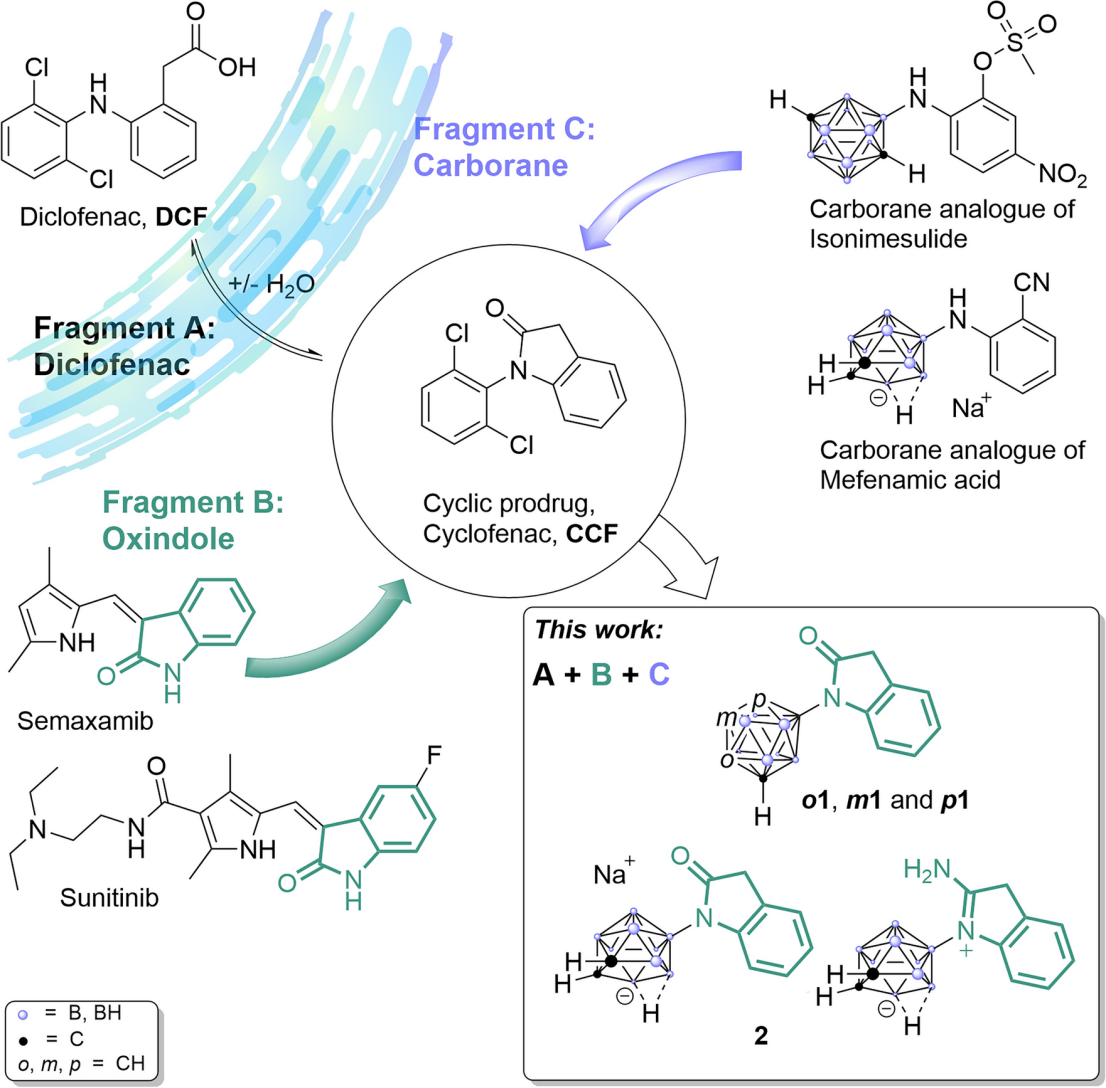
Fragment-based design concept for the new carborane-based anti-cancer agents: a combination of insights from diclofenac (fragment A), oxindoles (fragment B) and carborane-substituted NSAID (fragment C).

Despite the promising evidence of anti-cancer drug potential of diclofenac, the gastroulcerogenic effects associated with prolonged NSAID use necessitate co-administration with e.g. proton pump inhibitors during chronic treatments, such as chemotherapy. The ulceration is most likely caused by inhibition of prostaglandin synthesis in the stomach, which is crucial for stimulating mucus production, and a local effect caused by the direct contact of the acidic drugs with the gastric mucosa^36,37^. To mitigate these adverse effects, several prodrugs and derivatives of diclofenac, including esters^37–39^, alcohols^20^, aldehydes, alkanes^40^, oxadiazoles^41,42^, and amides have been developed^40^. Notably, the intramolecular amide or lactam of diclofenac, *N*-dichlorophenyl-oxindole (cyclofenac, **CCF**, Fig. 2) was shown to match the pharmacokinetics and anti-inflammatory efficacy of diclofenac but without causing gastrotoxicity^40,43,44^.

Although initial in vitro tests on MCF7, MiaPaCa-2, KB, HuTu80, L132, B16F10 and Molt4 cancer cells showed no effect^45^, in 2021 moderate efficacy against B16-F10, Hep-G2 and HT29 cell lines was reported (IC_50_ values 40.77 ± 1.33, 24.53 ± 1.11 and 40.77 ±1.33 μM respectively)^20^. In in vivo studies, hydrolysis of the lactam to form the open-chain diclofenac could be demonstrated^43,44^.

Furthermore, also various derivatives of the closed-ring oxindole scaffold, such as sunitinib and semaxamib (Fig. 2), have proven to be potent anti-cancer agents^46–49^. These findings led us to attempt the assembly of a new anti-cancer compound adaption of the concept of fragment-based drug discovery^50^. This method uses fragments of successful drugs as building blocks to create new therapeutic agents, leveraging high-resolution structural data to identify small chemical fragments that effectively bind to the target protein^50^. These fragments are then optimized and combined to enhance their efficacy and specificity which enables high hit rates, more manageable molecular sizes, and the ability to fine-tune drug properties for enhanced performance. One prominent example of new fragments that showed improved anti-cancer activity in combination with NSAID scaffolds is the carborane fragment^51–57^. Carboranes are polyhedral clusters that consist of CH and BH vertices, with *closo*-dicarbadodecaborane $\hbox {C}_{2}\hbox {B}_{10}\hbox {H}_{12}$, an icosahedral structure, being the most notable and thoroughly examined variant. Within this icosahedron, the positioning of the two carbon atoms can vary (in analogy to benzene substitution), leading to three isomers with the carbon atoms separated by one (*ortho*), two (*meta*), or three (*para*) bonds. These vertices are interconnected by a durable network of multi-electron-multi-center bonds, which create a three-dimensional $\sigma$-aromatic molecule of extraordinary thermodynamic and chemical stability^58–63^. Their hydrophobic nature as well as the option of Lewis-base induced deboronation creating an anionic nido-cluster $(\hbox {C}_{2}\hbox {B}_{9}\hbox {H}_{12})^{-}$ configuration have paved the way for their incorporation into numerous new therapeutic agents. This (bio)isosteric replacement strategy has been widely applied in medicinal chemistry, materials science and catalysis and often yielded increased selectivity and potency, as well as decreased toxicity^64–73^. While carborane-containing drugs show promise as therapeutic agents, the field remains in its early stages, and comprehensive studies on their in vivo metabolism and toxicity are still scarce in literature. Most foundational in vivo research comes from the field of boron neutron capture therapy (BNCT), in boron cluster-based nanomedicine and theoretical studies involving co-crystal structures and molecular docking^74–77^. More extensive research exists on single boron compounds in medicinal chemistry, though cluster chemistry presents distinct challenges; nevertheless, these studies offer a valuable starting point for future in vivo investigations of boron clusters^78–81^. Integrating insights from the anti-cancer actions of diclofenac with those of established oxindole-containing drugs, in the present publication, we thus set out to harness the latest findings on carborane-based NSAID, such as analogs of mefenamic acid^56^, indomethacin^52,53,55^, nimesulide,^82^ rofecoxib,^83^ and celecoxib (see Fig. 2)^51^. Despite being initially designed to enhance isoform selectivity for the bigger cavity of COX-2 *via* size exclusion (van-der-waals-volume of carboranes: 141–148 Å^3^ versus 102 Å^3^ for a rotating phenyl ring), these compounds typically displayed only weak COX affinity and minimal COX-2 selectivity (except for *nido*-indoborin)^84^. However, these carborane NSAID often exhibit significant COX-independent anti-cancer properties.

By merging these concepts, we aimed to develop a small series of novel anti-cancer drugs based on the diclofenac scaffold. This approach embodies the principles of ReDO, integrating knowledge of oxindole-based anti-cancer drugs and oxindole derivatives as prodrugs of diclofenac with innovative applications of carborane-based anti-cancer agents and their phenyl analogs.

## Results and discussion

### Synthesis and COX-affinity

**Fig. 3:**
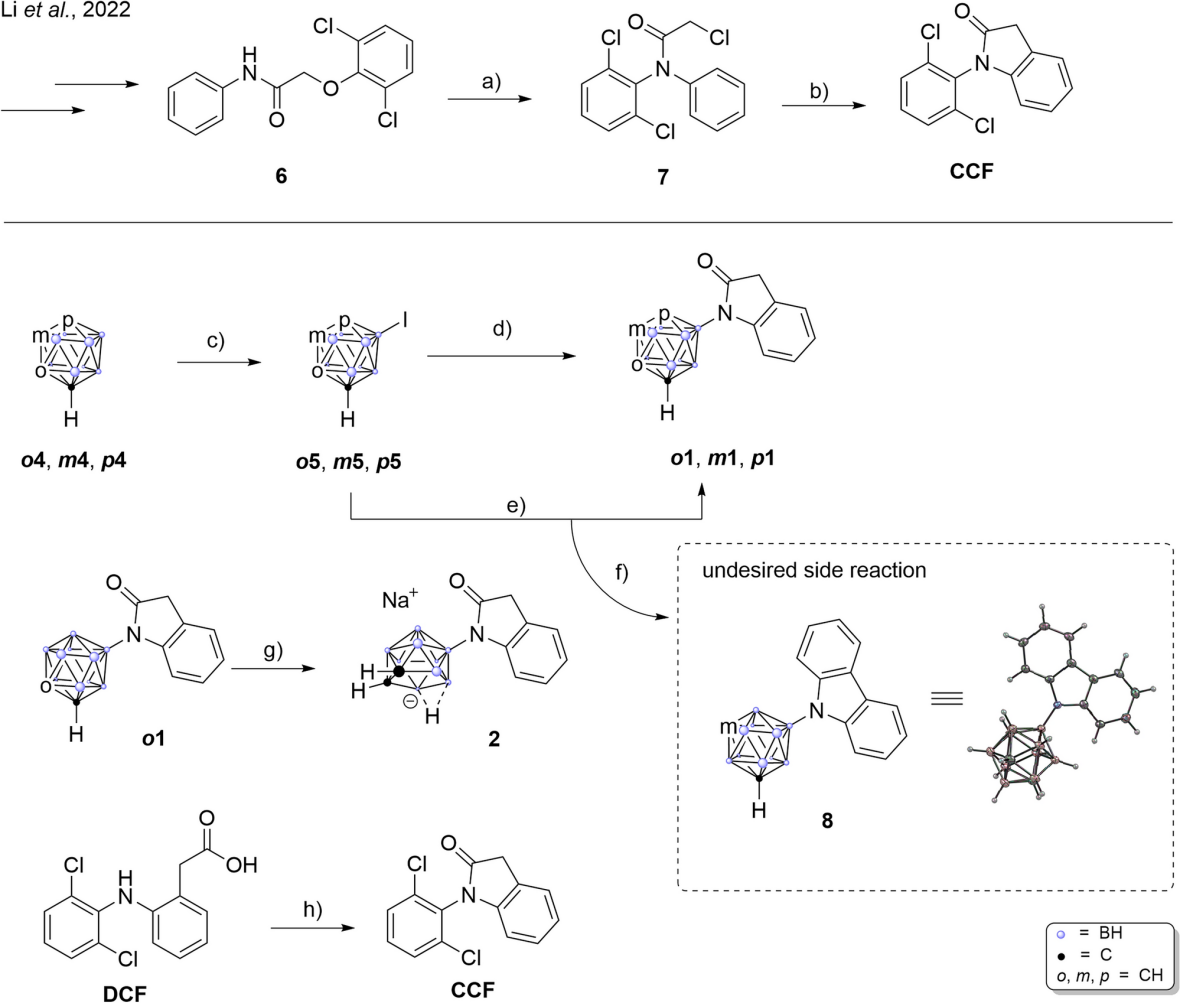
Synthesis of the diclofenac inspired carborane compounds ***o*1**, ***m*1**, ***p*1** and **2**. Reaction conditions: (**a**) i. NaOH, toluene, $115\,^{\circ }\hbox {C}$, 3h, 89%; ii. chloroacetyl chloride, neat, 3 h, $125\,^{\circ }\hbox {C}$, 92%^38^; (**c**) $\hbox {I}_{2}$, $\hbox {H}_{2}\hbox {SO}_{4}$, HNO_3_, AcOH, 60-$80\,^{\circ }\hbox {C}$, 80-96%; (**d**) oxindole, $\hbox {KO}^{\textit{t}}\hbox {Bu}$, SPhosPdG4 (methanesulfonato(2-dicyclohexylphosphino-2’,6’-dimethoxy-1,1’-biphenyl)(2’-methylamino-1,1’-biphenyl-2-yl)palladium(II)), 1,4-dioxane, $90\,^{\circ }\hbox {C}$, 58%; (**f**) oxindole, $\hbox {KO}^{\textit{t}} \hbox {Bu}$, SPhosPdG3 ((2-dicyclohexylphosphino-2’,6’-dimethoxybiphenyl) [2-(2’-amino-1,1’-biphenyl)]palladium(II) methanesulfonate), 1,4-dioxane, $90\,^{\circ }\hbox {C}$, 10%; (**g**) NaF, ethanol/$\hbox {H}_{2} \hbox {O}$ 3:1, $70\,^{\circ }\hbox {C}$, quant.; (**h**) $\hbox {SOCl}_{2}$, $\hbox {CH}_{2}\hbox {Cl}_{2}$, cat. DMF, $40\,^{\circ }\hbox {C}$, quant.

For our series of carborane-substituted oxindoles, we first chose to replace the eponymous dichlorophenyl-residue of diclofenac with one of the three carborane isomers. In the recent literature procedures for the synthesis of diclofenac, *N*-dichlorophenyl aniline **7** is synthesized *via* Smiles-rearrangement and *N*-acetylation with chloroacetyl chloride to form an amide that in turn is used in a Friedel-Crafts alkylation affording the *N*-dichlorophenyl-oxindole^39^. Instead of the rearrangement strategy, we chose to use a palladium catalyzed Buchwald-Hartwig type coupling reaction between oxindole and 9-iodo-*meta*-carborane (Fig. 3. ***m*5**) as previously described^85,86^. A first attempt using NaH as base and the catalyst system of 2.4 mol% $\hbox {Pd}_{2}\hbox {(dba)}_{3}$ ((1*E*,4*E*)-1,5-diphenylpenta-1,4-dien-3-one) and 2.5 mol% BINAP (*rac*-(±)-2,2’-Bis(diphenylphosphino)-1,1’-binaphthyl) in 1,4-dioxane afforded carborane analog ***m*1** in 30% yield.

**Fig. 4:**
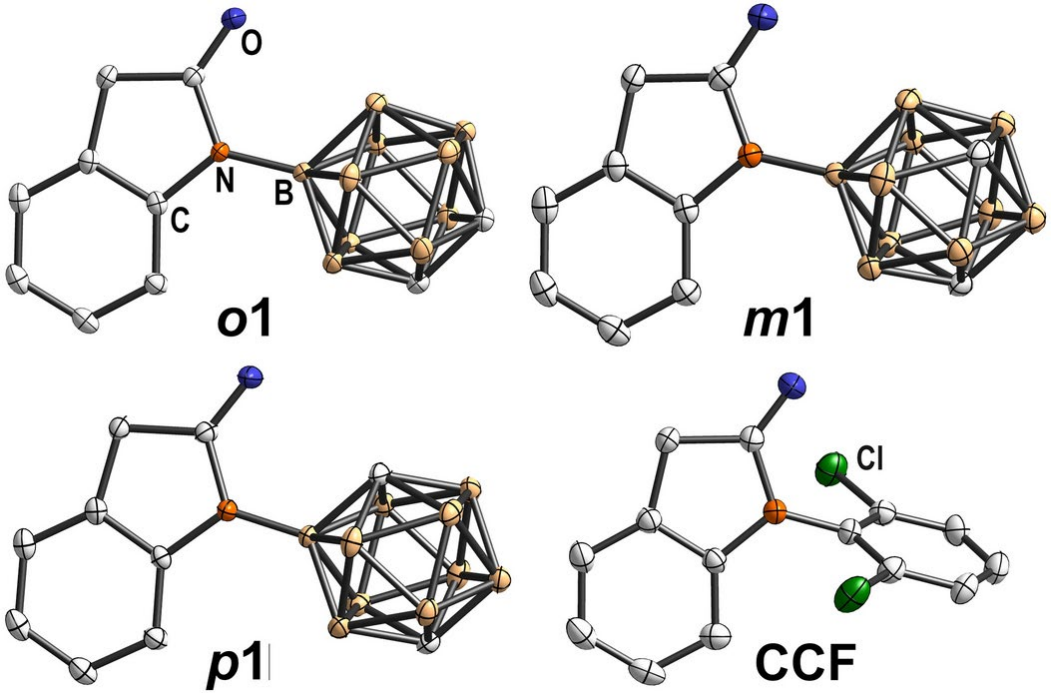
Molecular structures of the oxindoles ***o*1**, ***m*1**, ***p*1** and **CCF** with displacement thermal ellipsoids drawn at 50% probability level. The structure of **CCF** was taken from the CDCC reference 722968^91^. Detailed crystallographic parameters can be found in the Supporting Information.

With this proof of concept, we optimized the reaction conditions employing several palladium catalysts^87,88^. When the third generation catalyst SPhosPdG3 was used, under our reaction conditions the undesired carbazole-forming side reaction that led to the development of the fourth generation catalyst systems (SPhosPdG4) was so pronounced, that 9-*N*-carbazyl-1,7-dicarba-*closo*-carborane **8** was formed quantitatively (with respect to the catalyst loading)^89,90^. We were, however, able to synthesize the isomeric oxindoles ***o*1**, ***m*1**, and ***p*1** in 58%, 67%, and 78% yield respectively, using *N*-methylated fourth generation catalyst SPhosPdG4. Finally, the sodium salt of the *nido*-isomer **2** was obtained by NaF mediated deboronation of ***o*1** in ethanol and water in 76% yield (Fig. 3, middle and Fig. 6, left)^56^.

(Note, that deboronation of an unsymmetrically substituted *closo*-cluster gives a pair of planar chiral enantiomers in the ratio 1:1). All three carborane derivatives ***o*1**, ***m*1**, and ***p*1** readily crystallized and were suitable for single crystals X-ray analysis (Fig. 4).

For proper comparison, we used commercially available diclofenac (**DCF**) from which lactam **CCF** was synthesized in quantitative yield using thionyl chloride and catalytic *N*,*N*-dimethyl formamide (Fig. 3). To estimate the functionality as a prodrug and the stability of the oxindoles, we investigated the behavior in solution at several pH-levels, with different acids, bases, temperatures and solvents (see Supporting Information). As the *para*-carborane usually shows the highest stability towards deboronation, ***p*1** was used as model compound. The lactam proved extraordinarily stable towards hydrolysis and the open chain derivative was only observed under harsh conditions, but at both ends of the pH scale. The alkaline hydrolysis was successful with CsOH in a degassed 1:3 water-ethanol mixture at $80\,^{\circ }\hbox {C}$, while acidic hydrolysis happened at $100\,^{\circ }\hbox {C}$ in 40% aqueous sulfuric acid with 2% acetic acid. Upon attempted isolation however, the lactam was re-formed within minutes as observed by $^{1}$H and ^11^B{$^{1}$H} NMR spectroscopy (see Supporting Information). High stability towards hydrolysis was also observed by Chung and co-workers for the dichlorophenyl analog **CCF** in buffer solutions at pH 1.2 and pH 7.4 and in human serum and the open chain diclofenac was only detected in vivo^36,43^. However, a condensation reaction back to the oxindole upon isolation was not described for diclofenac and seems to be unique to the carborane analogs and a few differently substituted examples in the literature^92,93^. The reclosing to the oxindole ring might be attributed to an increased nucleophilicity of the amino nitrogen atom stemming from its association to the carborane cluster *via* a cluster boron atom. This is supported by density functional theory (DFT) calculations (see Supporting Information). With the five oxindoles **CCF**, ***o*1**, ***m*1**, ***p*1** and **2** at hand, we performed a preliminary COX-2 assay using the COX Fluorescent Inhibitor Screening Assay Kit (Cayman Chemical Company) and **DCF** as a reference at an initial concentration of 100 μM (the results are summarized in Table 1).

**Table 1:** COX Inhibition of compounds **CCF**, ***o*1**, ***m*1**, ***p*1**, **2**, and **3** with **DCF**, COX-2 inhibitor Celecoxib and COX-1 inhibitor **SC-560** as the reference as determined in a COX fluorescent inhibitor screening assay.

#	IC_50_ [μM] (95% CI^[a]^)		SI_1/2_ [b]	%Inh. @ 100 μM (n = 2)
	COX-1	COX-2		COX-2
DCF	$3.8\pm 0.0333^{\text{[c]}}$	$0.84\pm 0.0333^{\text{[c]}}$	4.5	102
Celecoxib	–^[d]^	$0.056\pm 0.010^{\text{[e]}}$	–	–
SC-560	$0.012\pm 0.002^{\text{[e]}}$	–^[d]^	–	–
CCF	–^[f]^	–	–	5
*o*1	–	–	–	14
*m*1	–	–	–	19
*p*1	–	–	–	$\hbox {n.i.}^{\text{[h]}}$
2	$>100^{\text{[i]}}$	58.5 (45.0–79.9)	1.7	56
3	15.3 (13.7–17.2)	4.58 (3.49–6.00)	3.3	105

[a] CI = Confidence interval. [b]Selectivity index, SI_1/2_ = IC_50_(COX-1)/IC_50_(COX-2). [c]IC_50_ values for diclofenac at ovine COX-1 and human recombinant COX 2 were taken from the literature^94^. [d]Not determined. [e]Data are presented as mean±SD of three independent experiments. [f]No IC_50_ value definable. [g]263 (179-infinity), fit ambiguous. [h] No inhibition (%inhibition below 5%). [i]337 (CI not definable). For additional information, see Supporting Information.

**Fig. 5:**
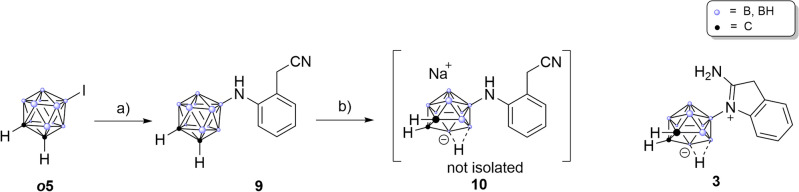
Synthesis of the diclofenac inspired nitrile compound **9** and subsequent decomposition to amidine **3**. Reaction conditions: (**a**) 2-(2-Aminophenyl)acetonitrile, $\hbox {KO}^{\textit{t}} \hbox {Bu}$, SPhosPdG4, 1,4-dioxane, $90\,^{\circ }\hbox {C}$, 58%; (**b**) degassed ethanol/water 3:2, NaF, $90\,^{\circ }\hbox {C}$, 18h, quant.

The only compound that showed considerable enzyme inhibition (56%) was *nido*-derivative **2**. This observation correlates with previous findings, where also the *nido*-derivative showed the most promising results in COX assays^52,56^. We thus decided to expand the series with one more *nido*-compound before proceeding with the advanced biological evaluation. Useini et al. had found in their study of the carborane derivatives of mefenamic acid, that COX-affinity was highest for the *nido*-derivative featuring a nitrile group instead of the carboxylic acid^56,95,96^. Following the synthetic protocol, we set out to synthesize nitrile compound **9** from 9-iodo-*ortho*-carborane ***o*5** and 2-(2-aminophenyl)acetonitrile employing the previously used *N*-methylated SPhosPdG4 which cleanly afforded *closo*-derivative **9** in 58% yield. The compound was isolated and fully characterized, but proved to be unstable in solution (DMSO-$d_{6}$); after approximately five hours decomposition to the corresponding amidine species **3** was observed (Fig. 5) by ^11^B{$^{1}$H} and $^{1}$H NMR spectroscopy (see Supporting Information).

**Fig. 6:**
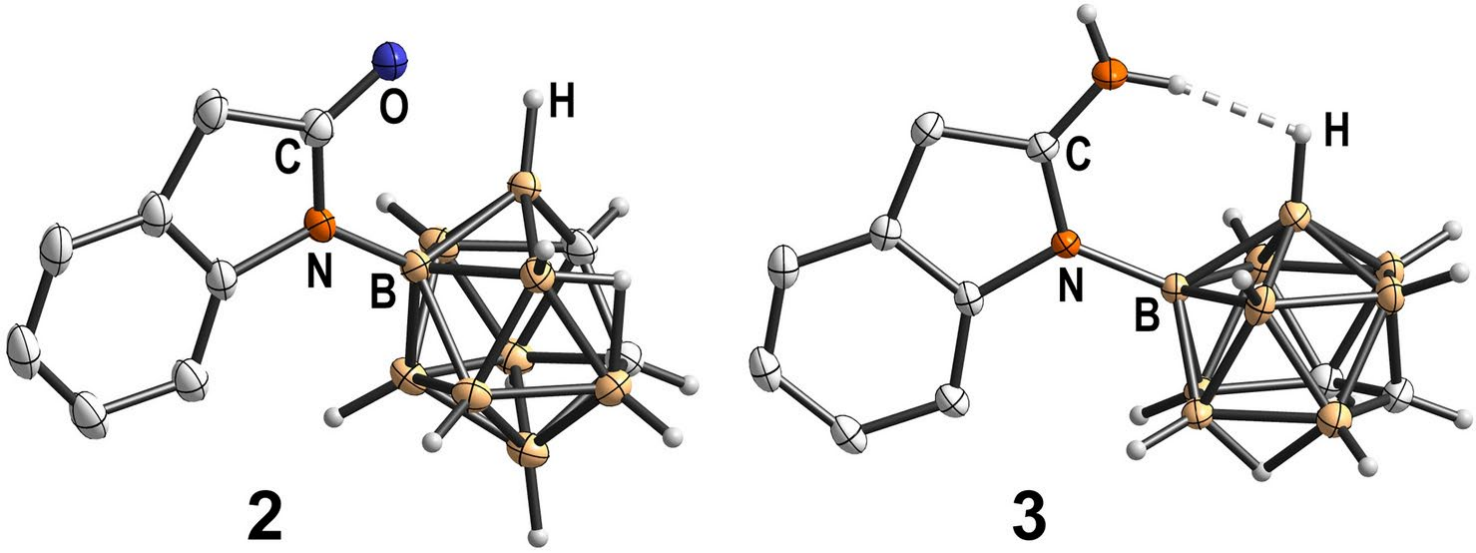
Molecular structures of *nido*-carboranyl oxindole **2** and amidine **3** with displacement thermal ellipsoids are drawn at 50% probability level. Only the $\textit{S}_{\textrm{p}}$-enantiomers are shown. The bond lengths inside the seven-membered ring featuring an intramolecular dihydrogen bond (1.738 Å) is indicated. Detailed crystallographic parameters can be found in the Supporting Information.

This transformation most likely proceeded through deboronation affording an extremely electron-rich secondary amine **10** that in turn attacks the nitrile carbon atom to form the amidine. When classical deboronation conditions (Fig. 5b) were applied, nitrile **9** reacted smoothly to amidine **3** in quantitative yield. In contrast to **9**, the amidine species is stable in air and moisture and can be stored infinitely without any signs of decomposition. In the molecular structure of **3** (Fig. 6), a nearly linear (N-H^..^H-angle: $167.76^{\circ }$) intramolecular dihydrogen bond (1.738 Å) between the cluster B-H and the *exo*-N-H moiety of the amidine is observed forming a distorted seven-membered ring and rendering the backbone of the molecule completely planar. This intramolecular dihydrogen bond is most likely responsible for the stabilization of this rather peculiar structure.

When tested in the COX-2 assay, compound **3** exhibited 100% inhibition at a concentration of 100 μM. The two *nido*-compounds **2** and **3** were subsequently tested for their isoform selectivity. For both compounds, the affinity to COX-2 was higher than for COX-1 (see Table 1). IC_50_ values for lactam **2** were > 100 μM (COX-1) and 58.5 μM (COX-2), and for the amidine **3**, 15.3 μM (COX-1) and 4.58 μM (COX-2). In comparison, the IC_50_ values of the native open-ring **DCF** are smaller by a factor of 3.9 for COX-1 and factor 5.5 for COX-2 with a slightly higher selectivity index SI_1/2_ of 4.5. In accordance with previously reported data, the COX-inactive lactam ring of **CCF** is not hydrolysed under the assay conditions^36,43^. The *nido*-compounds **2** and **3** are thus moderately active COX-inhibitors with a slight preference for COX isoform **2**, while closed-ring dichlorophenyl analog **CCF** shows no inhibition at all under in vitro conditions.

### Cytotoxicity

The anti-cancer properties of the five carborane analogs ***o*1**, ***m*1**, ***p*1**, **2**, and **3**, as well as their open- and closed-ring reference compounds **CCF** and **DCF**, were tested against three cancer cell lines: murine colon adenocarcinoma (MC38), human colorectal carcinoma (HCT116) and human colorectal adenocarcinoma (HT29). In a preliminary test, the stability of all compounds dissolved in the cell medium (*c* = 100 μM, T = $40\,^{\circ }\hbox {C}$) for 72 h was validated by HPLC (see Supporting Information). Cell lines were incubated with the respective compounds in a concentration range of 1.56–100 μM for 72 h, and cell viability was measured using 3-(4,5-dimethylthiazol-2-yl)-2,5-diphenyltetrazolium bromide (MTT) and crystal violet (CV) assays. The calculated IC_50_ values were incorporated in Table 2, while the viability curves comparing **DCF**, **CCF**, and compound **3** as the only active derivative are presented in the Supporting Information. As MTT tests gave inconsistent results, possibly due to interference of the compound with mitochondrial respiration, for the further evaluation only the CV results are considered^97,98^.

**Table 2:** IC_50_ values [μM] of **DCF**, **CCF**, ***o*1**, ***m*1**, ***p*** **1**, **2**, and **3** on cancer cell lines MC38, HCT116, and HT29.

#	IC_50_ [μM] (n = 3)					
	MC38		HCT116		HT29	
	MTT	CV	MTT	CV	MTT	CV
DCF	> 100	> 100	> 100	> 100	> 100	> 100
CCF	32.6 ± 2.7	75.1 ± 3.8	46.7 ± 2.4	73.5 ± 3.9	77.7 ± 5.5	74.4 ± 4.8
*o*1	> 100	> 100	> 100	> 100	> 100	> 100
*m*1	> 100	> 100	> 100	> 100	> 100	> 100
*p*1	> 100	> 100	> 100	> 100	> 100	> 100
2	> 100	> 100	> 100	> 100	> 100	> 100
3	7.9 ± 0.2	9.5 ± 0.4	6.9 ± 1.5	11.5 ± 1.0	17.0 ± 1.6	17.6 ± 0.3

Data are presented as mean±SD of three independent experiments obtained by MTT and CV tests.

Diclofenac and all four carborane-substituted oxindoles ***o*1**, ***m*1**, ***p*1**, and **2** showed no effect on cell viability at concentrations up to 100 μM. In contrast, the phenyl analog **CCF** demonstrated a moderate effect, with IC_50_ values around 74 μM. Amidine **3**, however exhibited significant cytotoxicity in the single-digit micromolar range (9.5 μM) and consequently was chosen for further investigation. On the other hand, IC_50_ values obtained on peritoneal exudate cells (PECs) isolated from healthy mice and on the human fetal lung fibroblast cell line MRC-5, were 2-3 times higher, indicating a moderate selectivity towards malignant cells (see Supporting Information).

### Flow cytometry and mechanism of cell death

To specify the main mechanisms responsible for the strong cytotoxicity observed in colon cancer cell cultures, MC38 cells were exposed to compound **3** for 72 h, and the presence of programmed cell death type I and II was assessed using flow cytometry and fluorescence microscopy.

**Fig. 7:**
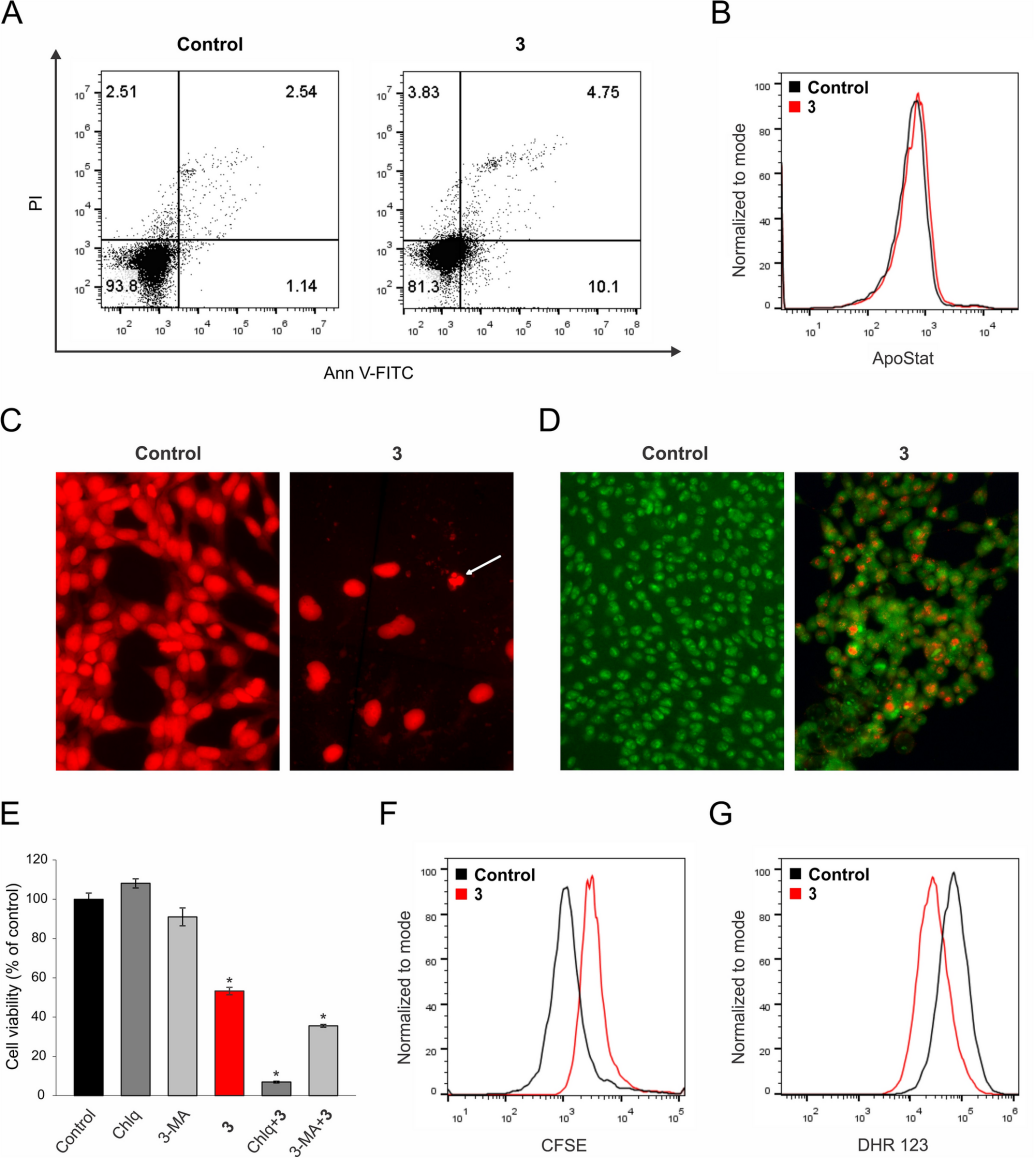
The mechanism of action of compound **3** on the MC38 cell line was evaluated using flow cytometry, fluorescence microscopy (400-fold magnification), and viability assay. Cells were exposed to the IC_50_ concentration of compound **3** for 72 h followed by the corresponding staining, AnnV-FITC/PI (**A**), ApoStat (**B**), PI (white arrow indicate apoptotic nuclei) (**C**), AO (**D**), CFSE (**F**) and DHR 123 (**G**). Representative charts and micrographs from three independent experiments are shown. Cell viability was determined upon simultaneous treatment of compound **3** with the autophagy inhibitors chloroquine (Chlq) and 3-methyladenine (3-MA), using the CV assay (**E**). *p < 0.05 compared to control cultures.

Annexin V fluorescein isothiocyanate conjugate and propidium iodide (AnnV-FITC/PI) double staining revealed a moderate apoptotic cell accumulation after the treatment with compound **3** within the indicated time interval. At the end point of the incubation period, the apoptotic cells were mostly in the early phase of dying process with inverted phosphatidylserines, but still preserved their membrane permeability ($\hbox {Ann}^{+}$/PI^−^) (Fig. 7A). There was no significant change in total caspase activity between cultures exposed to the IC_50_ concentration of experimental drug **3** and controls, suggesting that the apoptotic process was independent of caspase activation (Fig. 7B). PI staining of fixed MC38 cells through visualization of the consistency and density of genetic material confirmed the moderate involvement of apoptotic cells with high-density chromatin, shrunken nuclei, and fragmented DNA in the overall failure of cell viability (Fig. 7C). On the other hand, the presence of large nuclei with possibly duplicated but undivided genetic material, as well as cells in a state of senescence were observed. Acridine orange staining revealed an intensified presence of autophagosomes in the cytoplasm of the treated cells (Fig. 7D). Keeping in mind the dual role of the autophagic process in cell survival and death, co-treatment with specific inhibitors of autophagy, chloroquine (Chlq) and 3-methyladenine (3-MA), alternatively led to further viability decrease, clearly indicating a pro-survival role of autophagy, opposing the drug-mediated cytotoxicity (Fig. 7E). This excludes autophagy as cell death mechanism triggered by compound **3**, but underlines the opportunity to amplify novel drug action with concomitant application of chloroquine. Marginal apoptosis and the presence of cells with large nuclei evident on PI-stained chamber slides, suggested that compound **3** preferentially acts through cell division blockage which subsequently leads to apoptotic cell death starting in the last hours of the indicated incubation period. Flow cytometric assessment of cells stained with CFSE (2,5-dioxopyrrolidin-1-yl 3,6-dihydroxy-3-oxo-3*H*-spiro[[2]benzofuran-1,9’-xanthene]-6-carboxylate) confirmed this assumption, since the remarkable difference between the mean of fluorescence intensity of treated cells was significantly higher in comparison to controls, indicating the increased presence of an undivided cell fraction in the cultures exposed to compound **3** (Fig. 7F). Finally, and in concordance with efficient inhibition of COX-2 (Table 1), the production of reactive oxygen and nitrogen species (ROS/RNS) in the presence of compound **3** was strongly depleted, as determined by dihydrorhodamine (DHR 123) test (Fig. 7G). Having in mind that basic ROS production in colon cancer cells is enhanced in comparison to healthy cells and is tightly connected to the metabolic adaptation of malignant cells that serves disease progression, the scavenging capacity of compound **3** may be the core of its anti-tumor feature. Taken together, the compound **3** pivotally exhibits a profile of cytostatic drugs, basically affecting cell proliferation, thereby promoting late death signal as a consequence of the inability of cells to divide. This approach is much more favorable in advanced tumor treatment, as it has recently been documented that the presence of apoptotic cell death in the high-grade tumor microenvironment strongly stimulates tumor repopulation as a compensatory response of the tumor tissue to induction of cell death. In this context, the cytostatic potential and the delayed and mild cell death induced by compound **3** may minimize the mitogenic effects of postmortem signals delivered from apoptotic cells, resulting in tumor spreading^99^. It was recently reported that carborane-containing derivatives of NSAID such as mefenamic acid and fenoprofen realized their antitumor action by concomitant inhibition of proliferation and induction of caspase independent apoptosis, even in cells which are negative to COX-2 expression, indicated COX-2 independent pathways and potential off targets involved in drug mediated cytotoxicity^56,57^. On the other hand, a carborane-based derivative of COX-2/LOX-5 dual-inhibitor tebufelon manifested remarkably stronger antitumor potential than the respective parental compound, with an exclusively cytostatic mode of action^100^. In comparison with the carborane-based NSAID but in concordance with carborane-based counterparts of the COX-2/LOX-5 dual-inhibitor tebufelon mentioned above, *nido*-carboranyl oxindole amidine **3** represents a promising candidate for further research in the field of advanced tumor therapy.

### Molecular docking

**Fig. 8:**
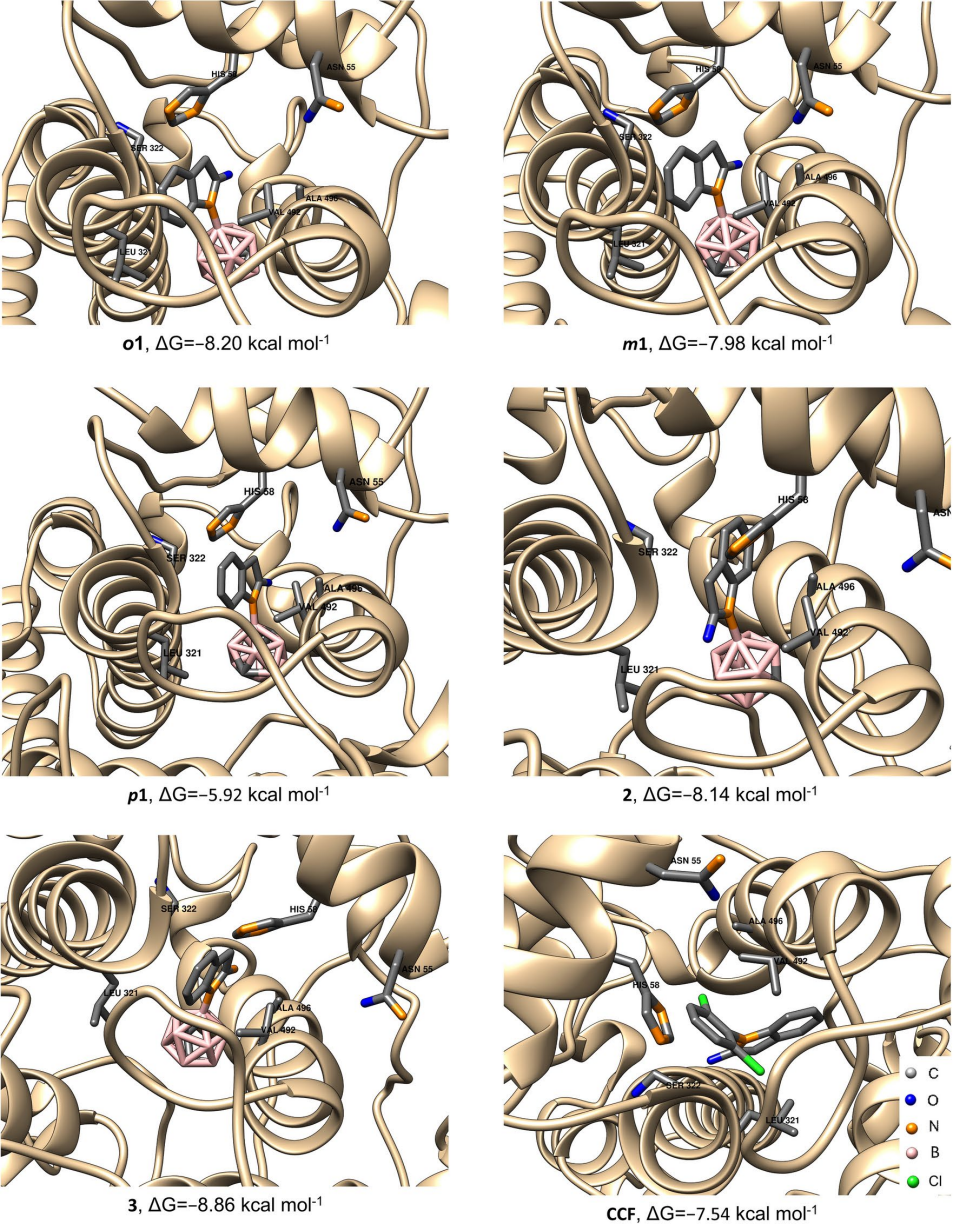
In silico investigation of the binding modes of carborane derivatives ***o*1**, ***m*1**, ***p*1**, **2** and **3** as well as phenyl analog **CCF** in COX-2. *nido*-Indoborin (PDB ID: 4Z0L)^53^ was used as the starting point for docking. The highest ranked docked positions of the compounds are shown together with the labeled amino acid residues important for the non-covalent interactions with the ligands (Leu321, Ser322, His58, Asn55, Val392, Ala496).

As the starting point for the molecular docking simulations, we selected the initial structure of COX-2 available in the protein data bank (PDB ID: 4Z0L)^53,55^. The initial ligand, *nido*-indoborin was removed from the binding pocket, subsequent docking experiments were conducted using the crystal structures of ***o*1**, ***m*1**, ***p*1**, **2**, **3** and phenyl analog **CCF** aligning them in a similar position. The energies associated with the most favorable docking conformations are summarized in Fig. 8.

The best-ranked positions of all five compounds displayed comparable non-covalent interactions with similar amino acids (Fig. 8), albeit with different binding energies ($\Delta$G). The comparative analysis revealed that compound ***p*1** displayed the lowest binding affinity (− 5.92 kcal mol^−1^), whereas amidine **3** demonstrated the highest binding potential to COX-2 (− 8.86 kcal mol^−1^). It is noteworthy that the calculated binding energy of the best docked pose of compound **CCF** was − 7.54 kcal mol^–1^. However, conformational analysis conducted on 100 conformations of this compound revealed only a single preferred conformation, demonstrating the additional stability of this pose (Fig. 8, bottom right). Collectively, the trends suggest a progressive increase in binding affinity to COX-2 across the tested carborane compounds in the order: ***p*1** < **CCF** < ***m*1** < ***o*1** $\thickapprox$ **2** < **3**. These results suggest that compound **3** binds strongly to COX-2, which is in line with the results from the COX-assays (Table 1) and possibly leading to the increased cell toxicity, likely through COX-dependent pathways (Fig. 1, Table 2).

### Conclusion

In summary, in this study, we explored a novel approach to anti-cancer drug design by re-evaluating diclofenac-based carborane-substituted prodrugs for their potential anti-cancer activity. The redesigned compounds merge the carborane scaffold, known for its remarkable stability and structural versatility, with the oxindole framework, known from established anti-cancer drugs like semaxamib. Through synthetic strategies including palladium-catalyzed Buchwald-Hartwig-type cross coupling reactions we obtained a range of four carborane derivatized oxindoles ***o*1**, ***m*1**, ***p*1**, and **2**, as well as a zwitterionic amidine species **3** featuring a *nido*-cluster. The anti-cancer properties of these prodrugs were tested on murine colon adenocarcinoma (MC38), human colorectal carcinoma (HCT116) and human colorectal adenocarcinoma (HT29) cell lines. The results demonstrated that diclofenac as well as the carborane-substituted oxindoles ***o*1**, ***m*1**, ***p*1**, and **2** showed no cytotoxicity, while the dichlorophenyl-substituted **CCF** showed moderate and the amidine **3** exhibiting strong anti-cancer potential in the single-digit micromolar range. At the core of its activity was the blockade of cell division and moderate apoptosis, compromised by the induction of autophagy. The strong ROS/RNS scavenging potential may be closely related to the abrogated viability of MC38, while cancer cell metabolism is calibrated to a higher baseline production of reactive species, which represent an important endogenous pool for sustaining proliferation. Our findings suggest that carborane-based prodrugs offer a promising route for developing new anti-cancer therapies, especially as adjuvant or combination therapies. Inhibition assays for COX-1 and COX-2 demonstrated that while the original diclofenac had strong COX-inhibition properties, the re-engineered carborane compounds presented diverse and, in some cases, enhanced anti-cancer effects, attributed to a combination of COX-inhibition and COX-independent pathways. With further development, a more robust structure-activity relationship (SAR) model will emerge, clarifying how structural modifications with carboranes influence drug properties like, e.g. cellular uptake efficiency and thus the cytotoxicity. This progress will enhance our understanding of the impact of these modifications on drug behavior.

## Experimental part

### Chemistry

Unless otherwise noted, all reactions were carried out with standard Schlenk-technique using oven dried glassware that was heated to $600\,^{\circ }\hbox {C}$ (with a heat gun) under high vacuum and refilled with dry argon three times prior to use (securated). NMR spectra were recorded on a Bruker Avance DRX 400 MHz ($^{1}$H NMR 400.16 MHz and $^{13}$C{$^{1}$H} NMR 100.59 MHz) and a Bruker Avance III 400 MHz ( $^{1}$H NMR 400.20 MHz, $^{13}$C{$^{1}$H} NMR 100.64 MHz) spectrometer. The chemical shift ($\delta$) is given in ppm. $^{1}$H NMR spectra were either referenced to Si(CH_3_)$_{4}$ as internal standard or to the solvent residual signal as internal reference for $^{1}$H and $^{13}$C{$^{1}$H} NMR spectra (CDCl_3_: $\delta$H 7.26; $\delta$C 77.0, ($\hbox {CD}_{3})_{2}$SO: $\delta$H 2.50; $\delta$C 39.5). ^11^B NMR chemical shifts were calculated according to the $\Xi$-scale^98^. Multiplicities are indicated as s (singlet), d (doublet), t (triplet), q (quartet), quint (quintet), sept (septet), m (multiplet), b (broad singlet); coupling constants (J) are in Hertz (Hz). High-resolution mass spectra were recorded using a Bruker Daltonics Impact II with electrospray-ionization and time-of-flight detection (TOF). IR spectra were recorded on a Thermo Scientific Nicolet iS5 with a diamond ATR (400–4000 cm^−1^), TLC analysis was performed on precoated silica gel 60 F_254_ slides and visualized by UV-light (254 nm) or palladium staining (5% m/v $\hbox {PdCl}_{2}$ solution in methanol). Analytical HPLC analysis was carried out using a JASCO system equipped with a JASCO PU 4180 pump, a JASCO MD 4015 photo diode array detector, a JASCO AS 4050 autosampler, and a RHEODYNE column compartment by IDEX Health & Science. The system was operated using a Daicel Chiralpak IA column (amylose based with tris(3,5-dimethylphenylcarbamate) immobilized on 5 μM silica gel, 6 mm x 25.0 mm). UV absorption was detected in the area of 209 to 338 nm with the respective solvent ratio of *n*-hexane to isopropanol at a flow rate of 1 mL min^−1^. Microwave-assisted reactions were carried out in an Initiator 8 (Biotage AB) single mode microwave at 2450 MHz controlled irradiation using standard sealed microwave glass vials (20 mL). Reaction temperatures were monitored by an IR sensor on the outside wall of the reaction vials. Reaction times refer to hold times at the selected set temperature, not to total irradiation times. Diethyl ether, tetrahydrofuran and 1,2-dimethoxyethane were distilled from sodium/benzophenone under argon over 4Å molecular sieves. All other solvents were distilled prior to use. *n*-Butyllithium was titrated with *N*-benzyl benzamide prior to use and stored under argon. Compounds ***o*5**, ***m*5**, ***p*5**^56^, and **6**^85,86^ were prepared according to known literature procedures. All other chemicals were purchased from commercial sources and used as received.

#### General procedure for the synthesis of ***o*1**, ***m*1** and ***p*1**

In a 10 mL Schlenk tube containing a stir bar, the respective iodo carborane (***o*5**, ***m*5** or ***p*** **5**) (81 mg, 0.3 mmol, 1.0 equiv.), 1,3-dihydro-2*H*-indol-2-one (40 mg, 0.3 mmol, 1.0 equiv.), potassium *tert*-butoxide (40 mg, 0.35 mmol, 1.2 equiv.), and methanesulfonato(2-dicyclohexylphosphino-2’-biphenyl-2-yl)palladium(II) (SPhosPdG4, 47 mg, 0.06 mmol, 5 mol%) were added. The vial was evacuated and refilled with argon three times. Then, 3 mL absolute 1,4-dioxane were added. The resulting mixture was stirred at $90\,^{\circ }\hbox {C}$ for 18 h, cooled to room temperature and diluted with 5 mL ethyl acetate. The black opaque mixture was filtered through a 1 cm pad of celite, washed with 3 mL water and 3 mL brine, dried over $\hbox {Na}_{2}\hbox {SO}_{4}$ and filtered. Silica (2 g) was added to the filtrate and the volatiles were removed under reduced pressure. The resulting dry load was directly used in a flash column chromatography (25 g silica, ethyl acetate/n-hexane gradient from 0% to 66%) to afford oxindoles ***o*1**, ***m*1** or ***p*1**.

#### 1-[9-(1,2-dicarba-*closo*-dodecaboranyl)]-1,3-dihydro-2*H*-indolin-2-one (***o*1**)

Colorless solid, 58% yield (48 mg, 0.17 mmol). Mp: $101\,^{\circ }\hbox {C}$. $^{1}$H NMR (400 MHz, DMSO) $\delta$ 7.33 (d, J = 8.1 Hz, 1H), 7.16 (q, J = 7.9, 7.3 Hz, 2H), 6.94 (t, J = 7.4 Hz, 1H), 5.07 (s, 1H), 4.94 (s, 1H), 3.49 (s, 2H), 3.37–1.30 (m, 9H). ^11^B{$^{1}$H} NMR (128 MHz, DMSO) $\delta$ 4.6 (s, 1B), − 4.9 (s, 1B), − 10.5 (s, 2B), − 15.1 (d, J = 209 Hz, 8B). ^11^B NMR (128 MHz, DMSO) $\delta$ 4.6 (s, 1B), − 4.9 (d, J = 147.7 Hz, 1B), − 10.5 (d, J = 148 Hz, 2B), − 15.8 (d, J = 187 Hz, 8B). $^{13}$C{$^{1}$H} NMR (101 MHz, DMSO) $\delta$ 179.1, 147.6, 127.3, 126.9, 124.4, 121.9, 112.1, 54.1, 50.6, 36.3. HR-ESI-MS (negative mode, ACN) m/z [M−H]^−^: calcd. for C_10_H_17_B_10_NO: 274.2246, found: 274.2242 with the expected isotopic distribution.

#### 1-[9-(1,7-dicarba-*closo*-dodecaboranyl)]-1,3-dihydro-2H-indolin-2-one (***m*1**)

Slightly yellow needles. 67% yield (55 mg, 0.20 mmol). Mp: $102-103\,^{\circ }\hbox {C}$. $^{1}$H NMR (400 MHz, DMSO) $\delta$ 7.43 (d, J = 8.0 Hz, 1H), 7.22 (ddd, J = 10.1, 5.3, 3.9 Hz, 2H), 7.03–6.95 (m, 1H), 4.06 (s, 2H), 3.56 (s, 2H), 2.50 (p, J = 1.8 Hz, 9H). ^11^B{$^{1}$H} NMR (128 MHz, DMSO) $\delta$ − 1.50 (s, 1B), − 7.7 (s, 2B), − 11.8 (s, 2B), − 14.5 (d, J = 192 Hz, 2B), − 18.4 (s, 2B). $^{13}$C{$^{1}$H} NMR (101 MHz, DMSO) $\delta$ 179.6, 147.6, 127.5, 127.0, 124.6, 122.2, 112.0, 53.9, 36.3. HR-ESI-MS (positive mode, ACN) m/z [M+H]$^{+}$: calcd. for C_10_H_17_B_10_NO: 276.2391, found: 276.2406 with the expected isotopic distribution.

#### 1-[9-(1,7-dicarba-*closo*-dodecaboranyl)]-carbazole **(8)**

When (2-dicyclohexylphosphino-2’,6’-dimethoxybiphenyl)[2-(2’-amino-1,1’-biphenyl)]palladium(II)-methansulfonat (SPhosPdG3, 47 mg, 0.06 mmol, 5 mol%) was used as a catalyst no oxindole-formation was observed after 18 h by TLC. Workup following the general procedure afforded carbazole **8** as red crystals in 5% yield with respect to the starting material ***o*5** or 100% yield calculated from the catalyst SPhosPdG3 (5 mg, 0.06 mmol). The physical data was identical to the data reported in the literature^101^.

#### 1-[2-(1,12-dicarba-*closo*-dodecaboranyl)]-1,3-dihydro-2*H*-indolin-2-one (***p*1**)

Slightly rose needles. 78% yield (64 mg, 0.23 mmol). Mp: $95-96\,^{\circ }\hbox {C}$. $^{1}$H NMR (400 MHz, DMSO) $\delta$ 7.64—7.49 (m, 1H), 7.32–7.25 (m, 2H), 7.07 (t, J = 7.5 Hz, 1H), 4.85 (s, 1H), 3.93 (s, 1H), 3.61 (s, 2H), 3.19–1.08 (m, 9H). ^11^B{$^{1}$H} NMR (128 MHz, DMSO) $\delta$ − 5.4 (s, 1B), − 15.9 (q, J = 243, 235 Hz, 9B). ^11^B NMR (128 MHz, DMSO) $\delta$ − 5.43 (s, 1B), − 8.90–25.6 (m, 9B). $^{13}$C{$^{1}$H} NMR (101 MHz, DMSO) $\delta$ 180.3, 146.4, 127.8, 126.9, 124.8, 122.9, 111.9, 64.3, 62.4, 36.7. HR-ESI-MS (positive mode, ACN) m/z [M+H]$^{+}$ calcd. for C_10_H_17_B_10_NO: 276.2391, found: 276.2400 with the expected isotopic distribution.

#### Sodium 1-[2-(7,8-dicarba-*nido*-undecaborate)]-1,3-dihydro-2H-indolin-2-one **(2)**

In a 10 mL Schlenk tube containing a stir bar, oxindole ***o*1** (134 mg, 0.5 mmol, 1.0 equiv.) was dissolved in 3 mL absolute ethanol and 2 mL water. Dry nitrogen was bubbled through the resulting solution with a Teflon canula for 30 min. The canula was removed and sodium fluoride (209 mg, 5.0 mmol, 10 equiv.) was added in one portion. The mixture was stirred at $90\,^{\circ }\hbox {C}$ for 18 h. The mixture was left to cool to room temperature 5 mL water were added and the resulting slurry was extracted with 10 mL ethyl acetate three times. The combined organic phases were dried over $\hbox {Na}_{2}\hbox {SO}_{4}$, filtered and the volatiles were removed under reduced pressure. The resulting solid was recrystallized from isopropanol (approx. 1,5 mL) to afford *nido*-compound **2** as long colorless needles in 76% yield (155 mg, 0.38 mmol).

Mp: $237\,^{\circ }\hbox {C}$. $^{1}$H NMR (400 MHz, DMSO) $\delta$ 7.69 (d, J = 8.4 Hz, 1H), 7.08–6.99 (m, 2H), 6.78 (t, J = 7.3 Hz, 1H), 4.34 (d, J = 4.2 Hz, 2H), 3.77 (pd, J = 6.2, 4.3 Hz, 2H), 3.23 (s, 2H), 1.80 (s, 2H), 1.04 (d, J = 6.1 Hz, 12H), 3.19–1.08 (m, 8H), − 2.70 (br s, 1H). ^11^B{$^{1}$H} NMR (128 MHz, DMSO) $\delta$ − 5.0 (s, 1B), − 11.4 (s, 1B), − 13.0 (s, 1B), − 18.9 (s, 2B), − 21.1–24.6 (m, 2B), − 32.3 (s, 1B), − 38.0 (s, 1B). ^11^B NMR (128 MHz, DMSO) $\delta$ − 5.0 (s, 1B), − 12.2 (t, J = 172 Hz, 2B), − 18.9 (d, J = 138 Hz, 2B), − 22.5 (d, J = 148 Hz, 2B), − 29.8–34.1 (m, 1B), − 37.9 (d, J = 141 Hz, 1B). $^{13}$C{$^{1}$H} NMR (101 MHz, DMSO) $\delta$ 178.7, 150.5, 126.5, 123.3, 120.1, 113.9, 62.5, 36.8, 26.0. HR-ESI-MS (negative mode, ACN) m/z [M−Na]−: calcd. for C_10_H_17_B_9_NNaO: 265.2197, found: 265.2176 with the expected isotopic distribution.

#### 1-(2,6-dichlorophenyl)-1,3-dihydro-2*H*-indolin-2-one **(CCF)**

In a 20 mL Schlenk tube containing a stir bar, diclofenac **(DCF)** (500 mg, 1.7 mmol, 1.0 equiv.) was dissolved in 3 mL ethyl acetate and 3 mL dichloromethane. One drop of *N*,*N*-dimethyl formamide was added to the tube and the mixture was cooled to $0\,^{\circ }\hbox {C}$ in an ice bath. Thionyl chloride (197 mg, 120 μL, 1.7 mmol, 1.0 equiv.) was slowly added *via* syringe over 5 min and the resulting solution was warmed to room temperature and stirred for 18 h. The resulting red solution was quenched with 5 mL water. The organic phase was separated and washed with 5 mL saturated aqueous NaHCO_3_ two times. The combined organic phases were dried over $\hbox {Na}_{2}\hbox {SO}_{4}$ and filtered. Silica (2 g) was added to the filtrate and the volatiles were removed under reduced pressure. The resulting dry load was directly used in a flash column chromatography (25 g silica, ethyl acetate/n-hexane gradient from 0 to 34%) to afford **CCF** as a light red solid in 99% yield (470 mg, 1.68 mmol).

The physical data was identical to the data reported in the literature^43^.

#### 2-[9-(1,2-dicarba-*closo*-dodecaboranyl-2-aminopheny)]acetonitrile **(9)**

In a 10 mL Schlenk tube containing a stir bar, 9-iodo-*ortho*-carborane (***o*5**) (81 mg, 0.3 mmol, 1.0 equiv.), 2-(2-aminophenyl) acetonitrile (40 mg, 0.3 mmol, 1.0 equiv.), potassium *tert*-butoxide (40 mg, 0.35 mmol, 1.2 equiv.), and methanesulfonato (2-dicyclohexylphosphino-2’-biphenyl-2-yl)palladium(II) (SPhosPdG4, 47 mg, 0.06 mmol, 5 mol%) were added. The Schlenk tube was evacuated and refilled with argon three times. Then, 3 mL absolute 1,4-dioxane were added. The resulting mixture was stirred at $90\,^{\circ }\hbox {C}$ for 18 h, cooled to room temperature and diluted with 5 mL ethyl acetate. The black opaque mixture was filtered through an 1 cm pad of celite, washed with 3 mL water and 3 mL brine, dried over $\hbox {Na}_{2}\hbox {SO}_{4}$ and filtered. Silica (2 g) was added and the volatiles were removed under reduced pressure. The resulting dry load was directly used in a flash column chromatography (25 g silica, dichloromethane/n-hexane gradient from 0% to 50%) to afford nitrile **9** as tan crystals in 58% yield (48 mg, 0.17 mmol). Mp: $97\,^{\circ }\hbox {C}$. $^{1}$H NMR (400 MHz, CDCl_3_) $\delta$ 7.25–7.12 (m, 4H), 6.75 (td, J = 7.3, 1.4 Hz, 1H), 3.53 (s, 2H), 3.48 (s, 1H), 3.41 (s, 1H). ^11^B NMR (128 MHz, CDCl_3_) $\delta$ 8.8 (s, 1B), − 3.4 (s, 1B), − 9.6 (s, 2B), − 12.2–19.9 (m, 6B). ^11^B NMR (128 MHz, CDCl_3_) $\delta$ 8.9 (s, 1B), − 3.4 (d, J = 149 Hz, 1B), − 9.6 (d, J = 150 Hz, 2B), − 12.5–19.3 (m, 6B). $^{13}$C{$^{1}$H} NMR (101 MHz, CDCl_3_) $\delta$ 145.7, 129.2, 128.9, 118.4, 117.2, 116.3, 116.0, 49.9, 42.3, 20.5. HR-ESI-MS (positive mode, ACN) m/z [M+H]$^{+}$: calcd. for C_10_H_18_B_10_$\hbox {N}_{2}$: 275.2551, found: 275.2544 with the expected isotopic distribution.

#### 1-[2-(7,8-dicarba-*nido*-undecaborate)]-$^{1}$H-indolium-2-amine **(3)**

In a 10 mL Schlenk tube containing a stir bar, nitrile **9** (198 mg, 0.72 mmol, 1.0 equiv.) was dissolved in 3 mL ethanol and 1 mL water. Dry nitrogen was bubbled through the resulting solution with a Teflon canula for 30 min. The canula was removed and sodium fluoride (150 mg, 3.6 mmol, 5.0 equiv.) was added in one portion. The mixture was stirred at $90\,^{\circ }\hbox {C}$ for 18 h. The mixture was left to cool to room temperature; 5 mL water were added resulting in a massive off-white precipitate. The suspension turned clear again upon addition of 10 mL ethyl acetate. The phases were separated and the mixture was extracted with 10 mL ethyl acetate three times. The combined organic phases were dried over $\hbox {Na}_{2}\hbox {SO}_{4}$ and filtered. Silica (2 g) was added to the filtrate and the volatiles were removed under reduced pressure. The resulting dry load was directly used in a flash column chromatography (25 g silica, ethyl acetate/n-hexane gradient from 15% to 40%) to afford amidine **3** as a white powder in >99% yield (190 mg, 0.72 mmol). Mp: $217\,^{\circ }\hbox {C}$. $^{1}$H NMR (400 MHz, DMSO) $\delta$ 10.08 (s, 1H), 8.42 (s, 1H), 8.04 (d, J = 8.3 Hz, 1H), 7.36 - 7.23 (m, 2H), 7.11 (t, J = 7.4 Hz, 1H), 4.14 (s, 2H), 2.18 (s, 1H), 2.11 (s, 1H), − 2.46 (br. s, 1H). ^11^B{$^{1}$H} NMR (128 MHz, DMSO) $\delta$ − 3.9 (s, 1B), − 11.5 (s, 1B), − 13.2 (s, 1B), − 18.8 (s, 1B), − 20.7 (s, 1B), − 22.0 (s, 1B), − 23.9 (s, 1B), − 32.9 (s, 1B), − 38.5 (s, 1B). ^11^B NMR (128 MHz, DMSO) $\delta$ − 3.9 (s, 1B, − 12.3 (t, J = 179 Hz, 2B), − 16.3–28.7 (m, 4B), − 32.9 (d, J = 133 Hz, 1B), − 38.5 (d, J = 142 Hz, 1B). $^{13}$C{$^{1}$H} NMR (101 MHz, DMSO) $\delta$ 174.92, 148.65, 127.52, 127.43, 124.17, 124.06, 116.50, 37.12. HR-ESI-MS (positive mode, ACN) m/z [M+H]$^{+}$: calcd. for C_10_H_17_B_9_$\hbox {N}_{2}$: 266.2502, found: 266.2491 with the expected isotopic distribution.

### Biological evaluation

#### Materials and methods

Materials: The culture media DMEM (Dulbecco’s Modified Eagle Medium) and RPMI-1640 (Roswell Park Memorial Institute-1640) were purchased from Capricorn Scientific GmbH (Ebsdorfergrund, Germany). The penicillin/streptomycin solution was obtained from Biological Industries (Cromwell, CT, USA). Fetal calf serum (FCS), phosphate-buffered saline (PBS), carboxyfluorescein diacetate succinimidyl ester (CFSE), dimethyl sulfoxide (DMSO), trypsin, crystal violet (CV), and propidium iodide (PI) were bought from Sigma-Aldrich (St. Louis, MO, USA). 3-(4,5-Dimethythiazol-2-yl)-2,5-diphenyltetrazolium bromide (MTT) was purchased from AppliChem (St. Louis, MO, USA). Annexin V-FITC (AnnV-FITC) was obtained from BD Pharmingen (San Diego, CA, USA) and ApoStat from R&D Systems (Minneapolis, MN, USA). Dihydrorhodamine 123 (DHR 123) was obtained from Thermo Fisher Scientific (Waltham, MA, USA). Paraformaldehyde (PFA) was purchased from Serva (Heidelberg, Germany). Acridine orange (AO) was obtained from LaboModerne (Paris, France).

All compound stocks were made in DMSO at a concentration of 100 mM and stored at $-20\,^{\circ }\hbox {C}$ for up to 2 weeks. The working concentrations were freshly prepared in a culture medium immediately before treatment.

The biological study was performed on a murine colon adenocarcinoma cell line (MC38), two human colorectal carcinoma cell lines (HCT116 and HT29), and a human fetal lung fibroblast cell line (MRC-5). All cell lines are commercially available and were obtained from the American Type Culture Collection (ATCC). The human cell lines were kept in HEPES-buffered RPMI-1640 medium supplemented with 10$\%$ heat-inactivated FCS, 2 mM L-glutamine, 0.01$\%$ sodium pyruvate, penicillin (100 units mL^−1^), and, streptomycin ($100\,\upmu \hbox {g}$ mL^-1^). The MC38 cell line was maintained in DMEM medium additionally supplemented with 1$\%$ non-essential amino acids. Cells were grown at $37\,^{\circ }\hbox {C}$ in a humidified atmosphere with 5$\%$ $\hbox {CO}_{2}$. Peritoneal exudate cells (PECs) were isolated from healthy C57BL/6 mice (males, n = 3, 3 months old, weight 22–25 g) by performing an ice-cold PBS peritoneal lavage. Mice were sacrificed by cervical dislocation. Cells were cultivated for 4 h when nonadherent population was removed by washing in PBS, and additionally cultivated overnight in HEPES-buffered RPMI-1640 medium supplemented with 5$\%$ heat-inactivated FCS before treatment with experimental drugs. Animal handling and protocol for isolation of PECs were performed according to European Union rules, and the ARRIVE guidelines 2.0. Experiments were approved by the Institutional Animal Care and Use Committee at the Institute for Biological Research “Siniša Stanković” (IBISS) and by the national licensing committee at Department of Animal Welfare, Veterinary Directorate, Ministry of Agriculture, Forestry and Water Management of the Republic of Serbia (No. 323-07-02147/2023-05)^102^.

For the viability tests, MC38, HCT116, and HT29 cells were seeded in 96-well plates at a density of 2$\times$103, 5$\times$103, and 6$\times$103 cells/well, respectively. To determine whether the drug affected the viability of healthy, non-malignant cells, MRC-5 and PECs were seeded in 96-well plates at a density of 8$\times$103 and 2$\times$105 cells/well, respectively. For the flow cytometric analyses, MC38 cells were seeded in 6-well plates at 5$\times$104 cells/well. For morphological assessment of nuclei and the detection of autophagy, MC38 cells were seeded in 2-well chamber slides at 1.5$\times$104 cells/well.

#### Viability assays

All cell lines were treated with a wide range of concentrations (1.56 - 100 $\upmu \hbox {M}$) of **DCF**, **CCF**, ***o*1**, ***m*1**, ***p*1**, **2** and **3**. After 72 h, cell viability was determined using MTT and CV tests. First, the supernatant from the wells was discarded and the cells were incubated with an MTT solution (0.5 mg mL^−1^). After half an hour, the purple formazan crystals formed were dissolved in DMSO. For CV staining of the cells, the cells were fixed with 4$\%$ PFA for 10 min and then stained with a 0.02$\%$ CV solution for 15 min, all at room temperature (RT). The cells were then washed with tap water, air-dried and the dye was dissolved in 33$\%$ acetic acid. For both viability tests, the absorbance of the developed colors was measured using an automated microplate reader at 570 nm with a reference wavelength of 670 nm. The results were expressed as a percentage of the control values of the untreated cells, which were arbitrarily set at 100$\%$. IC_50_ values, defined as 50$\%$ inhibitory concentration, were calculated using a four-parameter logistic function and presented as mean±SD. All biological experiments were performed in triplicates. To evaluate the nature of the detected autophagy, a combined treatment with compound **3** and the autophagy inhibitors chloroquine (Chlq) and 3-methyladenine (3-MA) was carried out. MC38 cells were seeded overnight and treated with the IC_50_ concentration of compound **3** and Chlq (20 μM) or 3-MA (1 mM) simultaneously. After 72 h, cell viability was determined by CV assay.

#### Flow cytometric analyses

For all flow cytometric analyses, MC38 cells were seeded overnight and then treated with the IC_50_ concentration of compound **3** for 72 h. The cells were harvested, washed, and stained according to the manufacturer’s instructions for each method. To determine whether the compound induces apoptosis, cells were double stained with AnnV-FITC and PI (15 μg mL^–1^) for 15 min at RT in the dark. To detect caspase activity, cells were stained with the FITC-conjugated caspase inhibitor ApoStat for 30 min at $37\,^{\circ }\hbox {C}$. To detect cell proliferation, MC38 cells were stained with CFSE (1 μM) for 10 min at $37\,^{\circ }\hbox {C}$ prior to seeding and then treated as previously described. To detect intracellular production of Reactive oxygen/nitrogen Species (ROS/RNS), MC38 cells were prestained with DHR 123 (1 $\upmu$ M) for 20 min at $37\,^{\circ }\hbox {C}$, then seeded and exposed to an IC_50_ concentration of compound **3**. After 72 h incubation, all samples were washed, resuspended in PBS and fluorescence intensity was analyzed using a CytoFLEX$^{\circledR }$ flow cytometer (Beckman Coulter, Pasadena, CA, USA), while the data obtained was evaluated using the $\hbox {FlowJo}^{\textrm{TM}}$ software program.

#### Fluorescence microscopy

For morphological assessment of cell nuclei, MC38 cells were seeded overnight in 2-well chamber slides and then treated with the IC_50_ concentration of compound **3** for 72 h. At the end of the incubation period, cells were fixed with 4$\%$ PFA for 15 min at RT. Staining of cells was performed with PI solution (0.1$\%$ Triton X-100, 0.5 M EDTA pH 8, 50 μg mL^− 1^ RNase, and 50 $\upmu \hbox {g}$ mL^− 1^ PI - final concentrations in PBS) for 1–2 min at RT. After washing in PBS, the cells were mounted in a fluorescence microscopy medium (Fluoromount-$\hbox {G}^{\textrm{TM}}$, eBioscience, San Diego, CA, USA). The slides were analyzed with a ZeissAxio Observer Z1 inverted fluorescence microscope (Carl Zeiss AG, Oberkochen, Germany) at 400$\times$ magnification. For the detection of autophagosomes, AO staining of cells seeded in 2-well chamber slides was applied. After 72 h treatment with the IC_50_ concentration of compound **3**, the cells were incubated for 15 min at $37\,^{\circ }\hbox {C}$ in 10 $\upmu$ M AO solution (in PBS). The dye was then removed, the cells were rinsed with PBS and immediately evaluated *via* fluorescence microscopy at 400$\times$ magnification using the Leica DM4 B microscope equipped with a DFC7000 T camera (Leica Microsystems CMS GmbH, Wetzlar, Germany).

#### Statistical analysis

Data were presented as mean±SD of at least three independent experiments. Differences between groups were analyzed using Student’s *t*-test and *p*-values of less than 0.05 were considered statistically significant.

#### COX inhibition assay

The COX inhibition activity against ovine COX-1 and human recombinant COX-2 was determined using the COX Fluorescent Inhibitor Screening Assay Kit (Cayman Chemical Company, Ann Arbor, MI, USA) according to the manufacturer’s instructions as reported^56^. All compounds were screened at a concentration of 100 $\upmu \hbox {M}$ in duplicate. Compounds **2** and **3** were assayed in a concentration range from 0.1 to 100 $\upmu$ M in duplicate for determination of the IC_50_ values. IC_50_ values were determined with GraphPad Prism 10 version 10.2.3 by fitting to the equation y = A2+(A1-A2)/(1+(x/x0)p) and are given as absolute IC_50_ values. COX-2 selective inhibitor celecoxib and COX-1 selective inhibitor **SC-560** served as reference compounds (see Table 1).

## Supplementary Information

Supplementary Information.

## Data Availability

Crystallographic data for the structures reported in this article have been deposited at the Cambridge Crystallographic Data Centre. Copies of the data can be obtained free of charge via https://www.ccdc.cam.ac.uk/structures/. All other relevant data generated and analysed during this study, which include experimental, spectroscopic, crystallographic and computational data, are included in this article and its Supporting Information files. The authors declare that the data supporting the findings of this study are available within the paper and its Supporting Information files. Should any raw data files be needed in another format they are available from the corresponding author upon reasonable request.
