# Supplementary material for: Re-design and evaluation of diclofenac-based carborane-substituted prodrugs and their anti-cancer potential
Source: Sci Rep. 2024 Dec 16;14:30488. doi: 10.1038/s41598-024-81414-x (PMC11649784; doi:10.1038/s41598-024-81414-x)
Supplement: Supplementary file 1 — Supplementary Information. [file 41598_2024_81414_MOESM1_ESM.pdf]

Re-Design And Evaluation Of Diclofenac-Based Carborane-Substituted Prodrugs And Their Anti-Cancer Potential

Christoph Selg,[1] Vuk Gordić,[2] Tamara Krajnović,[2] Antonio Buzharevski,[3] Markus Laube,[4] Aleksandr Kazimir,[5] Peter Lönnecke,[1] Mara Wolniewicz,[6] Menyhárt B. Sárosi,[3] Jonas Schädlich,[4,7] Jens Pietzsch,[4,7] Sanja Mijatović,[2] Danijela Maksimović-Ivanić,[2] Evamarie Hey-Hawkins*[1]

[1] Department of Chemistry and Mineralogy, Leipzig University, Institute of Bioanalytical Chemistry, Deutscher Platz 5.

[2] Department of Immunology, Institute for Biological Research "Siniša Stanković" - National Institute of the Republic of Serbia, University of Belgrade, Bulevar despota Stefana 142, 11108 Belgrade, Serbia.

[3] Department of Chemistry and Mineralogy, Leipzig University, Institute of Inorganic Chemistry, Johannisallee 29, 04103 Leipzig, Germany

[4] Department of Radiopharmaceutical and Chemical Biology, Helmholtz-Zentrum Dresden-Rossendorf, Bautzner Landstraße 400, 01328 Dresden, Germany.

[5] Institute for Drug Discovery, Leipzig University, Brüderstraße 34, 04103, Leipzig, Germany.

[6] Department of Chemistry and Mineralogy, Leipzig University, Institute of Organic Chemistry, Johannisallee 29, 04103 Leipzig, Germany.

[7] Faculty of Chemistry and Food Chemistry, School of Science, Technische Universität Dresden, Mommsenstraße 4, 01069 Dresden, Germany

E-mail: hey@uni-leipzig.de

Supporting Information

Table of Contents

1 General procedures for the acidic and alkaline ring opening attempts 2

2 Stability Tests for Compound **3** 3

3 Biological Evaluation 4

4 Single crystal X-ray diffraction 7

5 DFT Calculations 12

6 Molecular docking 13

7 NMR Data for Compounds **6**, **7**, ***o*1**, ***m*1**, ***p*1**, **2**, **3**, and **9** 17

8 HR-ESI MS Data for Compounds ***o*1**, ***m*1**, ***p*1**, **2**, **3** and **9** 32

9 HPLC Data for compounds ***o*1**, ***m*1**, ***p*1**, **2**, **3** and **CCF** 35

1 General procedures for the acidic and alkaline ring opening attempts

In a 10 mL microwave tube containing a stir bar, oxindole ***p1*** (83 mg, 0.5 mmol, 1.0 equiv.) was dissolved in 3 mL of the respective solvent or solvent mixture. Dry nitrogen was bubbled through the resulting solution with a Teflon canula for 30 minutes. The canula was removed and the acid or base was added in one portion. The tube was sealed with a PTFE (polytetrafluoroethylene) seal and the mixture was stirred at the temperatures given in Table Table S 1 for 18 hours. The reaction was monitored by TLC.

*Table S 1. Experimental conditions in the attempts to hydrolyze the oxindole ring of **p1**.*

Acid or Base	Concentration of Acid or Base	Solvents (v/v)	Temperature [°C]
NaOH	5 M	H_2O/EtOH (1:3)	90
NaOH	5 M	H_2O/THF (1:3)	80
KOH	5 M	H_2O/EtOH (1:3)	90
KOH	5 M	H_2O/THF (1:3)	80
CsOH	5 M	H_2O/EtOH (1:3)	90
CsOH	5 M	H_2O/THF (1:3)	80
HCl	1 M	H_2O/EtOH (1:3)	90
HCl	5 M	H_2O/EtOH (1:3)	90
HCl	5 M	H_2O/EtOH (1:3)	90
H_2SO_4	5 M	H_2O with 2% v/v AcOH	100

In the case of cesium hydroxide and H_2SO_4, complete conversion was observed by TLC. The reaction mixture was cooled to room temperature, extracted with 10 mL dichloromethane three times and the volatiles were removed under reduced pressure. In the ^{1}H NMR spectrum, ring-closure could be observed already after two minutes and a purification was not possible (Figure Figure S 1).

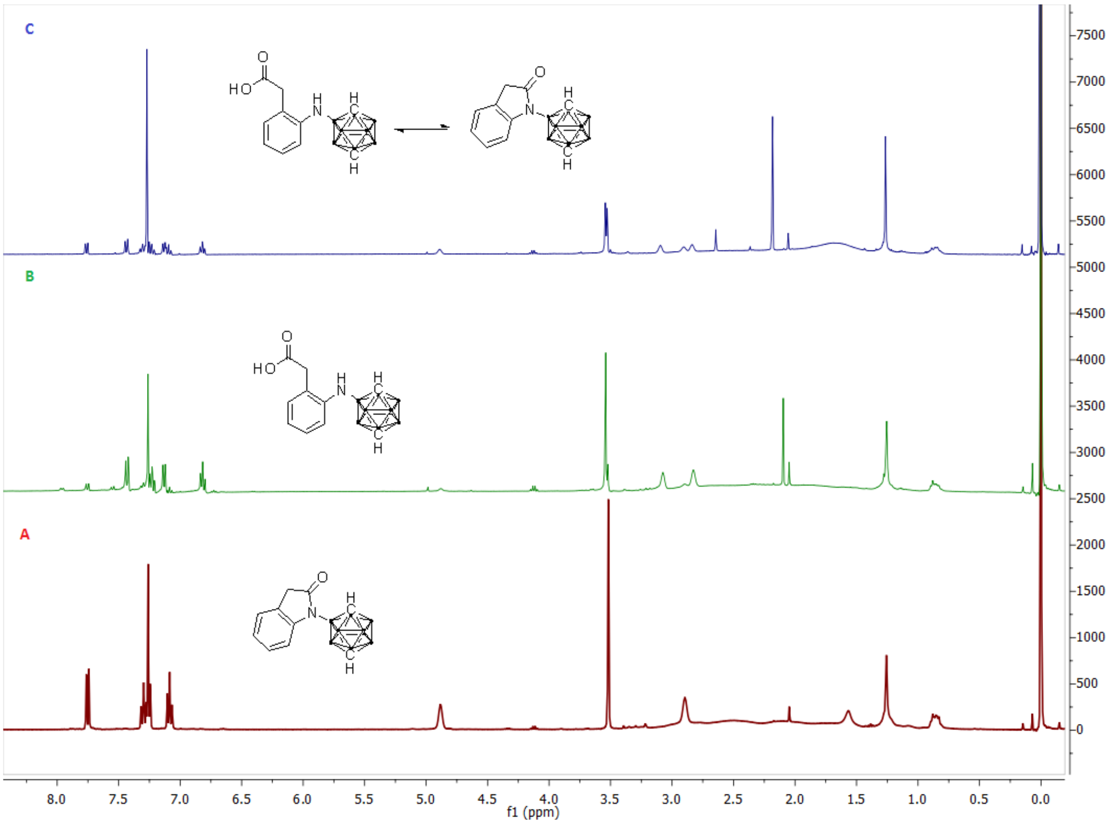

*Figure S 1. ^{1}H NMR spectra of the ring closing of the carboranyl analogue of diclofenac; A: **p1**; B: p-carborane derivative of **DCF**; C: a mixture of **p1** and p-carborane derivative of **DCF**.*

2 Stability Tests for Compound 3

In a 1 mL Eppendorf vial, 10 µL (0.05 µmol) of a freshly prepared 5 mM stock solution of compound **3** in DMSO was mixed with 490 µL of DMEM (Dulbecco's Modified Eagle Medium) containing 10% of fetal bovine serum and stored in an incubation oven at 40 °C for a total 72 hours. After 0, 24 and 72 hours, a 50 µL sample was collected from the mixture and diluted with 250 µL acetonitrile in a 500 µL Eppendorf vial to precipitate the proteins. The mixture was vortexed for 30 s before it was centrifuged for 20 min at 15,000 min^{-1} and 4 °C. 100 µL of the supernatant solution was transferred into a HPLC vial with a pipette and the vial was screw-sealed with a rubber septum. 5 µL of the solution were injected into the HPLC-MS system. The chromatograms after 0, 24 and 72 hours showed no signs of decomposition, and after 72 hours the purity (relative area of integrals) was still above 95%.

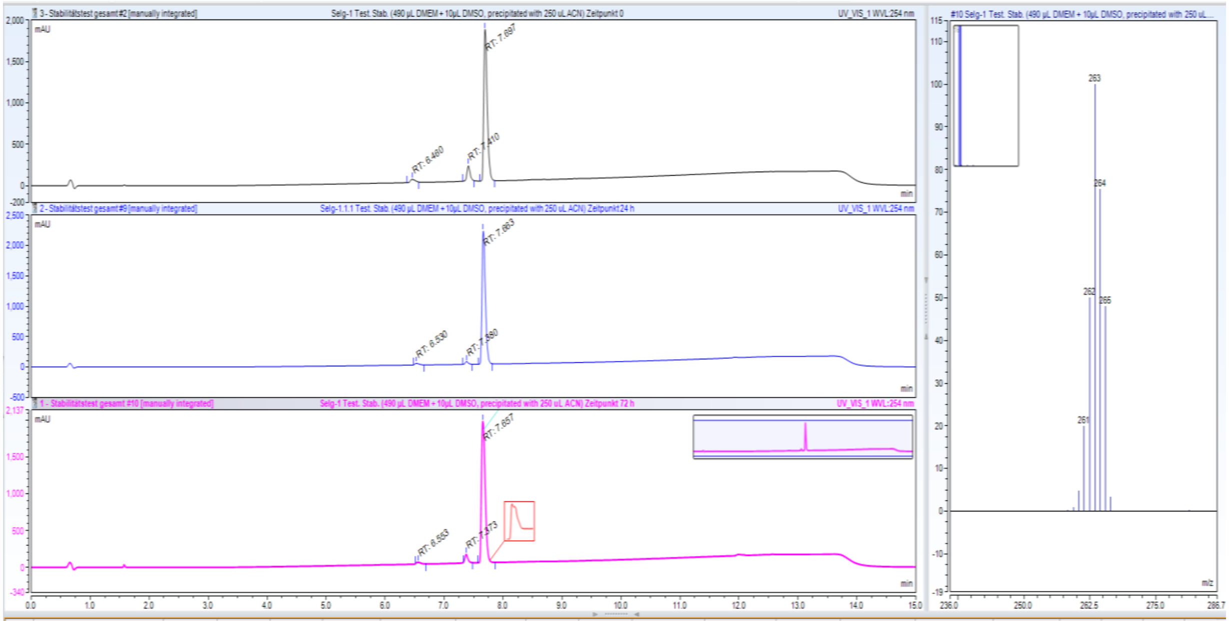

	A	B	C	D	E	F	G
1	Peak Name	Ret. Time	Area	Rel Area	Amount	Height	
2		min	mAU*min	%	n.a.	mAU	
4		6.55	1	0.75	n.a.	17.114	
5		7.37	6	4.00	n.a.	110.208	
6		7.66	141	95.24	n.a.	1920.612	

*Figure S 2. Chromatograms to monitor the stability of compound **3** in the culture medium. Samples measured at 0, 24 and 72 hours (top to bottom) including the mass spectrum (right) and integration data (bottom).*

3 Biological Evaluation

*Table S 2. Selectivity index for compound **3** (S.I.; IC_{50} value of primary vs. cancer cells).*

Primary cell lines	Cancer cell lines		
**MRC-5**	1.81	1.5	0.98
**PEC**	2.63	2.17	1.42

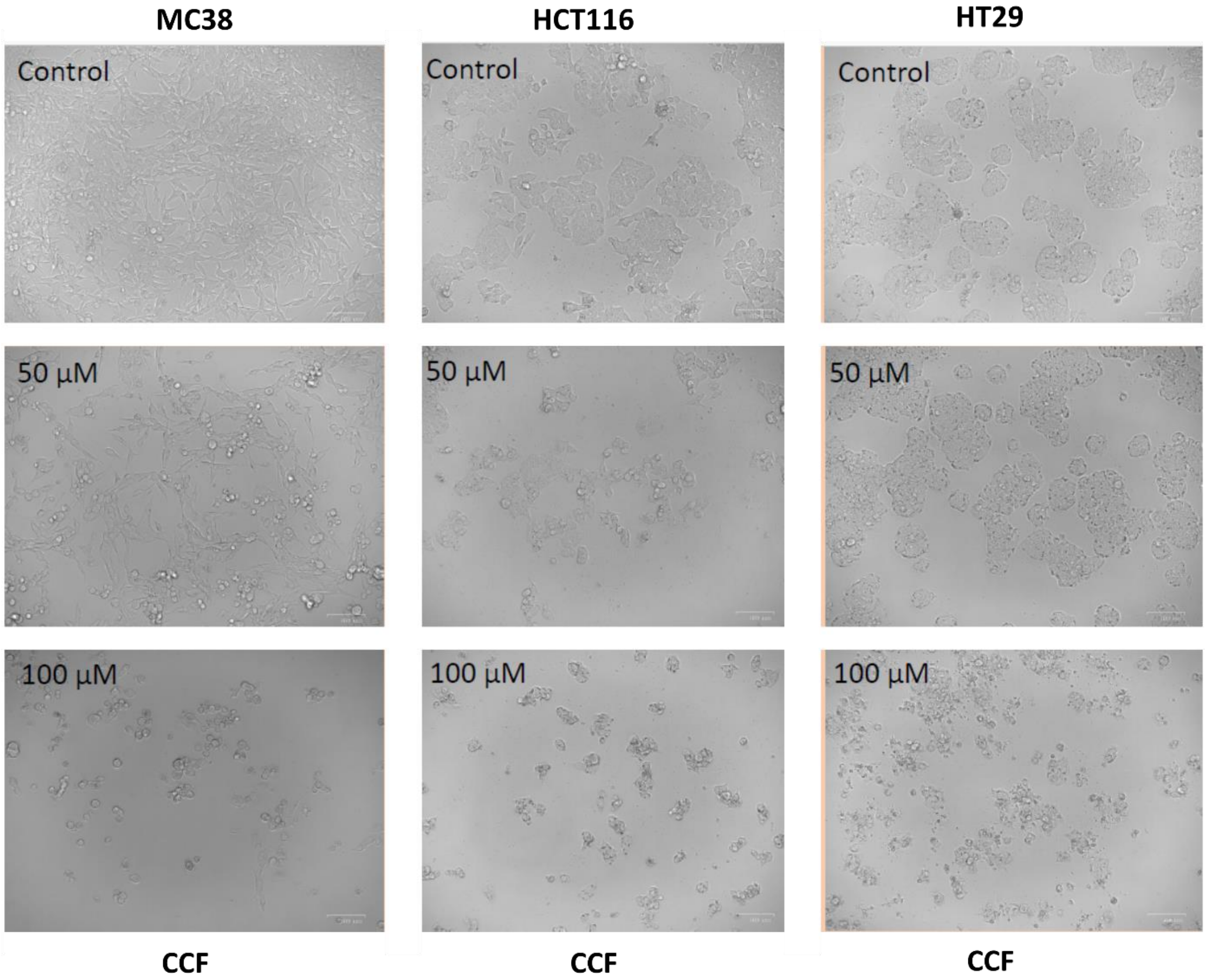

*Figure S 3. Photomicrographs of the indicated cells after treatment with the indicated concentration of **CCF** for 72 hours.*

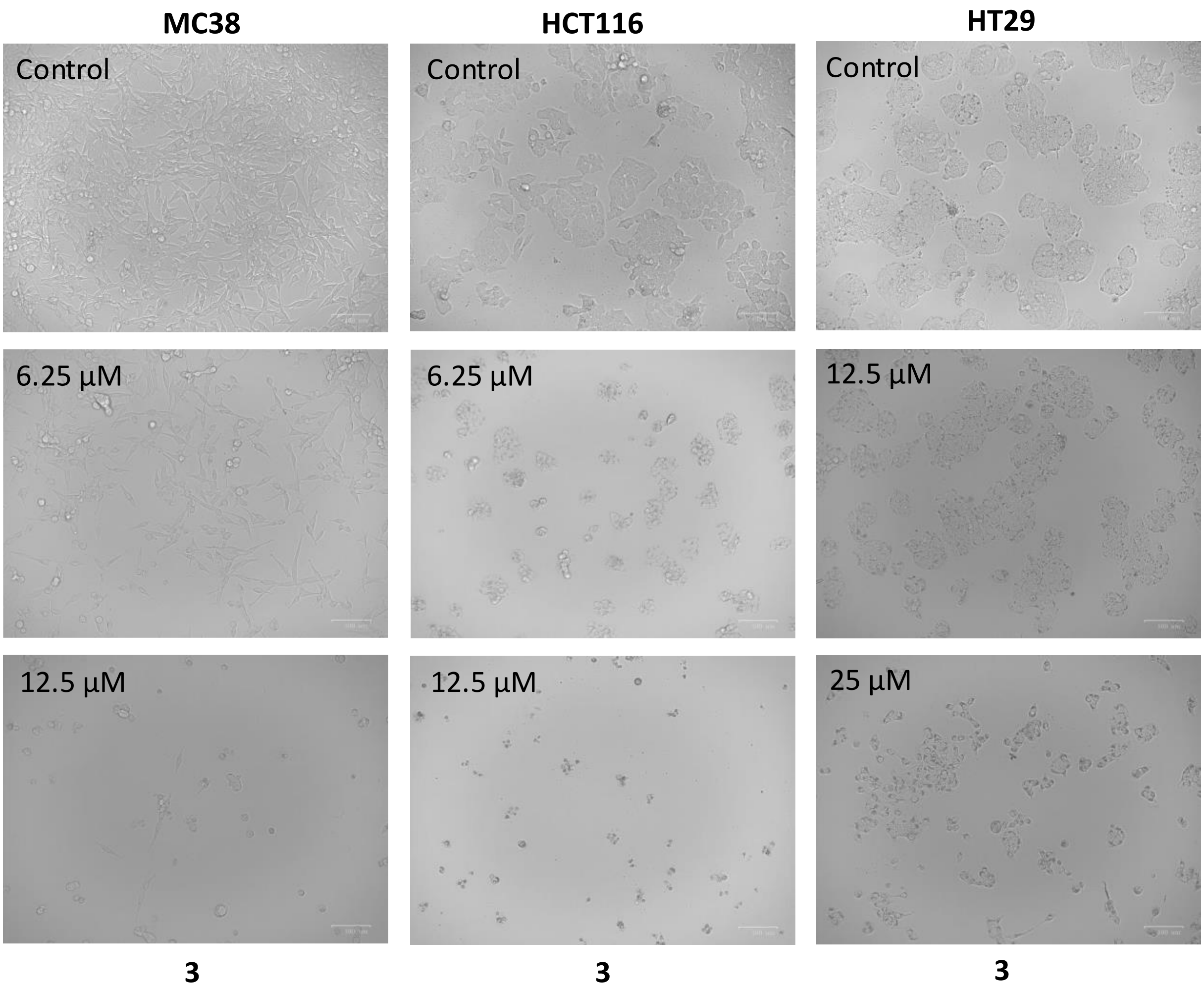

*Figure S 4 Photomicrographs of the indicated cells after treatment with the indicated concentration of compound **3** for 72 hours.*

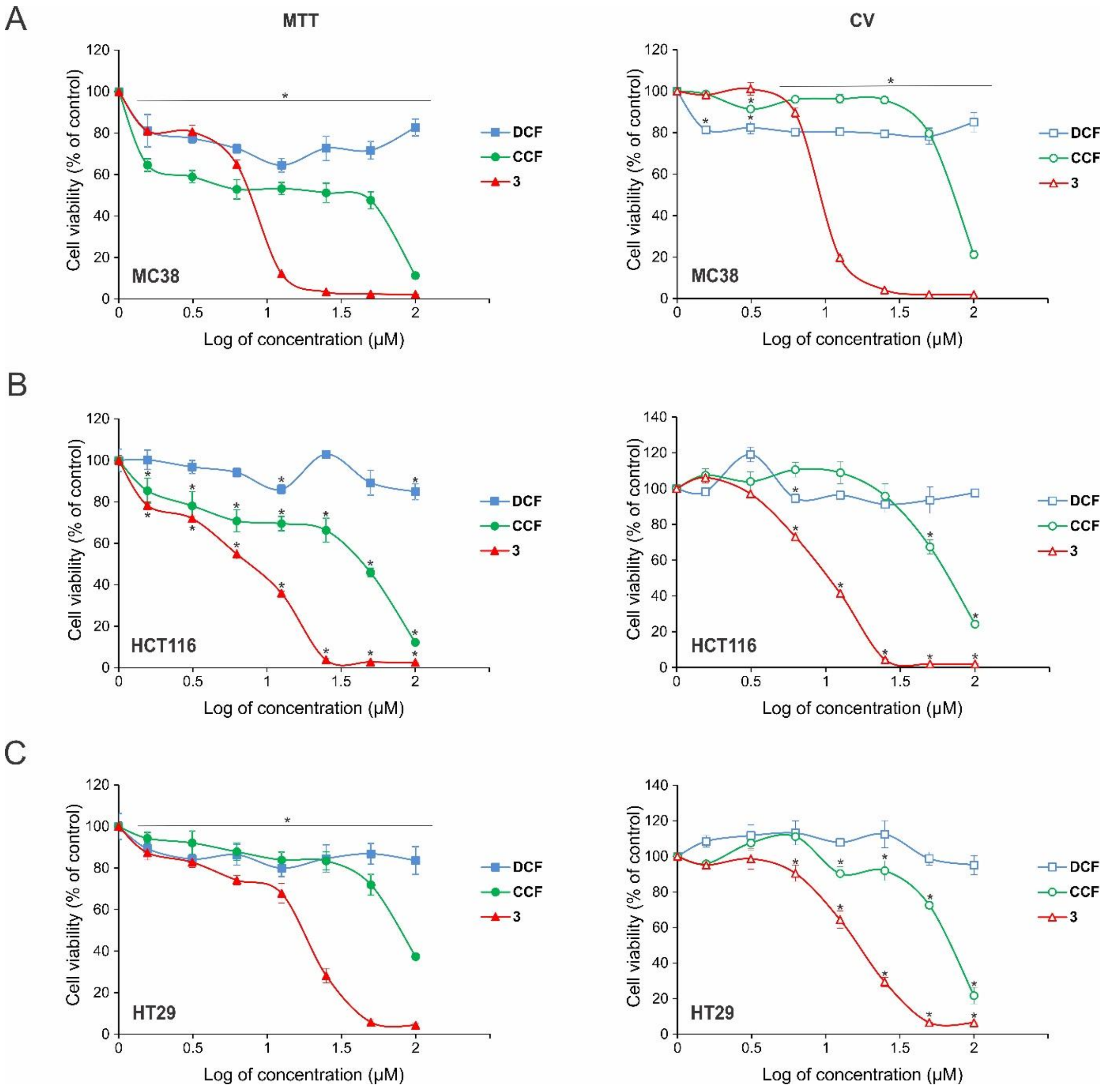

Figure S 5. Cell viability curves for ***DCF, CCF*** *and compound* ***3*** *using MTT or CV dye.*

4 Single crystal X-ray diffraction

The data were collected on a Gemini diffractometer (Rigaku Oxford Diffraction) using Mo-Kα radiation and ω-scan rotation. Data reduction was performed with CrysAlisPro[1] including the program SCALE3 ABSPACK for an empirical absorption correction. All structures were solved by dual space methods with SHELXT[2] and the refinement was performed with SHELXL[3]. With the exception of (**9**) and disordered atoms of a structure, hydrogen atoms are generally located with a difference-density Fourier map. Carbaborane carbon atoms could be localized for all compounds with a bond length and displacement parameter analysis. Structure figures were generated with DIAMOND-4.[4]

CCDC deposition numbers given in Table S 3 and Table S 4 contain the supplementary crystallographic data for this paper. These data can be obtained free of charge via https://summary.ccdc.cam.ac.uk/structure-summary-form (or from the Cambridge Crystallographic Data Centre, 12 Union Road, Cambridge CB2 1EZ, UK; fax: (+44)1223-336-033; or deposit@ccdc.cam.uk).

[1] Rigaku Corporation. CrysAlisPro Software System. Rigaku Oxford Diffraction; Wroclaw, Poland: 1995–2023.
[2] Sheldrick G.M. SHELXT–integrated space-group and crystal-structure determination. Acta Crystallogr. A Found. Adv. 2015;71:3–8.
[3] Sheldrick G.M. Crystal structure refinement with SHELXL. Acta Crystallogr. C Struct. Chem. 2015;71:3–8.
[4] Crystal Impact GbR. Brandenburg, K; Bonn, Germany: version 4.6.8. DIAMOND 4.

Table S 3. Fundamental structure parameters of ***o1****,* ***m1*** *and* ***p1****.*

Compound	**o1**	**m1**	**p1**
Empirical formula	$C_{10}H_{17}B_{10}NO$	$C_{10}H_{17}B_{10}NO$	$C_{10}H_{17}B_{10}NO$
Formula weight	275.35	275.35	275.35
Temperature [K]	130(2)	130(2)	130(2)
Wavelength [pm]	71.073	71.073	71.073
Crystal system	Orthorhombic	Orthorhombic	Orthorhombic
Space group	$P\ 2_12_12_1$	$P\ 2_12_12_1$	$P\ 2_12_12_1$
Unit cell dimensions			
a [pm]	700.94(2)	698.84(3)	696.10(2)
b [pm]	859.65(2)	861.11(3)	868.65(3)
c [pm]	2397.26(6)	2409.44(7)	2405.92(8)
α [deg]	90	90	90
β [deg]	90	90	90
γ [deg]	90	90	90
Volume [nm^3]	1.44450(6)	1.44995(9)	1.45478(8)
Z	4	4	4
$\rho_{(calculated)}$ [Mg/m^3]	1.266	1.261	1.257
µ [mm^{-1}]	0.068	0.067	0.067
F(000)	568	568	568
Crystal size [mm^3]	0.47 · 0.11 · 0.08	0.3 · 0.3 · 0.05	0.60 · 0.30 · 0.10
Θ_{Min} / Θ_{Max} [deg]	2.517 / 32.596	2.512 / 30.545	2.493 / 32.263
Index ranges	$-10 \leq h \leq 10$<br>$-12 \leq k \leq 12$<br>$-34 \leq l \leq 36$	$-9 \leq h \leq 9$<br>$-11 \leq k \leq 11$<br>$-33 \leq l \leq 19$	$-10 \leq h \leq 9$<br>$-12 \leq k \leq 12$<br>$-29 \leq l \leq 35$
Reflections collected	22082	12788	15928
Indp. reflections (R_{int})	4889 (0.0541)	3946 (0.0541)	4817 (0.0299)
Completeness (Θ_{Max})	99.9 % (30.51)	100.0 % (28.29)	100.0 % (30.51)
T_{Max} / T_{Min}	1.00000 / 0.98944	1.00000 / 0.98253	1.00000 / 0.87832
Restraints / parameters	0 / 267	0 / 267	0 / 267
Gof on F^2	1.057	1.024	1.043
R1 / wR2 (I>2σ(I))	0.0527, 0.1208	0.0532, 0.1107	0.0428, 0.1071
R1 / wR2 (all data)	0.0695, 0.1309	0.0699, 0.1185	0.0489, 0.1111
Absolute structure parameter	0.3(7)	-0.2(9)	-0.4(5)
Residual electron density [e·Å^{-3}]	0.392 / -0.363	0.346 / -0.190	0.327 / -0.251
Comments	†[1]	†[1]	†[1]
CCDC No	2372648	2372649	2372650

*Table S 4. Fundamental structure parameters of **2**, **3**, **8** and **9**.*

Compound	**2**	**3**	**8**	**9**
Empirical formula	$C_{32}H_{66}B_{18}N_2Na_2O_6$	$C_{10}H_{19}B_9N_2$	$C_{14}H_{19}B_{10}N$	$C_{10}H_{18}B_{10}N_2$
Formula weight	815.42	264.56	309.40	274.36
Temperature [K]	130(2)	130(2)	130(2)	130(2)
Wavelength [pm]	71.073	71.073	71.073	71.073
Crystal system	Orthorhombic	Orthorhombic	Monoclinic	Triclinic
Space group	P bca	P nma	P 2_1/n	P $\bar{1}$
Unit cell dimensions				
a [pm]	1248.67(2)	2381.62(5)	1414.81(7)	1303.04(2)
b [pm]	2022.16(4)	707.29(2)	786.59(3)	1361.93(2)
c [pm]	1831.98(4)	833.26(2)	1516.46(8)	1730.49(3)
α [deg]	90	90	90	96.866(1)
β [deg]	90	90	97.538(5)	91.031(1)
γ [deg]	90	90	90	97.172(1)
Volume [nm^3]	4.62577(16)	1.40362(6)	1.67305(14)	3.02344(8)
Z	4	4	4	8
$\rho_{(calculated)}$ [Mg/m^3]	1.171	1.252	1.228	1.205
μ [mm^{-1}]	0.086	0.064	0.062	0.061
F(000)	1728	552	640	1136
Crystal size [mm^3]	0.32 · 0.22 · 0.08	0.66 · 0.16 · 0.16	0.35 · 0.20 · 0.10	0.42 · 0.39 · 0.25
Θ_{Min} / Θ_{Max} [deg]	2.216 / 30.678	2.590 / 32.357	2.112 / 28.324	2.325 / 32.475
	-17 ≤ h ≤ 15	-35 ≤ h ≤ 35	-18 ≤ h ≤ 18	-18 ≤ h ≤ 19
Index ranges	-28 ≤ k ≤ 27	-10 ≤ k ≤ 10	-10 ≤ k ≤ 10	-20 ≤ k ≤ 19
	-25 ≤ l ≤ 25	-11 ≤ l ≤ 12	-20 ≤ l ≤ 19	-25 ≤ l ≤ 24
Reflections collected	48478	25154	4952	72008
Indp. reflections (R_{int})	6674 (0.0590)	2546 (0.0306)	4952 (0.0515)	20121 (0.0460)
Completeness (Θ_{Max})	100.0 % (28.29)	100.0 % (30.51)	100.0 % (26.38)	99.9 % (30.51)
T_{Max} / T_{Min}	1.00000 / 0.92562	1.00000 / 0.93387	1.00000 / 0.95451	1.00000 / 0.99329
Restraints / parameters	18 / 405	160 / 191	0 / 303	7 / 862
Gof on F^2	1.227	1.152	0.939	1.015
R1 / wR2 (I>2σ(I))	0.0918, 0.1771	0.0569, 0.1376	0.0483, 0.0955	0.0600, 0.1386
R1 / wR2 (all data)	0.1118, 0.1839	0.0619, 0.1406	0.0725, 0.1010	0.0908, 0.1550
Absolute structure parameter	-		-	-
Residual electron density [e·Å^{-3}]	0.329 / -0.282	0.368 / -0.256	0.194 / -0.231	0.375 / -0.285
Comments	†[2]	†[3]	†[4]	†[5]
CCDC No	2372651	2372652	2372653	2372654

†[1]: The *ortho*, *meta* and *para* carbaboranes are isotyp. †[2]: Disordered carbaborane (0.722(9):0.278(9)) and *i*-propanol molecule (0.53(2):0.47(2)). The carbaborane disorder could be detected and refined only for C(1)-H(1) with a reasonable accuracy. †[3]: Carbaborane moiety highly disordered on a special position (mirror plane). †[4]: Two component twin. Twin law by rows: -1.00 0.00 0.00, 0.00 -1.00 0.00, 0.28 0.00 1.00. Twin domain ratio 0.579(1):0.421(1). †[5]: Disordered nitrile substituent (C40-N8) in the vicinity of a centre of inversion.

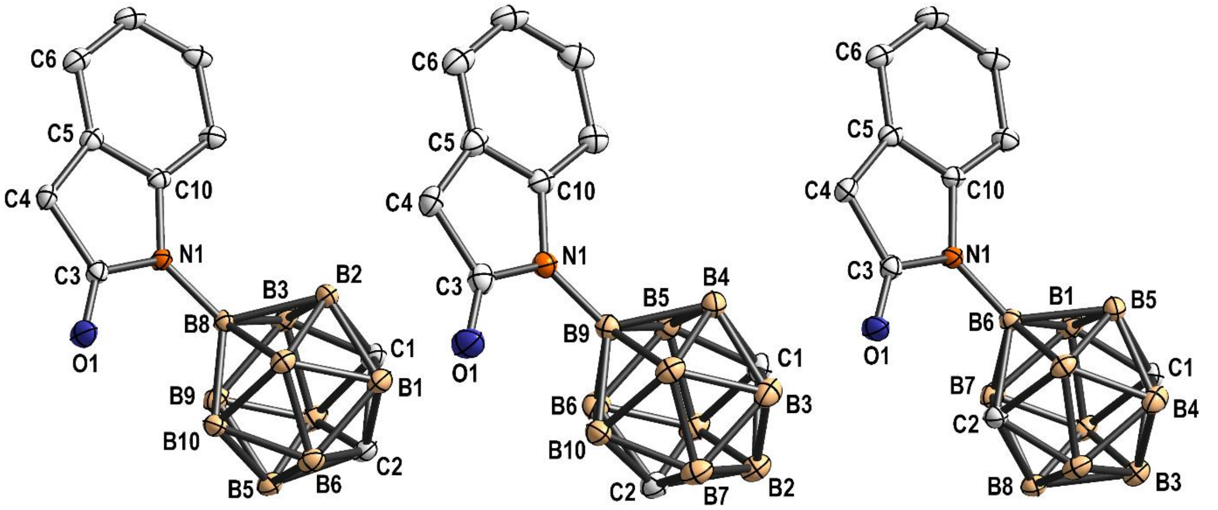

*Figure S 6. Molecular structure and labelling scheme of **o1**, **m1** and **p1**. Hydrogen atoms were omitted for clarity. Displacement ellipsoids are drawn at the 50% probability level.*

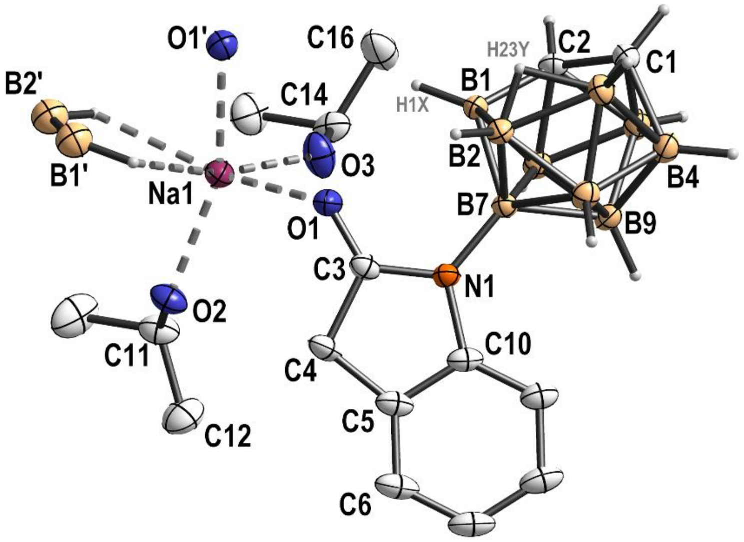

*Figure S 7. Molecular structure and labelling scheme of **2**. Hydrogen atoms - except carbaborane CH and BH - and disordered atoms were omitted for clarity. Displacement ellipsoids are drawn at the 50% probability level.*

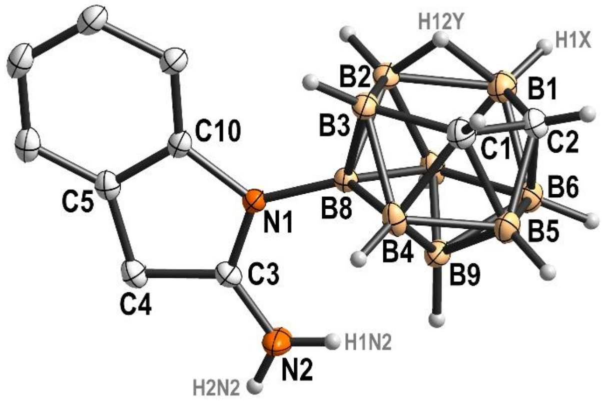

Figure S 8. Molecular structure and labelling scheme of ***3****. Hydrogen atoms - except NH and carbaborane CH and BH - and disordered atoms were omitted for clarity. Displacement ellipsoids are drawn at the 50% probability level.*

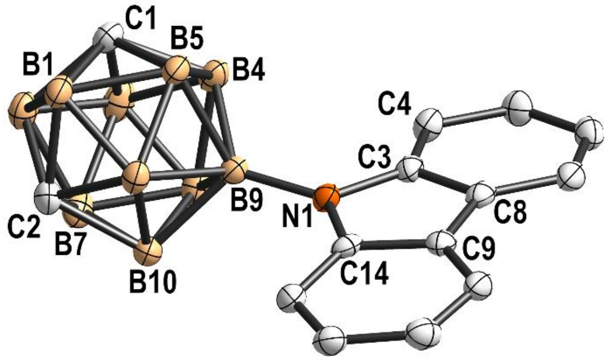

Figure S 9. Molecular structure and labelling scheme of ***8****. Hydrogen atoms were omitted for clarity. Displacement ellipsoids are drawn at the 50% probability level.*

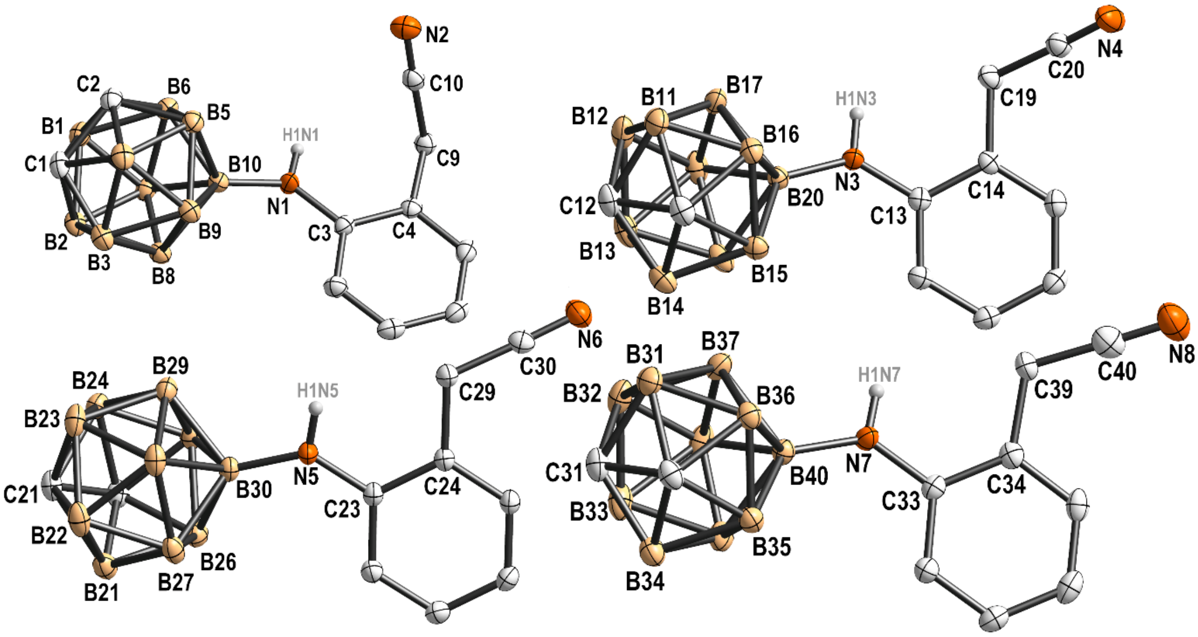

Figure S 10. Molecular structure and labelling scheme of ***9****. Four independent molecules crystallise in the asymmetric unit. Hydrogen atoms and one disordered nitrile substituent were omitted for clarity. Displacement ellipsoids are drawn at the 50% probability level.*

5 DFT Calculations

Density functional theory (DFT) calculations were carried out with ORCA4.[5] Geometries were optimized and confirmed as minima by frequency calculations (no imaginary frequencies) at the BLYP-D3BJ/DZVP-DFT level of theory.[6] Highest contribution from atomic orbitals to the LUMO were determined from Löwdin population analysis, as implemented in ORCA4. Images were rendered with UCSF Chimera.[7]

To further investigate this reactivity, we employed frontier molecular orbitals as a reactivity index for the attack of a nucleophile (water or hydroxide) on the amide carbon atom at position 2 (Figure S 11). The density of the lowest unoccupied molecular orbital (LUMO) usually correlates well with the reactivity towards a nucleophilic reagent.[8] The LUMO of the theoretical direct phenyl analogue *N*-phenyl oxindole **CPF** is a π* system extending on both the oxindole and phenyl moieties, with the highest contribution from the amide carbon p atomic orbital at position 2 (Figure S 11). This is in agreement with the known ring opening of oxindole upon reaction with a nucleophile. On the other hand, the LUMO of carboranyl species ***m*1** is mainly located on the oxindole moiety and the highest contribution comes from the p atomic orbital at the aromatic carbon atom at position 4 (Figure S 11). The shift in LUMO density upon changing the phenyl to *m*-carborane might explain why the ring opening of oxindole becomes less favored for ***m*1**. The highest contribution to the LUMO of the *para*-carboranyl derivative ***p*1** again comes from the amide carbon p atomic orbital at position 2 (Figure S 11). However, the stabilizing CH···O intramolecular interaction might hinder the ring opening of oxindole in ***p*1**. Furthermore, the rotation of the *p*-carborane cluster has a strong influence on the LUMO of ***p*1**. Thus, considering a rotamer with higher energy (ΔG = 14.12 kJ mol^{-1}) and without a CH···O interaction, the highest contribution to the LUMO again in accordance with ***m*1** is located at the carbocyclic carbon at position 4 (Figure 4, bottom right).

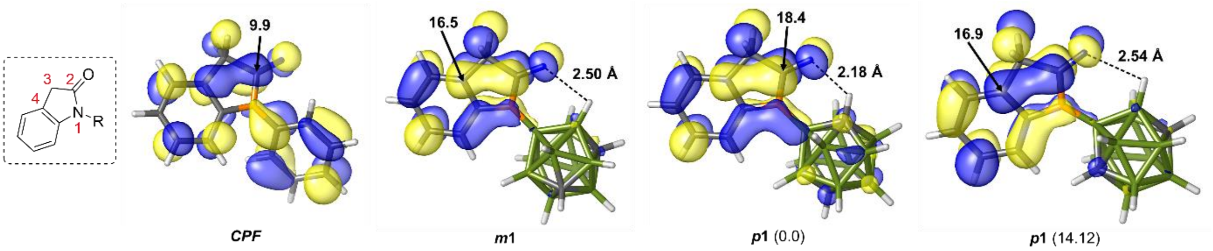

*Figure S 11. BLYP-D3BJ/DZVP-DFT gas phase optimized geometries, lowest unoccupied molecular orbitals of **CPF**, **m1** and **p1** (relative energy of rotamers in kJ mol^{-1}) with selected intramolecular Y-H···O (Y = C, B) interactions. The location and magnitude of the highest contribution from atomic orbitals to the LUMO are shown along with selected distances. H: white, B: green, C: grey, N: orange, O: blue.*

[5] Neese, F. *WIREs Comput. Mol. Sci.* **2012**, *2* (1), 73–78.

[6] a) Lee, C. T.; Yang, W. T.; Parr, R. G., Phys. Rev. B: Condens. Matter Mater. Phys. 1988, 37, 785−789. b) Hostaš, J.; Řezáč, J., *J. Chem. Theory Comput.* **2017**, *13*, 3575–3585.

[7] Pettersen, E. F.; Goddard, T. D.; Huang, C. C.; Couch, G. S.; Greenblatt, D. M.; Meng, E. C.; Ferrin, T. E.; *J. Comput. Chem.* **2004**, *25*, 1605–1612.

[8] Parr, Robert G.; Yang, Weitao, J. Am. Chem. Soc. **1984**, 106, 4049–4050.

6 Molecular docking

The molecular docking was performed with the AutoDockTools4 software[9] using the Lamarckian Genetic Algorithm[10]. The partial charges for structures were obtained using HF-3c implemented in ORCA[11]. The water molecules were eliminated, and the non-polar hydrogen atoms were merged. The docking area was limited by the constructed grid box of the size 40 x 40 x 40 centered at 109.572, 51.688, 67.892 of x,y,z-coordinates (based on the position of the carborane derivative reported in the crystal structure 4Z0L[12]). The following parameters were used in the docking: number of hybrid GA-LS runs: 100; population size: 150; maximum number of energy evaluations: 2,500,000, maximum number of top individuals to survive to next generation: 1; rate of gene mutation: 0.02; rate of crossover: 0.8; Mean of Cauchy distribution for gene mutation: 0.0; variance of Cauchy distribution for gene mutation: 1.0.

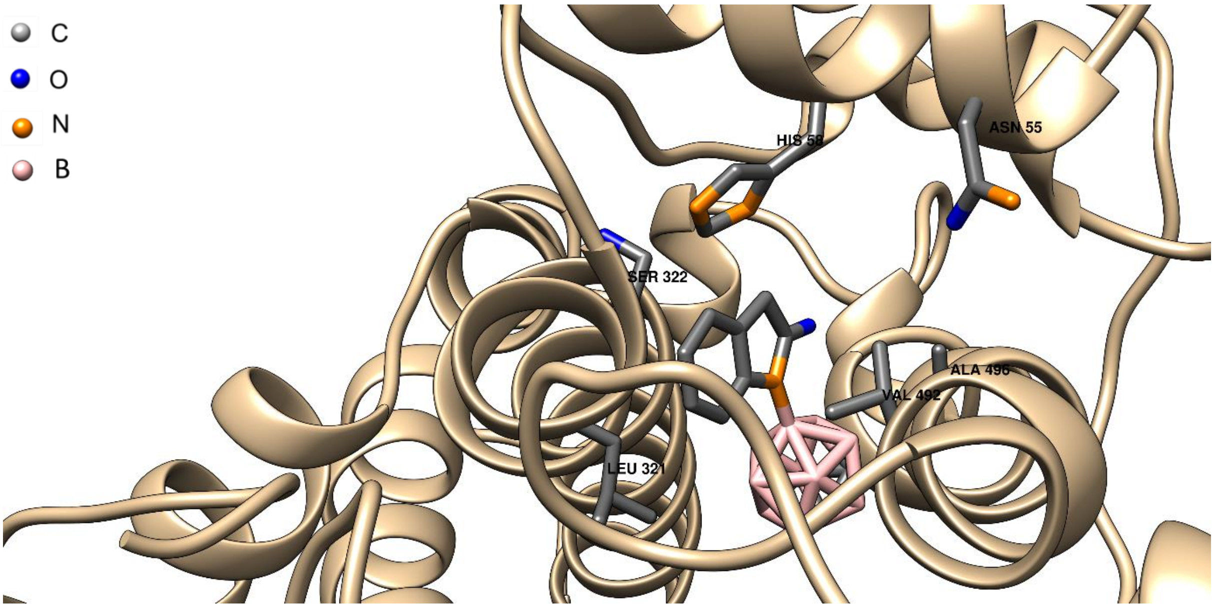

Figure S 12. ***o1**, ΔG = −8.20 kcal mol^{-1}.*

[9] Morris, G. M.; Huey, R.; Lindstrom, W.; Sanner, M. F.; Belew, R. K.; Goodsell, D. S.; Olson, A. J.,*J. Comput. Chem.* **2009**, *30* (16), 2785–2791.

[10] Morris, G. M.; Goodsell, D. S.; Halliday, R. S.; Huey, R.; Hart, W. E.; Belew, R. K.; Olson, A. J., *J. Comput. Chem.* **1998**, *19* (14), 1639–1662.

[11] Neese, F. The ORCA Program System. *WIREs Comput. Mol. Sci.* **2012**, *2* (1), 73–78..

[12] Neumann, W.; Xu, S.; Sárosi, M. B.; Scholz, M. S.; Crews, B. C.; Ghebreselasie, K.; Banerjee, S.; Marnett, L. J.; Hey-Hawkins, E., *ChemMedChem* **2016**, *11* (2), 175–178.

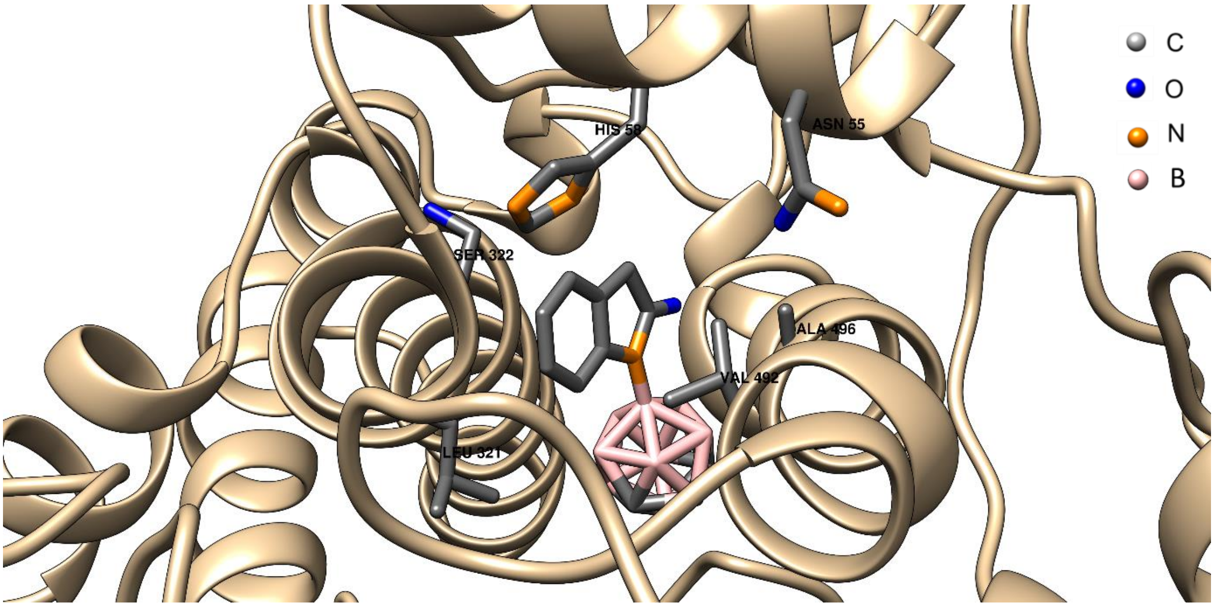

Figure S 13. ***m1****, ΔG = −7.98 kcal mol*$^{-1}$*.*

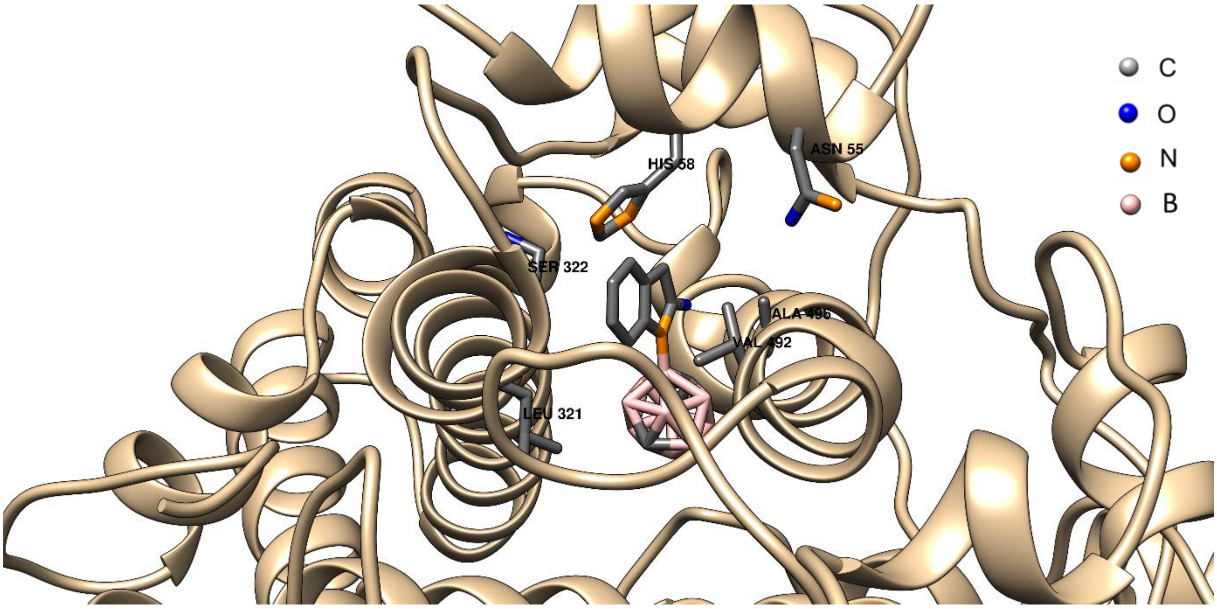

Figure S 14. ***p1****, ΔG = −5.92 kcal mol*$^{-1}$*.*

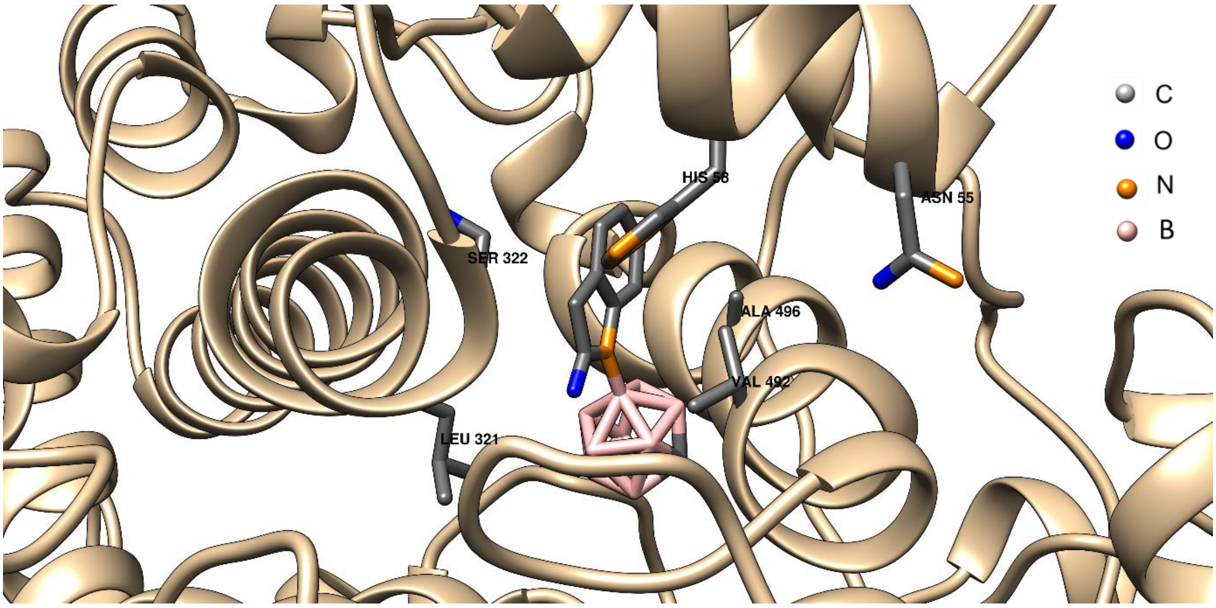

*Figure S 15. **2**, ΔG = −8.14 kcal mol*$^{-1.}$

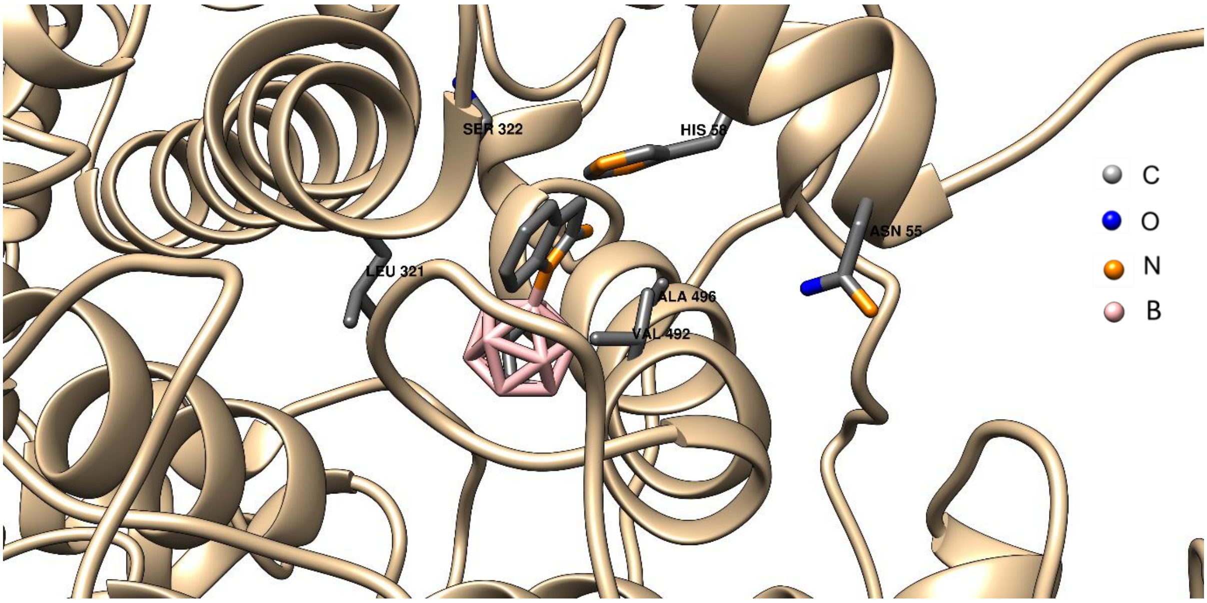

*Figure S 16. **3**, ΔG = −8.86 kcal mol*$^{-1}$*.*

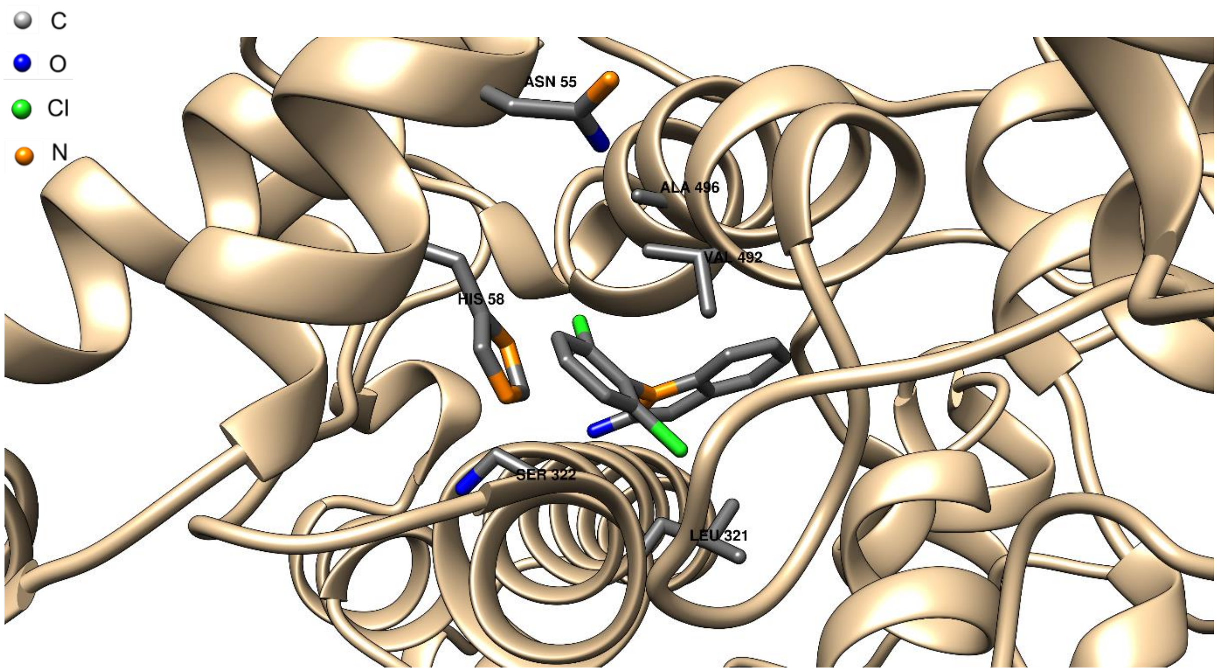

Figure S 17. ***CCF****, ΔG = -7.54 kcal mol*$^{-1}$*.*

7 NMR Data for Compounds 6, 7, *o*1, *m*1, *p*1, 2, 3, and 9

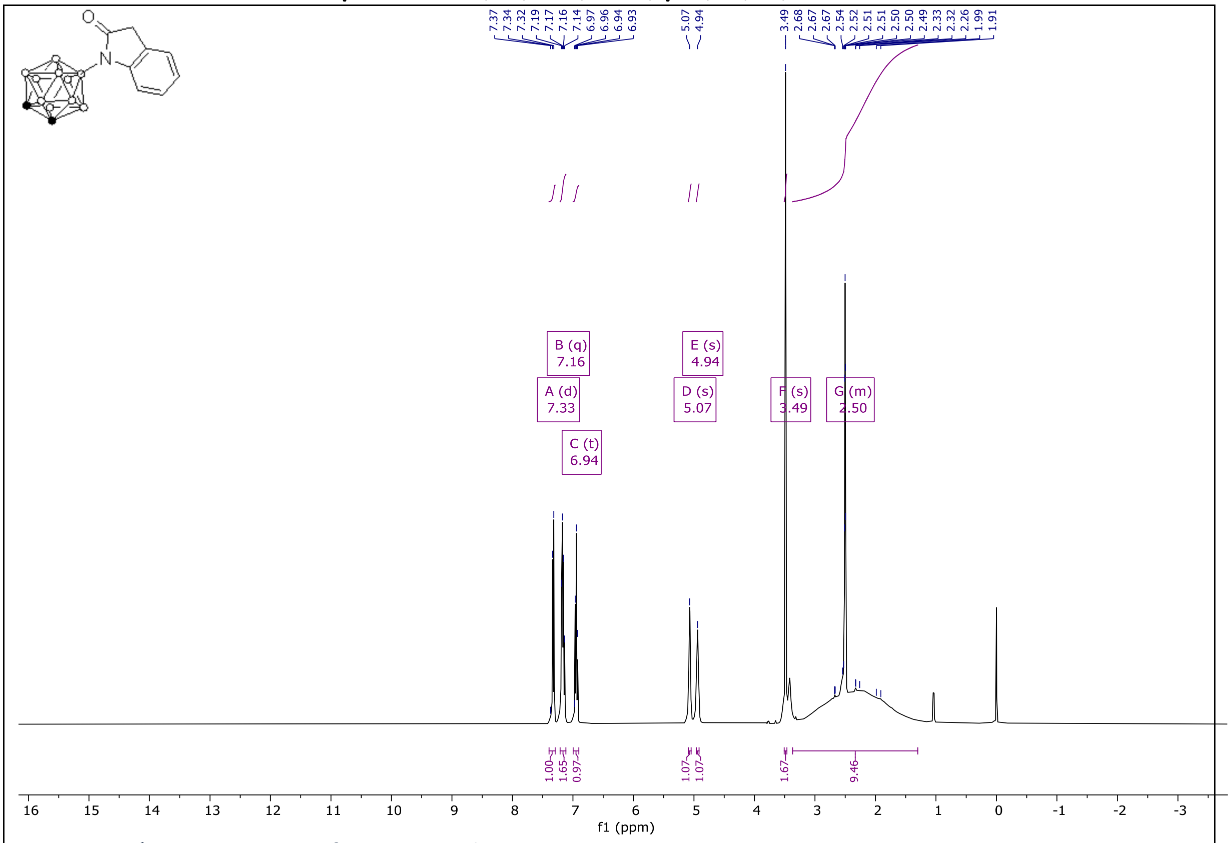

*Figure S 18. ^{1}H NMR spectrum of **o1** in DMSO-d_6.*

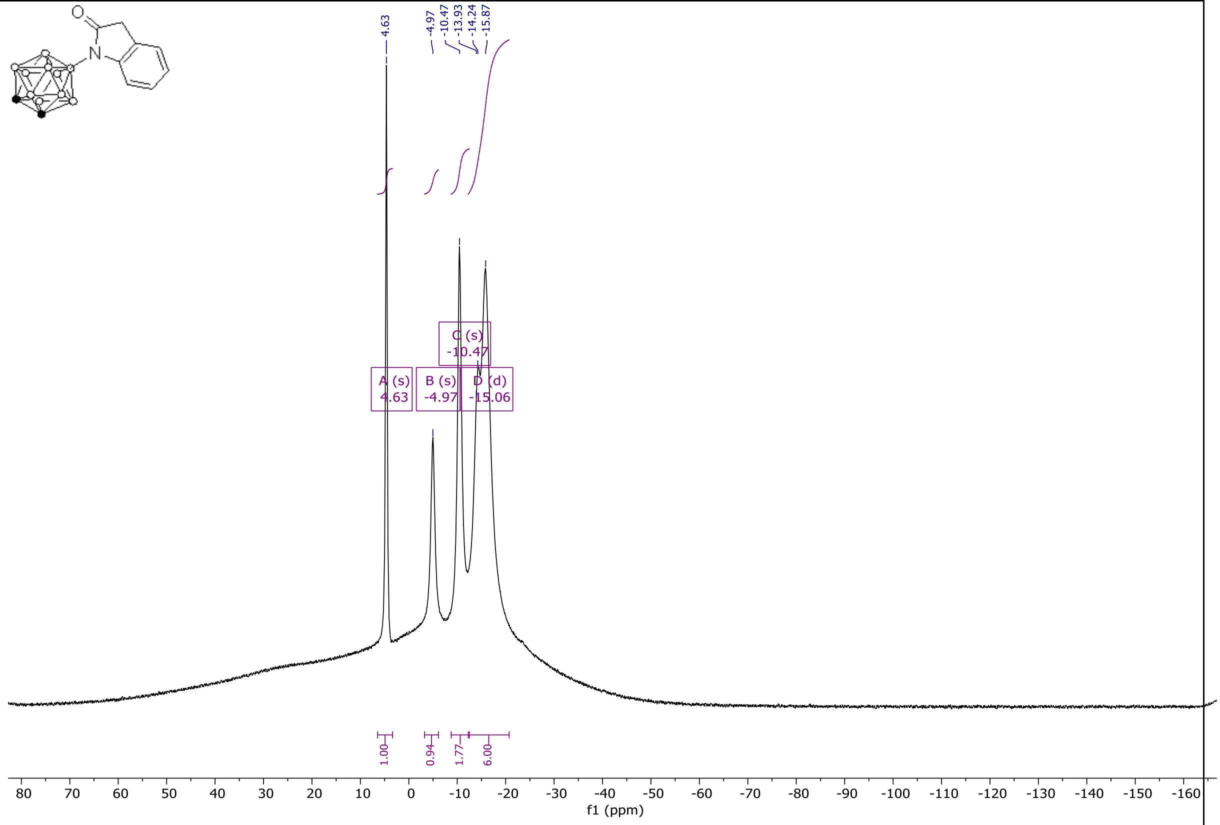

*Figure S 19. ^{11}B{^{1}H} NMR spectrum of **o1** in DMSO-d_6.*

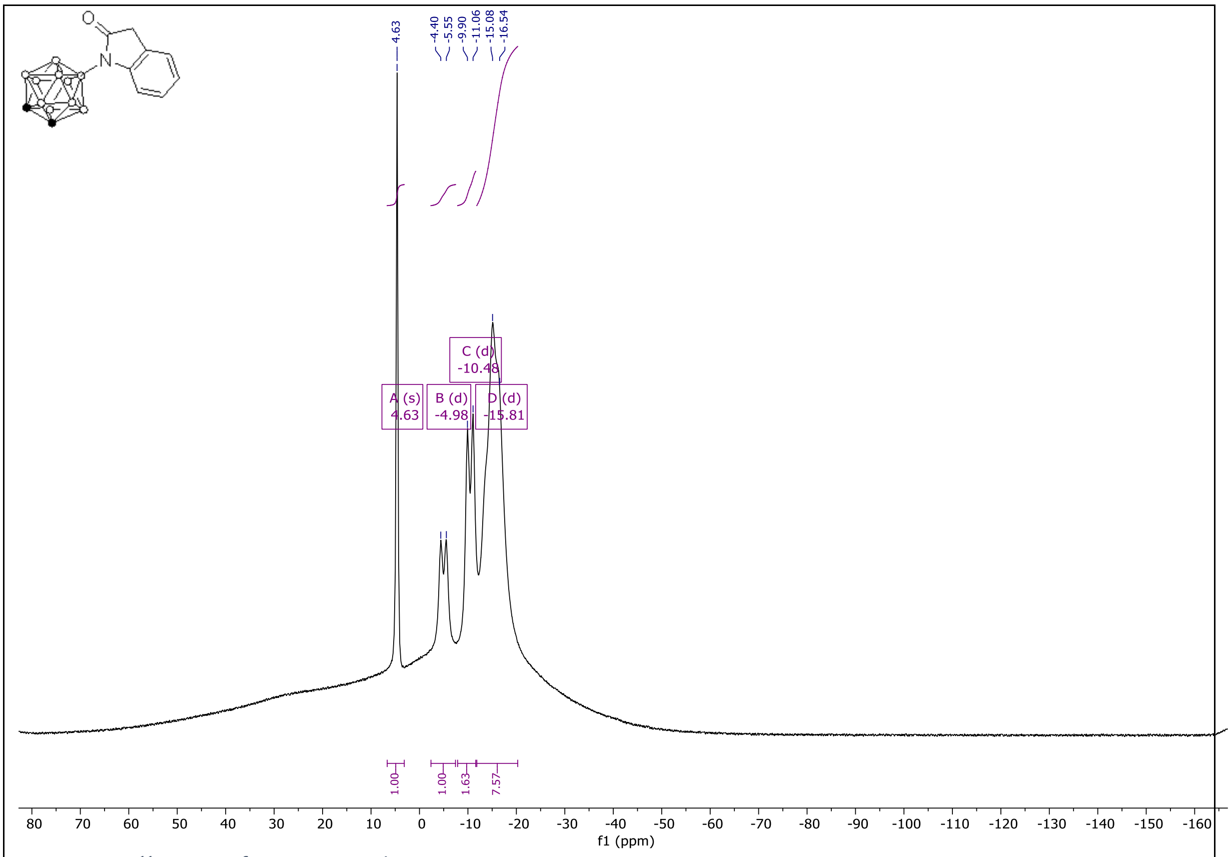

*Figure S 20. ^{11}B NMR of **o1** in DMSO-d$_6$.*

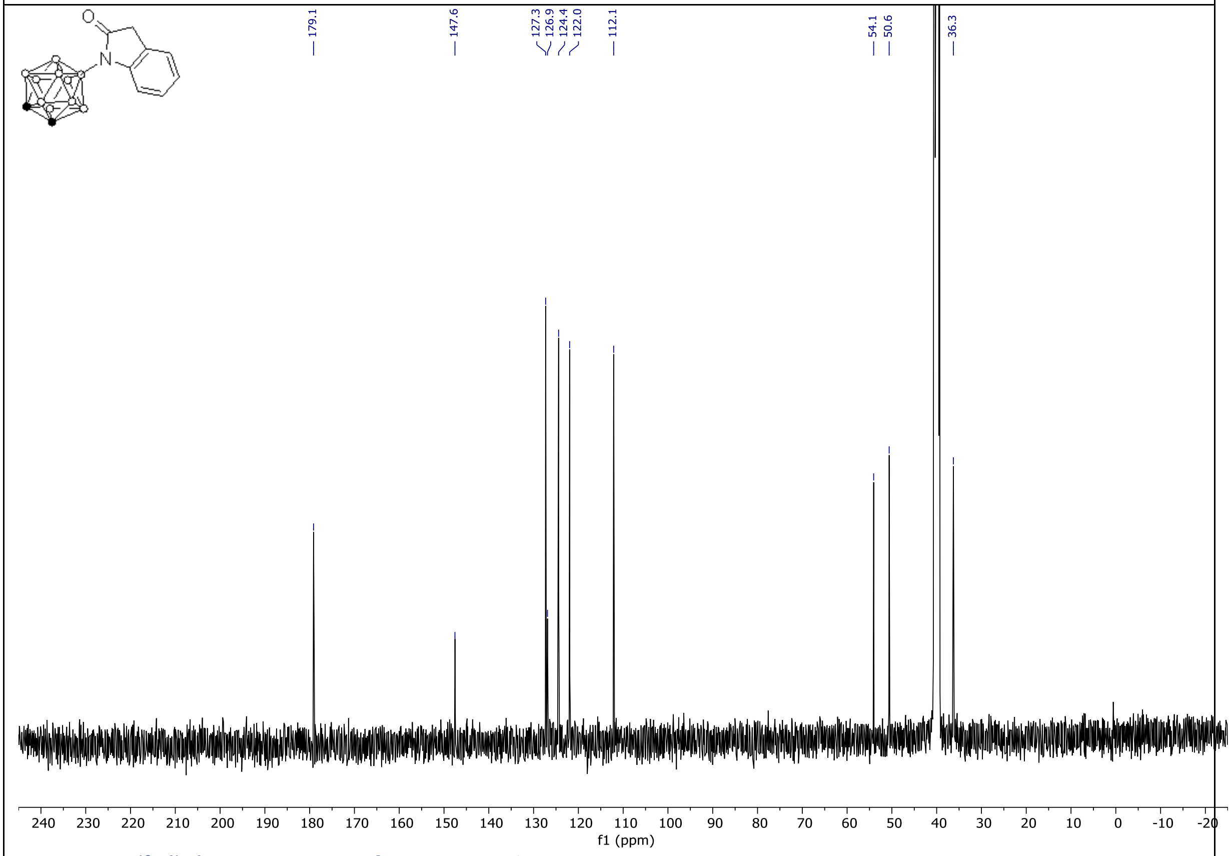

*Figure S 21. ^{13}C{^{1}H} NMR spectrum of **o1** in DMSO-d$_6$.*

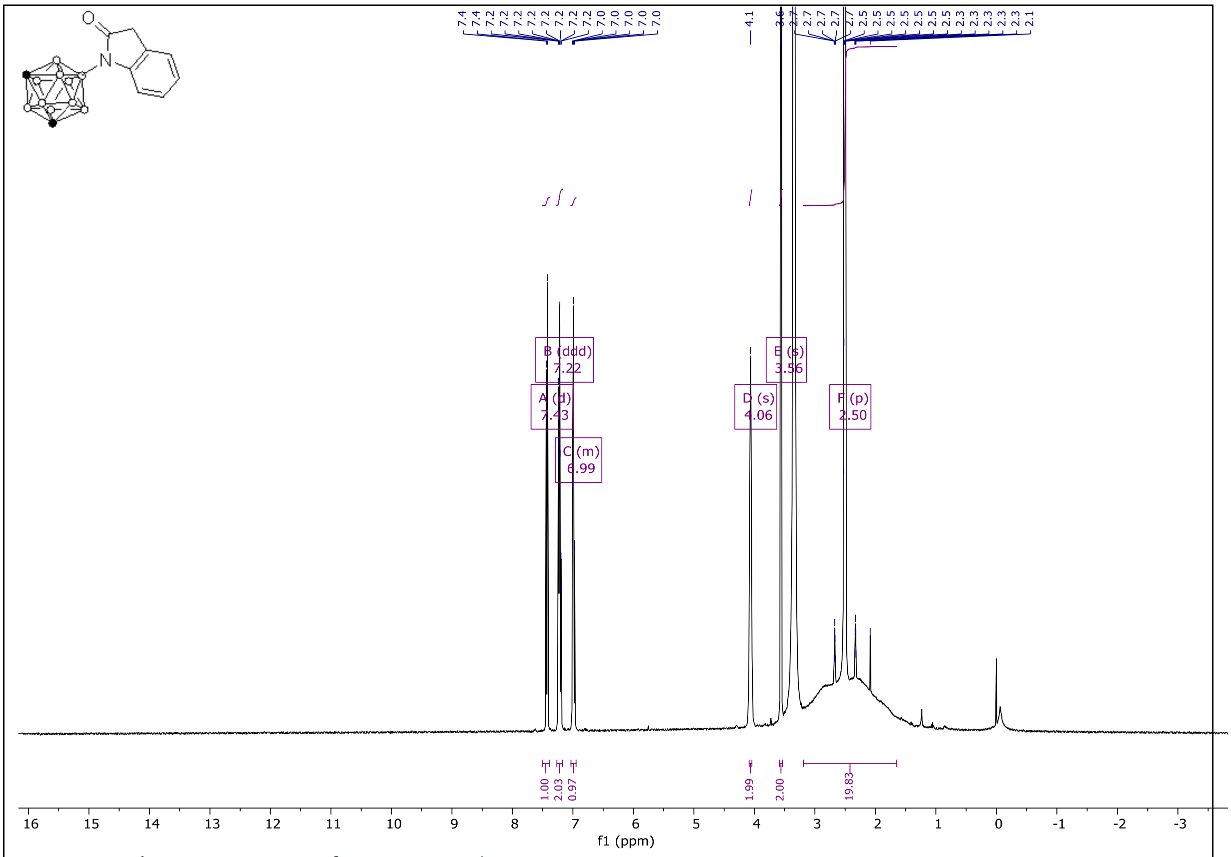

Figure S 22. ^{1}*H NMR spectrum of* ***m1*** *in DMSO-d*$_{6}$.

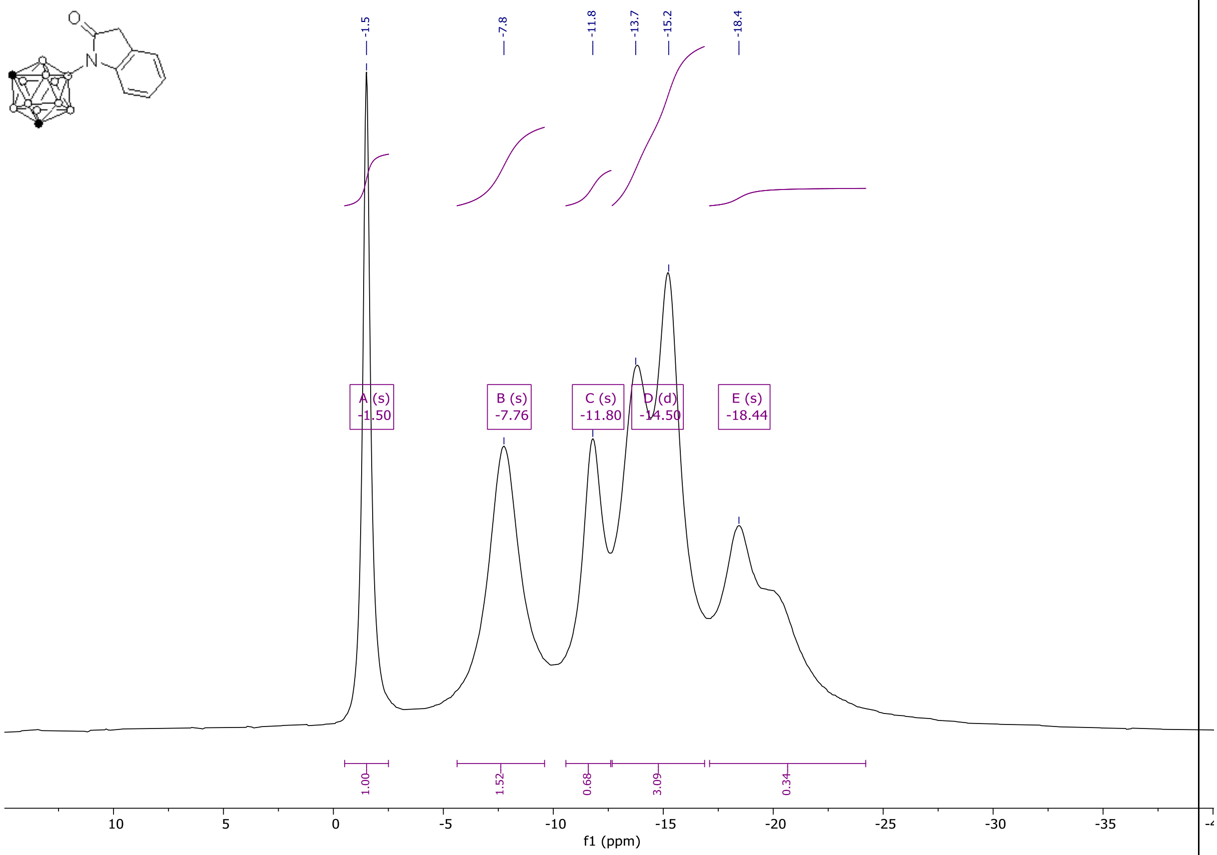

Figure S 23. ^{11}*B{*^{1}*H} NMR spectrum of* ***m1*** *in DMSO-d*$_{6}$.

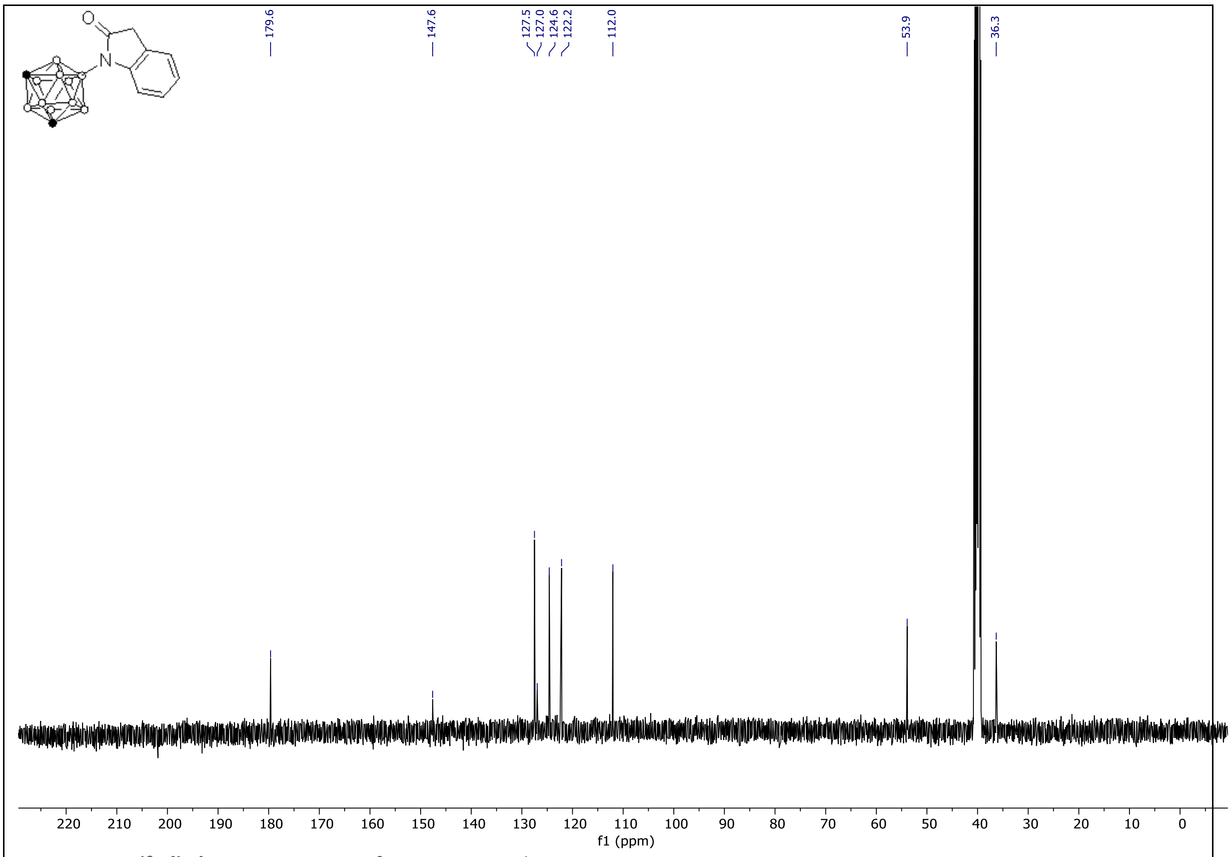

*Figure S 24. $^{13}C\{^{1}H\}$ NMR spectrum of **m1** in DMSO-d_6.*

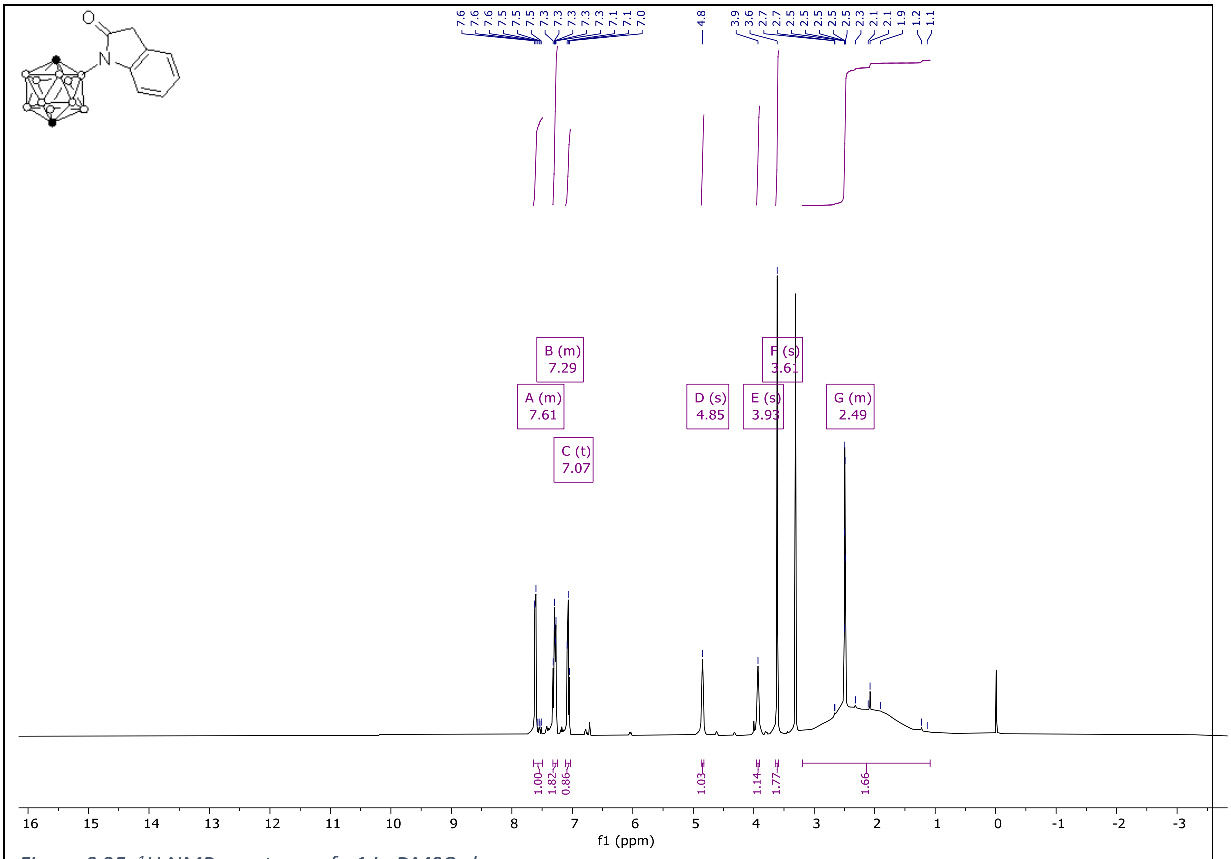

*Figure S 25. ^{1}H NMR spectrum of **p1** in DMSO-d_6.*

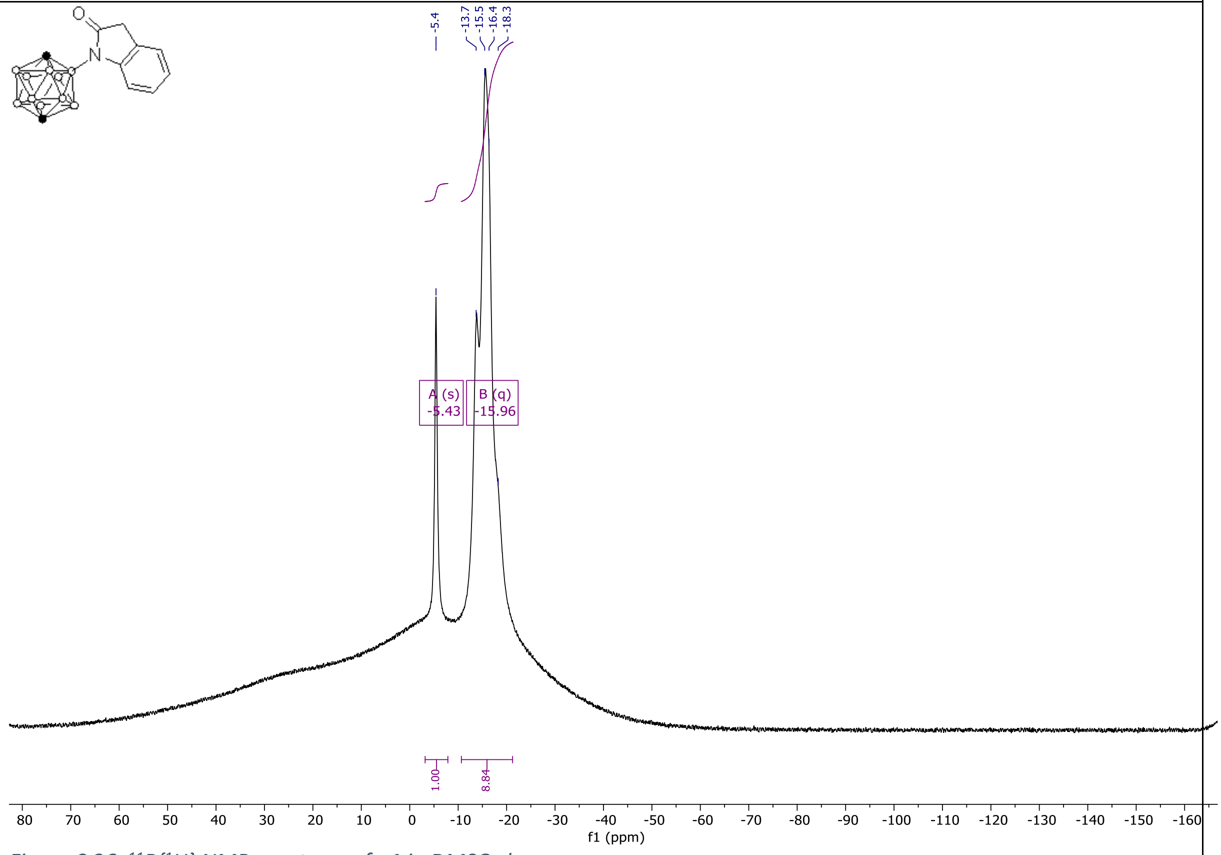

*Figure S 26. $^{11}B\{^{1}H\}$ NMR spectrum of **p1** in DMSO-d_6.*

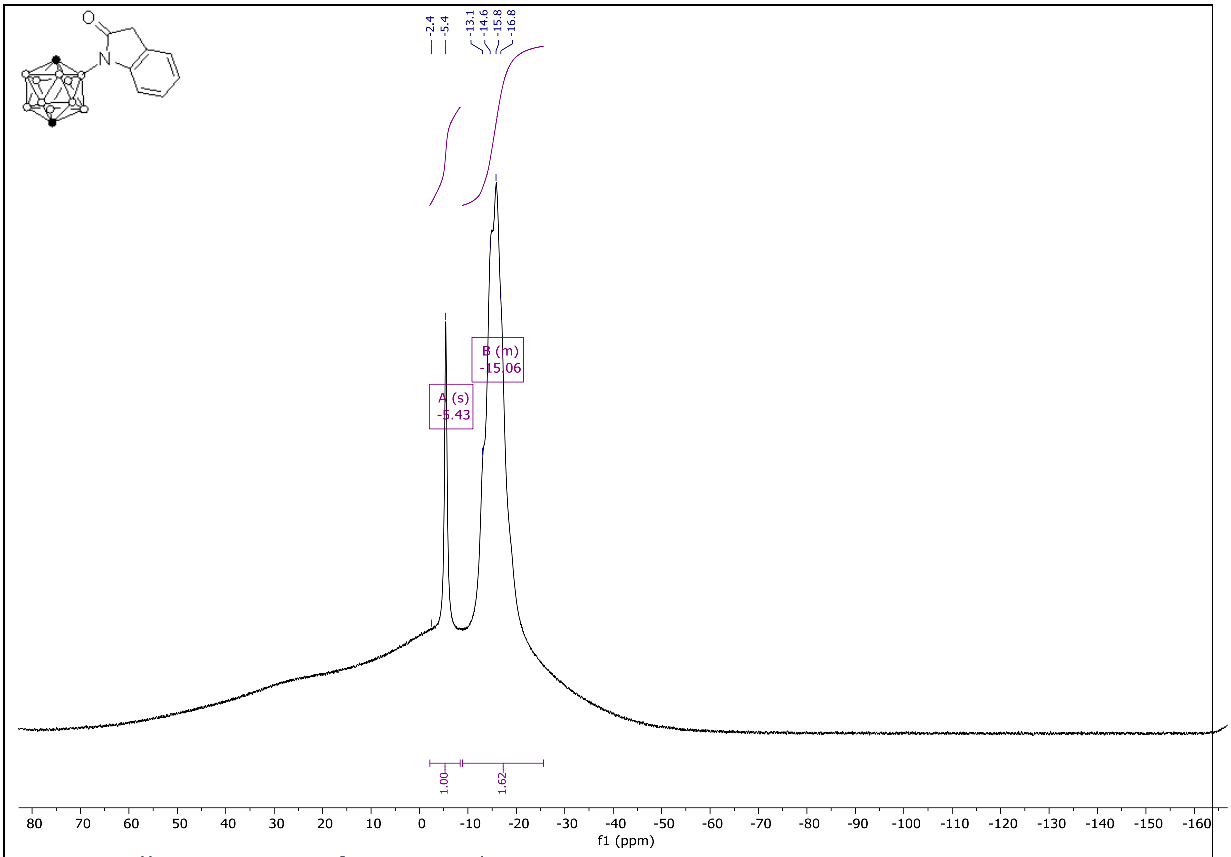

Figure S 27. ^{11}B *NMR spectrum of* ***p1*** *in DMSO-*d_6*.*

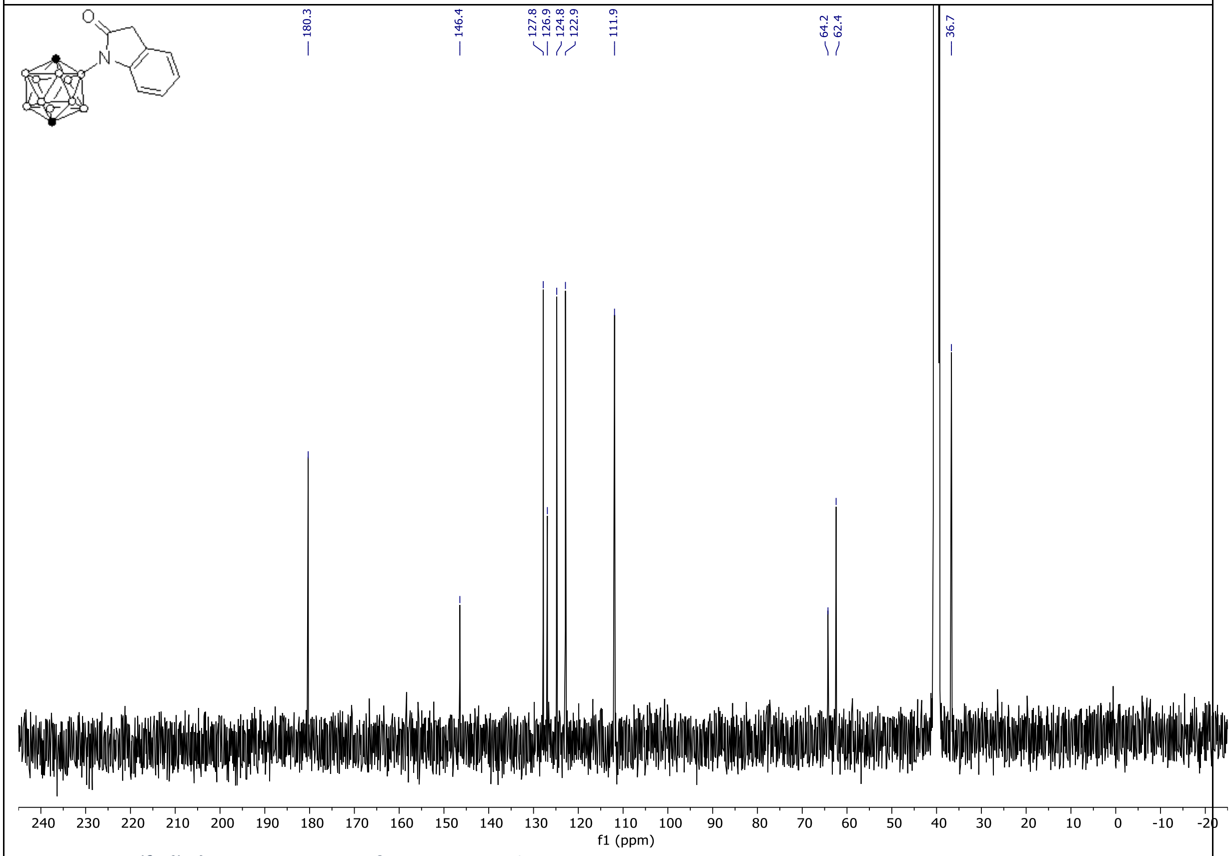

Figure S 28. $^{13}C\{^{1}H\}$ *NMR spectrum of* ***p1*** *in DMSO-*d_6*.*

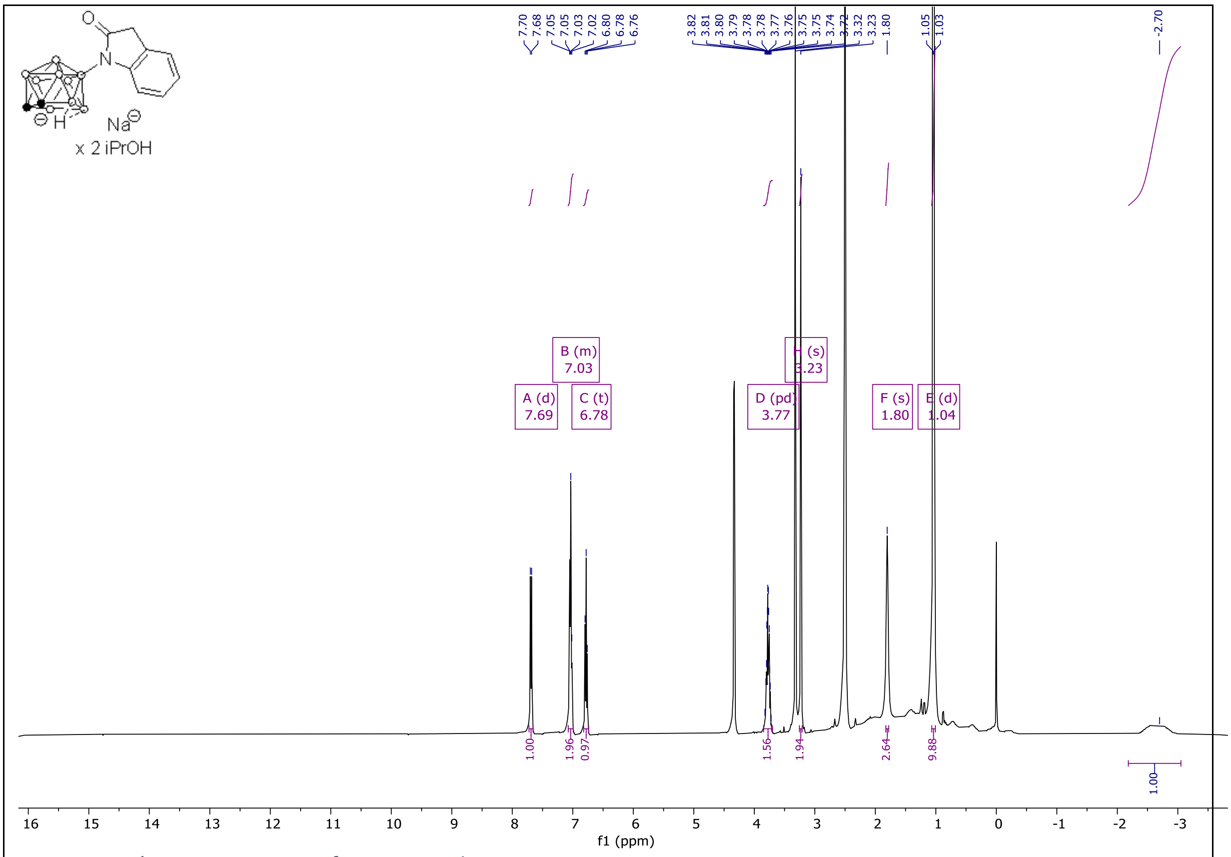

Figure S 29. ^{1}H NMR spectrum of **2** in DMSO-d_6.

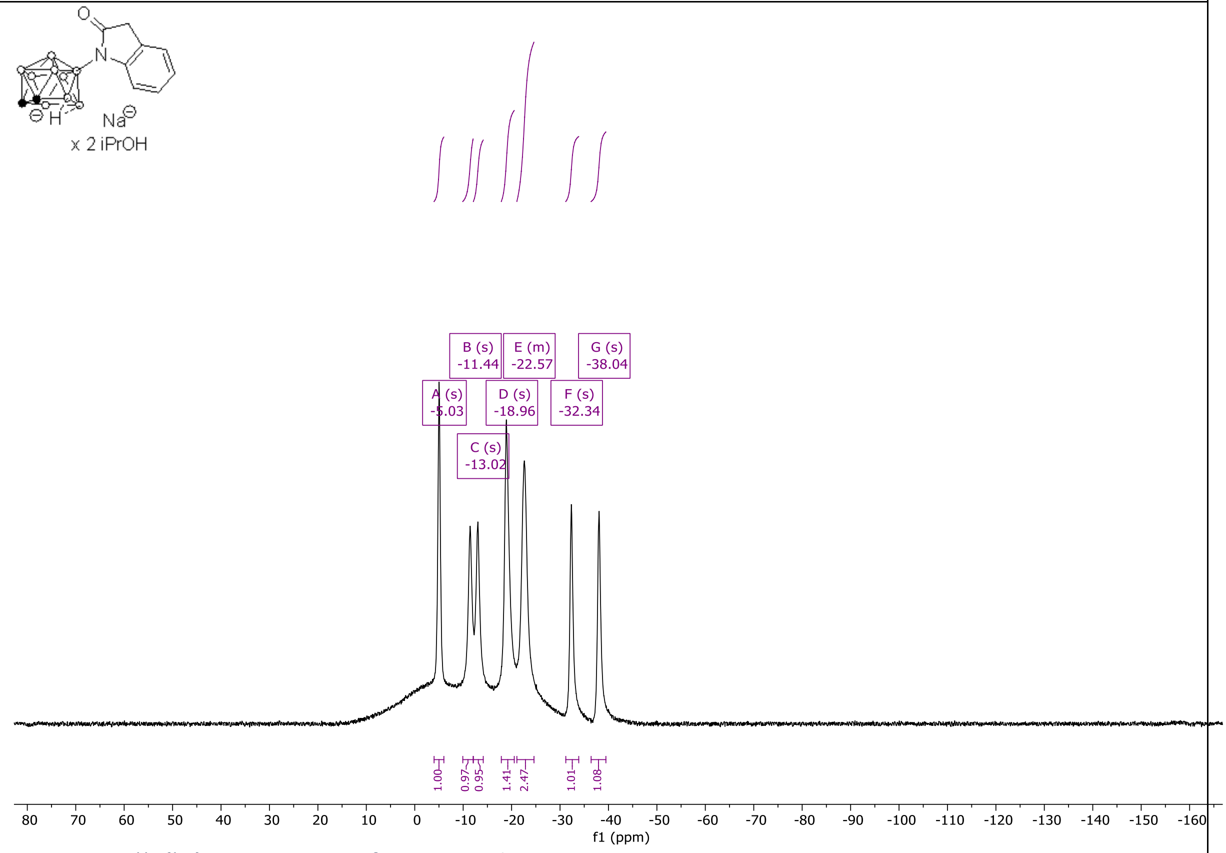

Figure S 30. ^{11}B{^{1}H} NMR spectrum of **2** in DMSO-d_6.

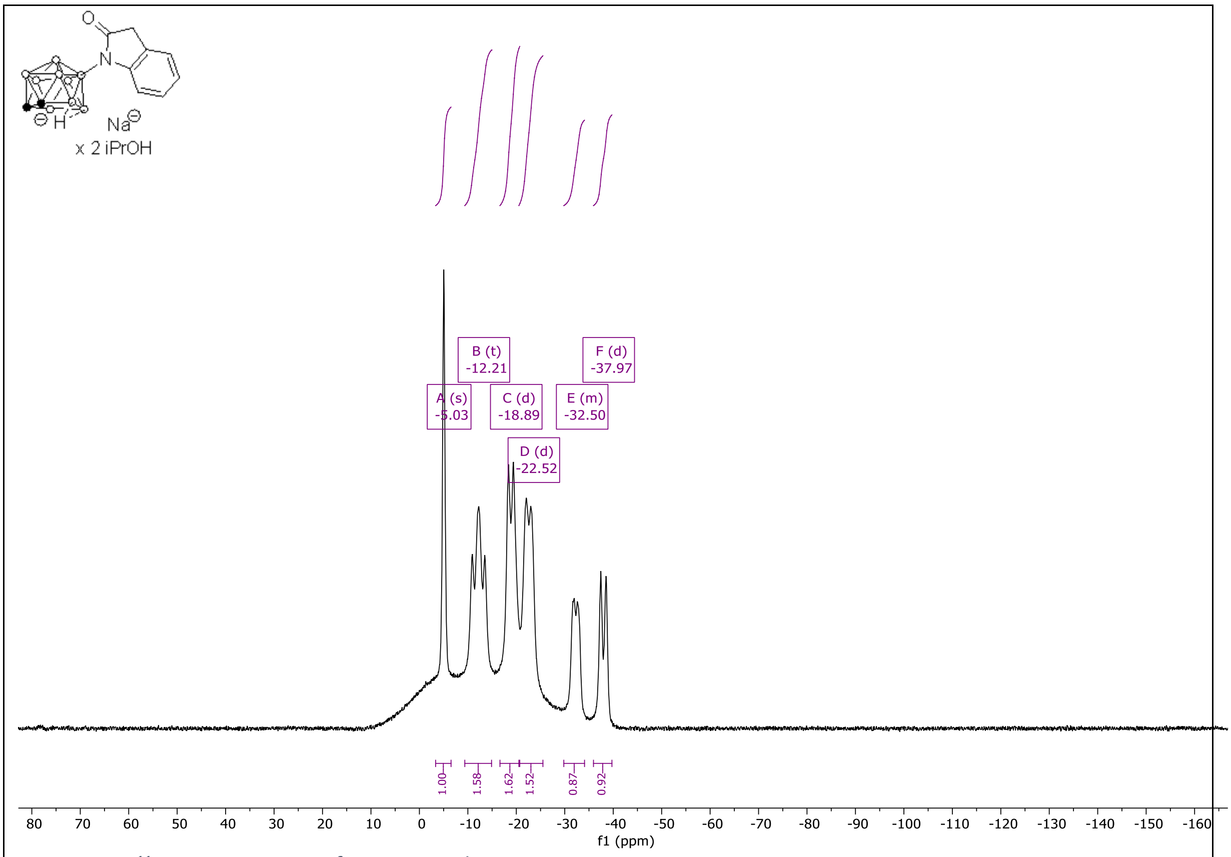

*Figure S 31. ^{11}B NMR spectrum of **2** in DMSO-d$_6$.*

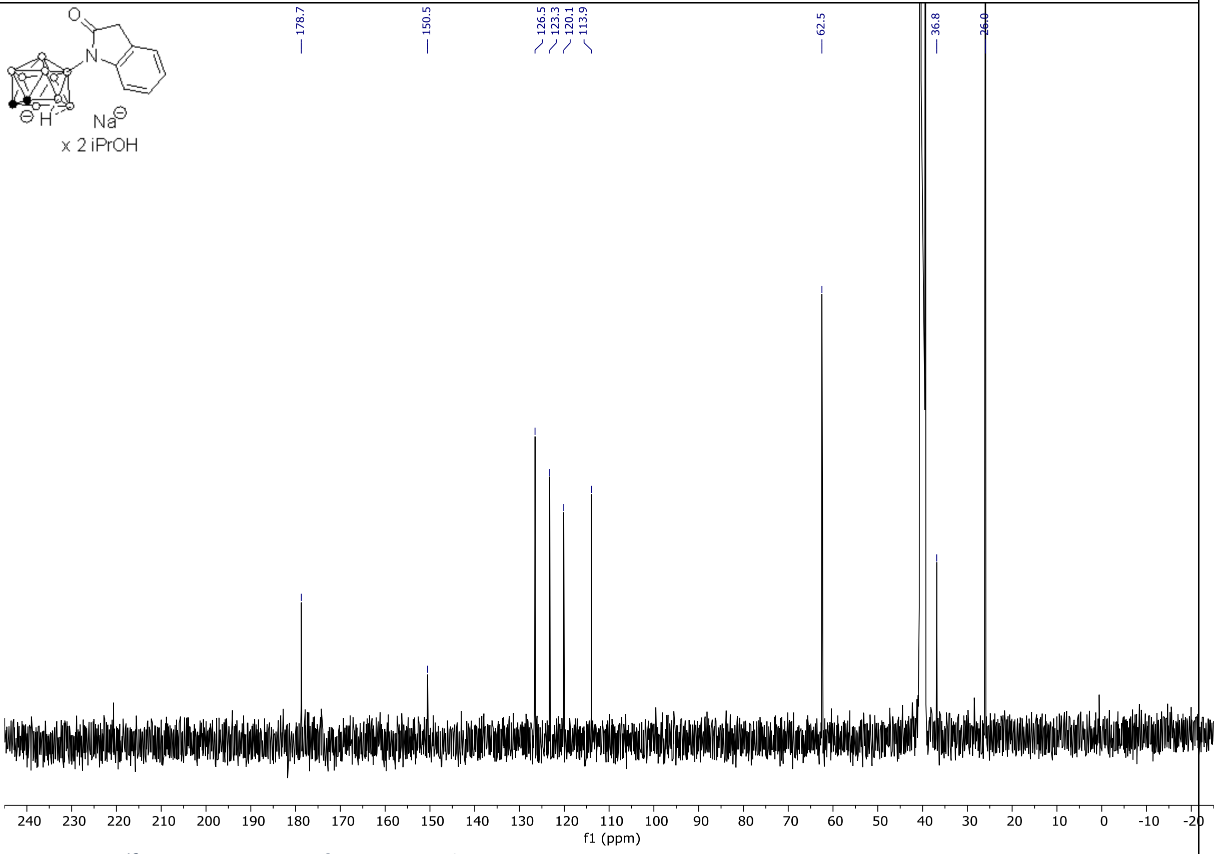

*Figure S 32. ^{13}C NMR spectrum of **2** in DMSO-d$_6$.*

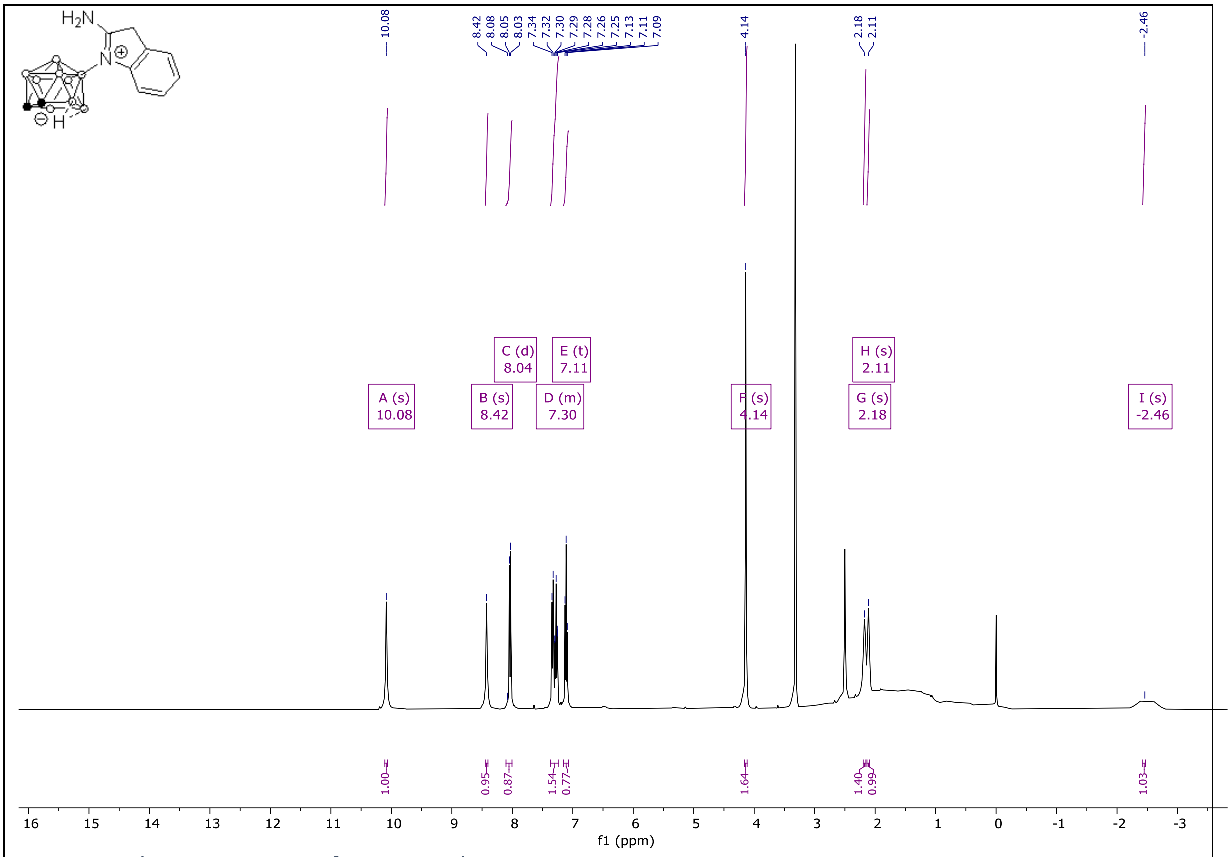

Figure S 33. ^{1}H NMR spectrum of ***3*** *in DMSO-d_6.*

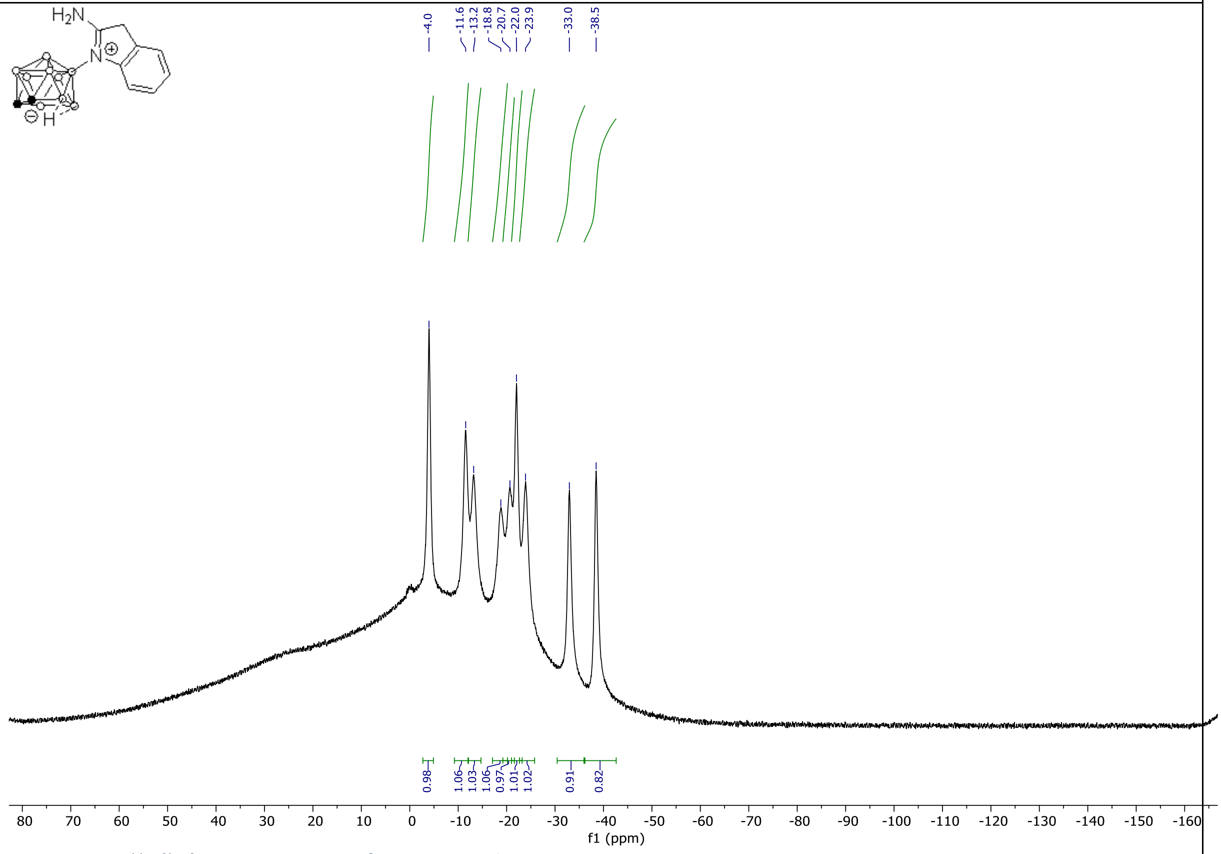

Figure S 34. ^{11}B{^{1}H} NMR spectrum of ***3*** *in DMSO-d_6.*

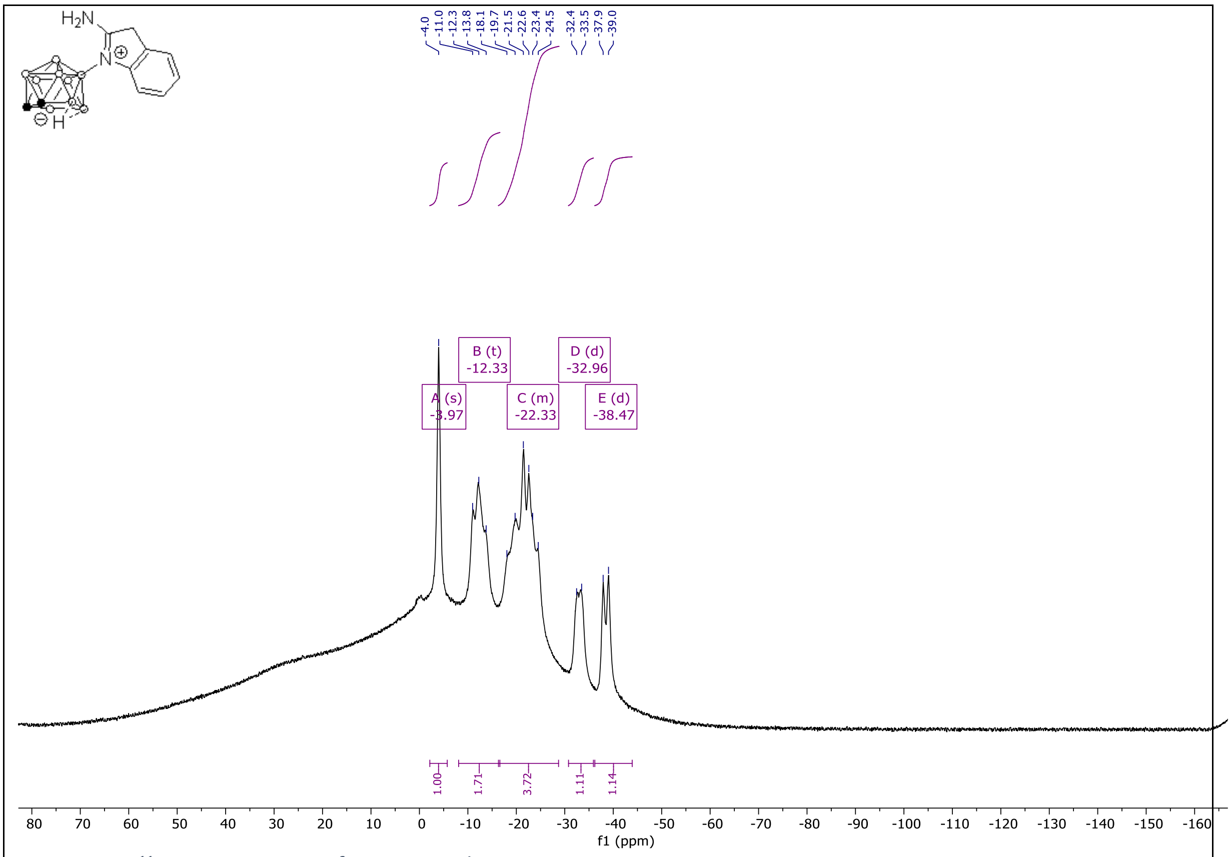

*Figure S 35. ^{11}B NMR spectrum of **3** in DMSO-d$_6$.*

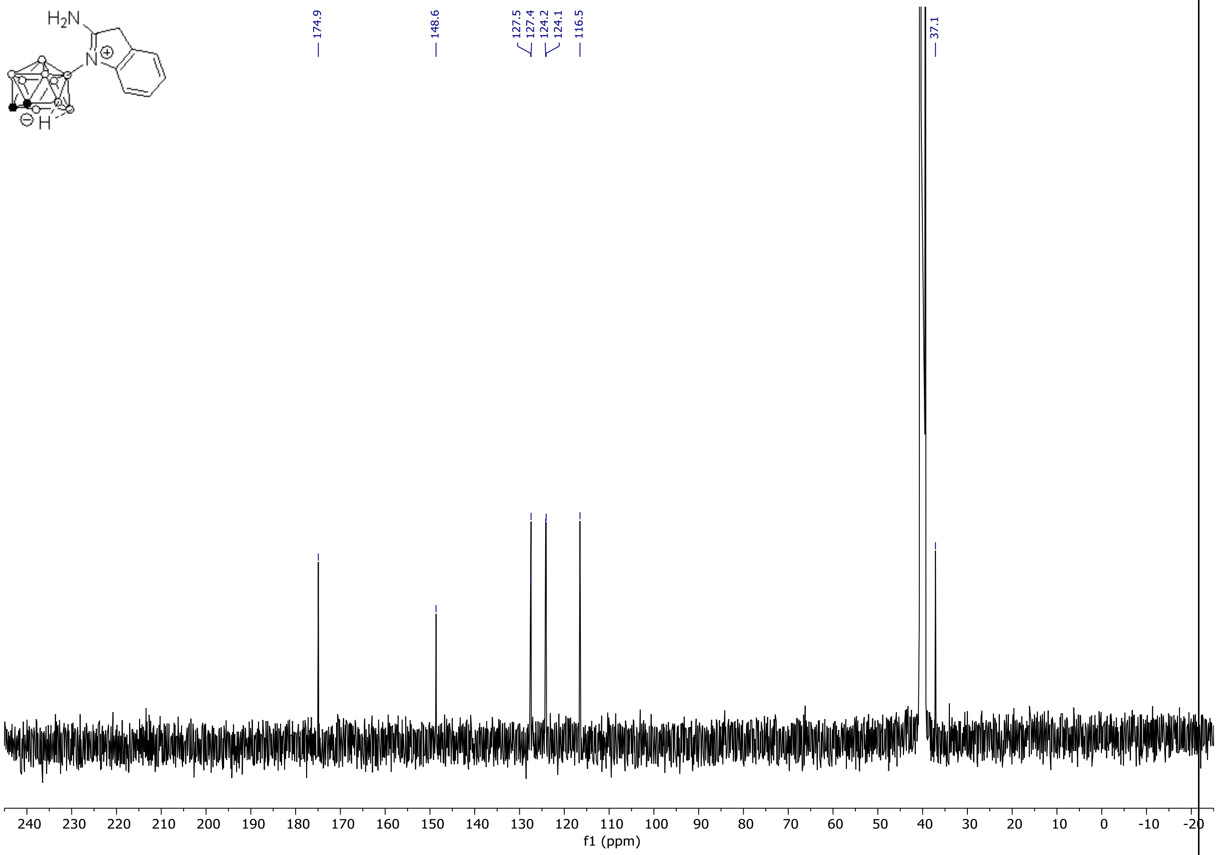

*Figure S 36. ^{13}C NMR spectrum of **3** in DMSO-d$_6$.*

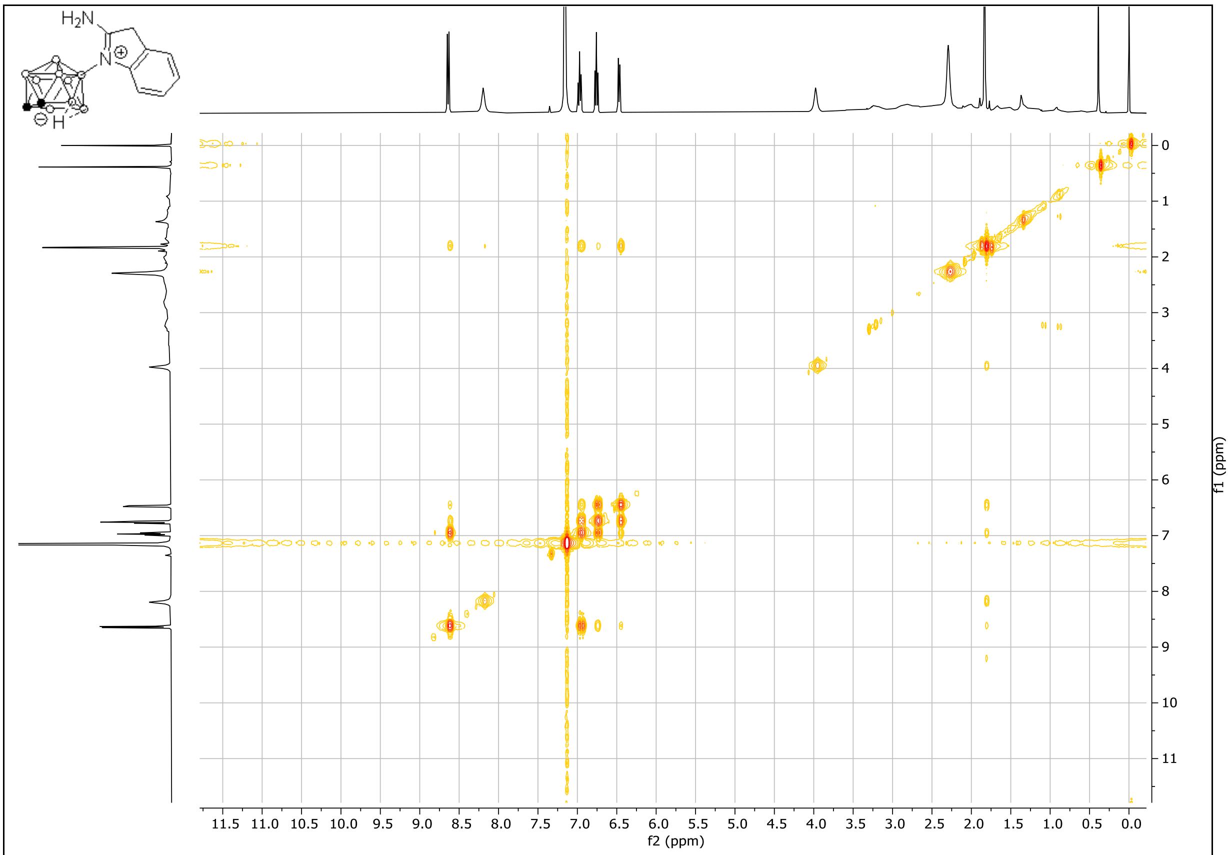

*Figure S 37. ^{1}H-^{1}H-COSY of **3** in C_6D_6.*

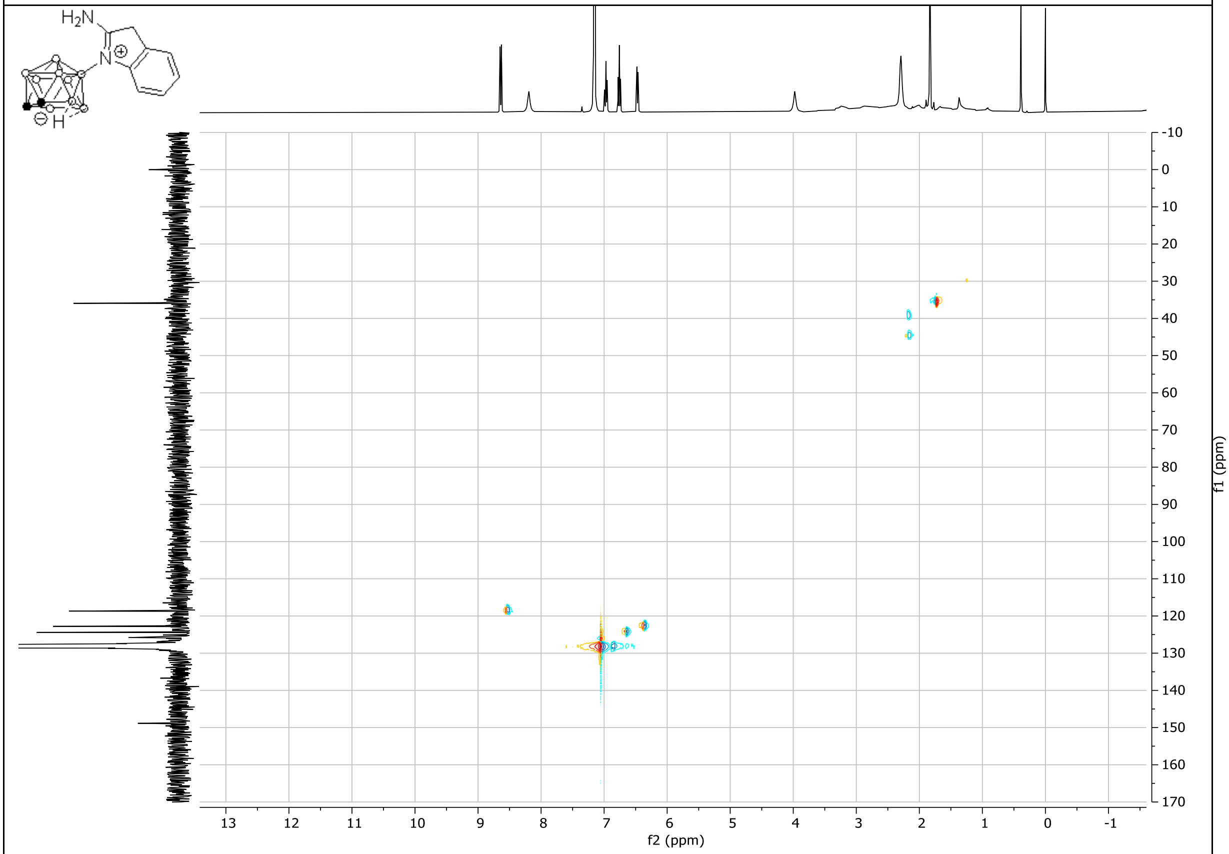

*Figure S 38. HSQC of **3** in C_6D_6.*

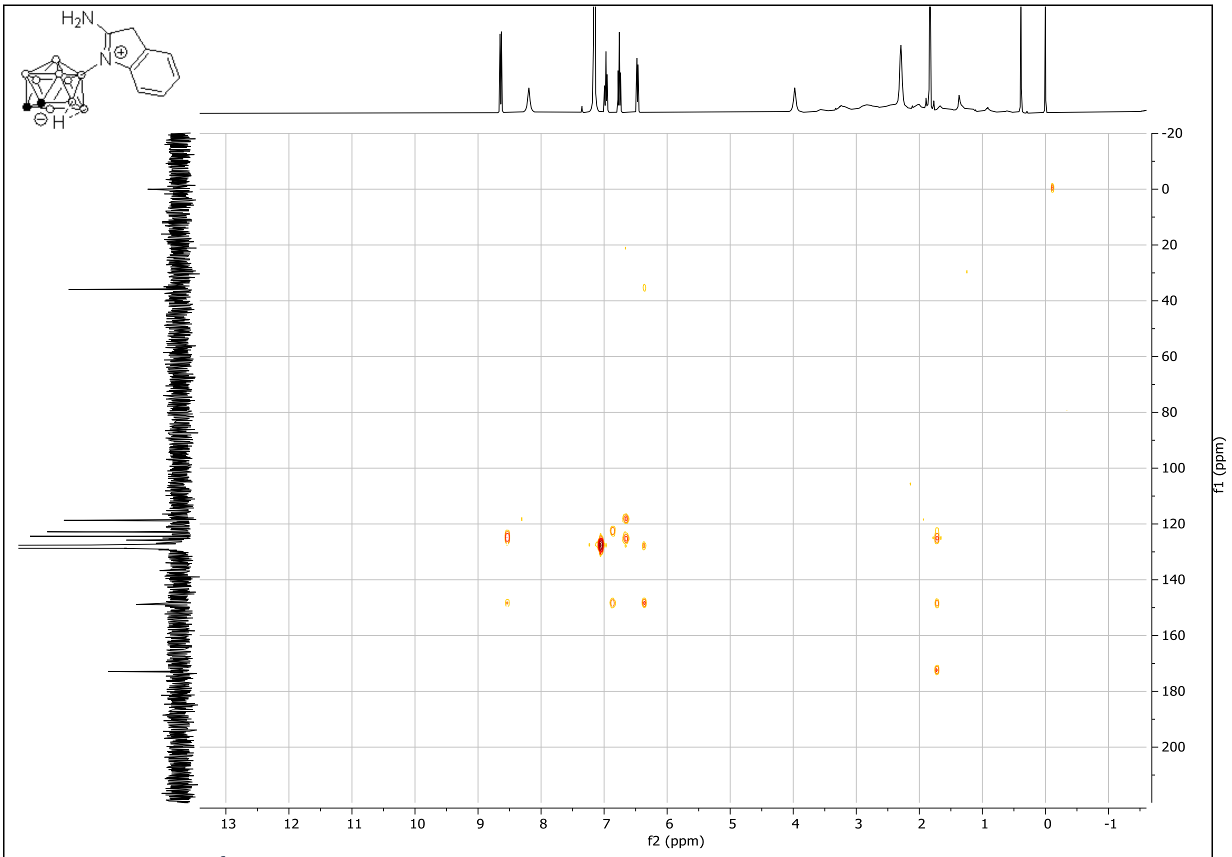

*Figure S 39. HMBC of **3** in C_6D_6.*

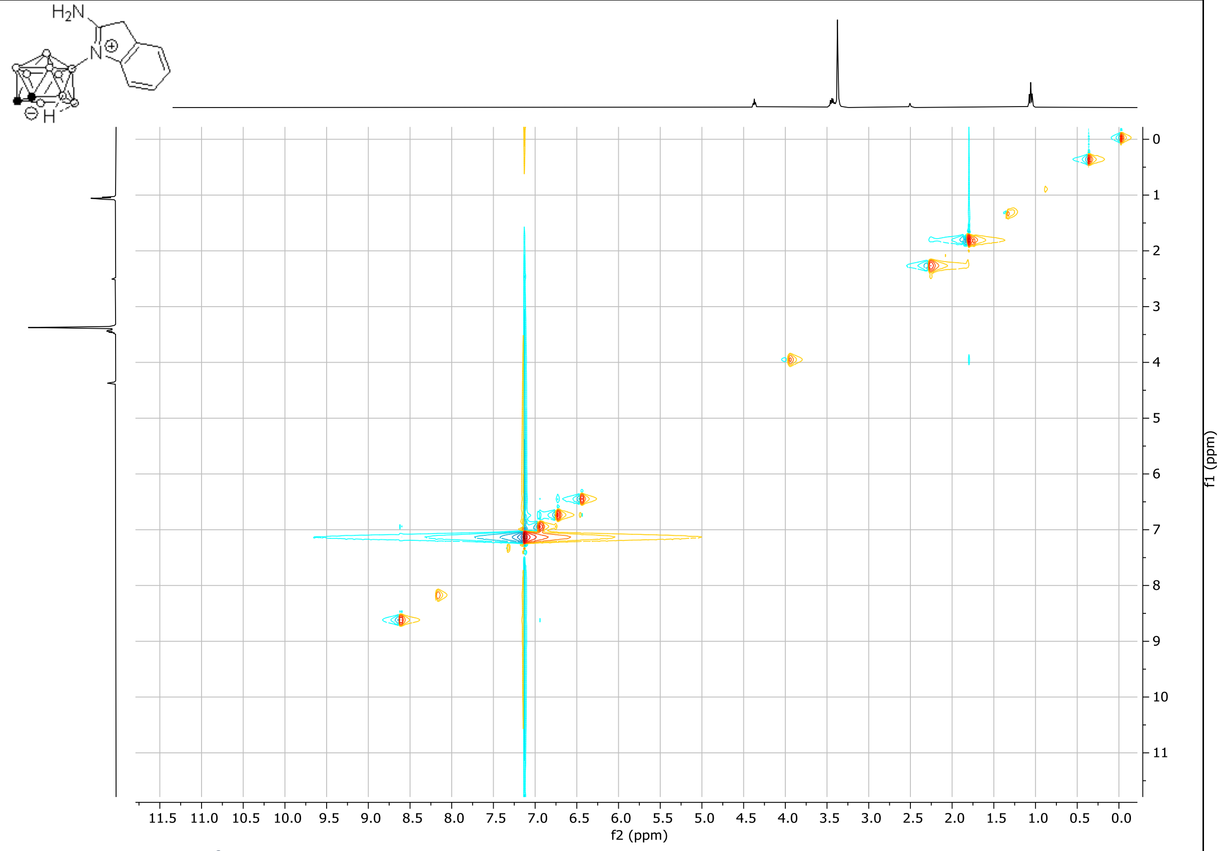

*Figure S 40. NOESY of **3** in C_6D_6.*

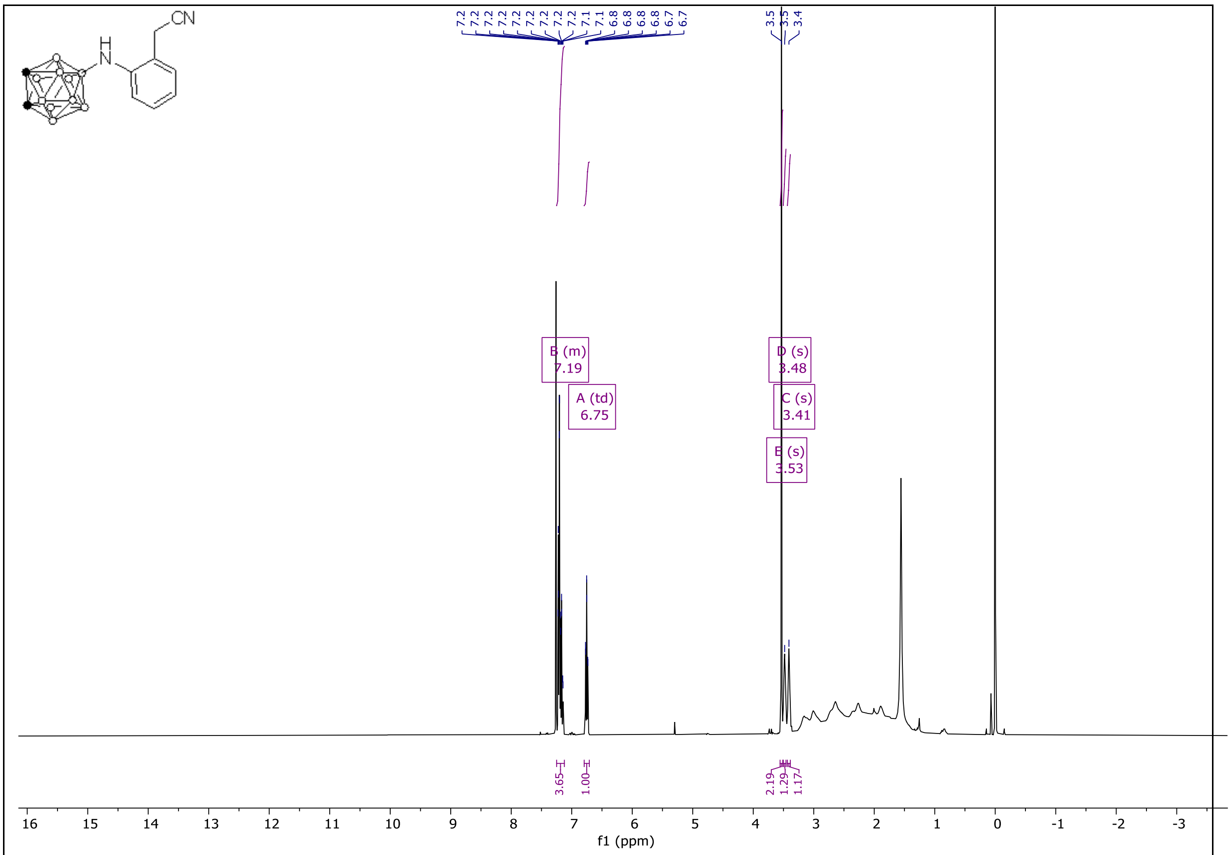

*Figure S 41. ¹H NMR spectrum of **9** in DMSO-d_6.*

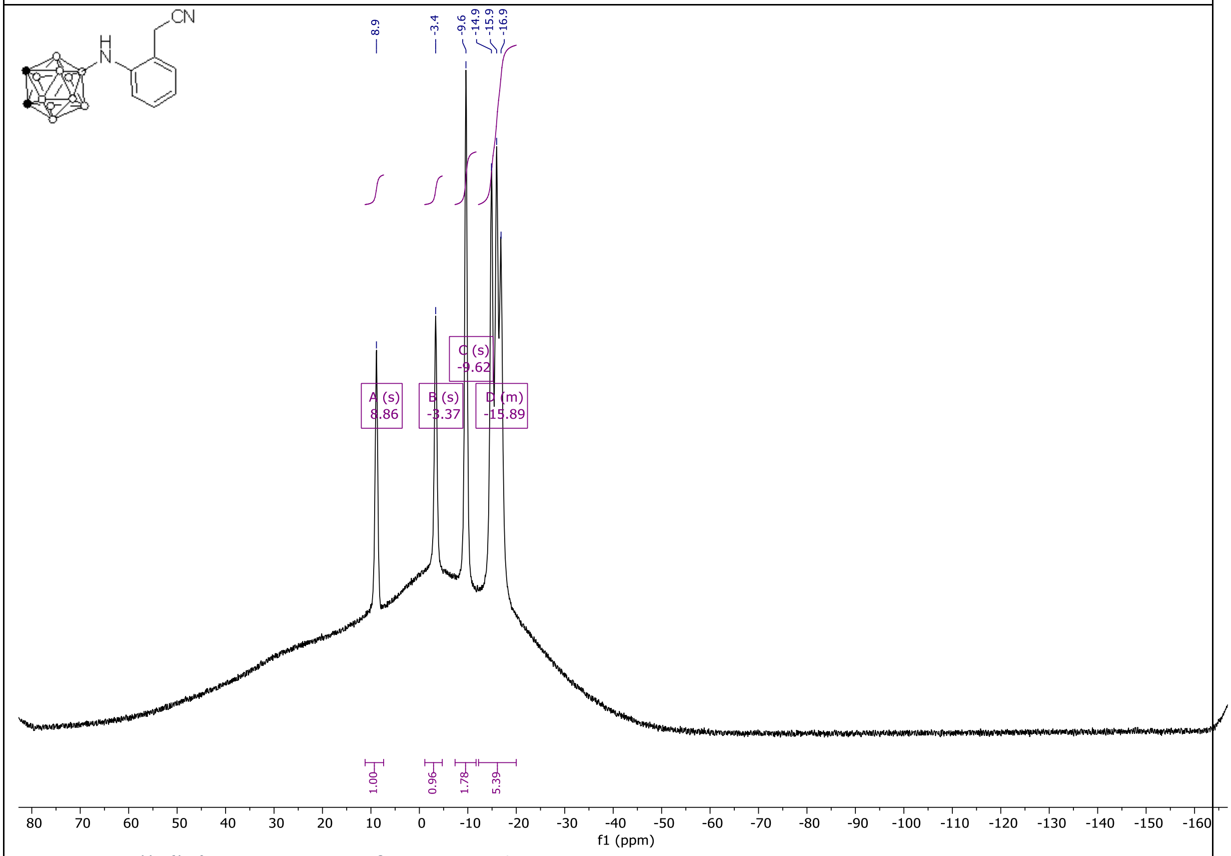

*Figure S 42. ¹¹B{¹H} NMR spectrum of **9** in DMSO-d_6.*

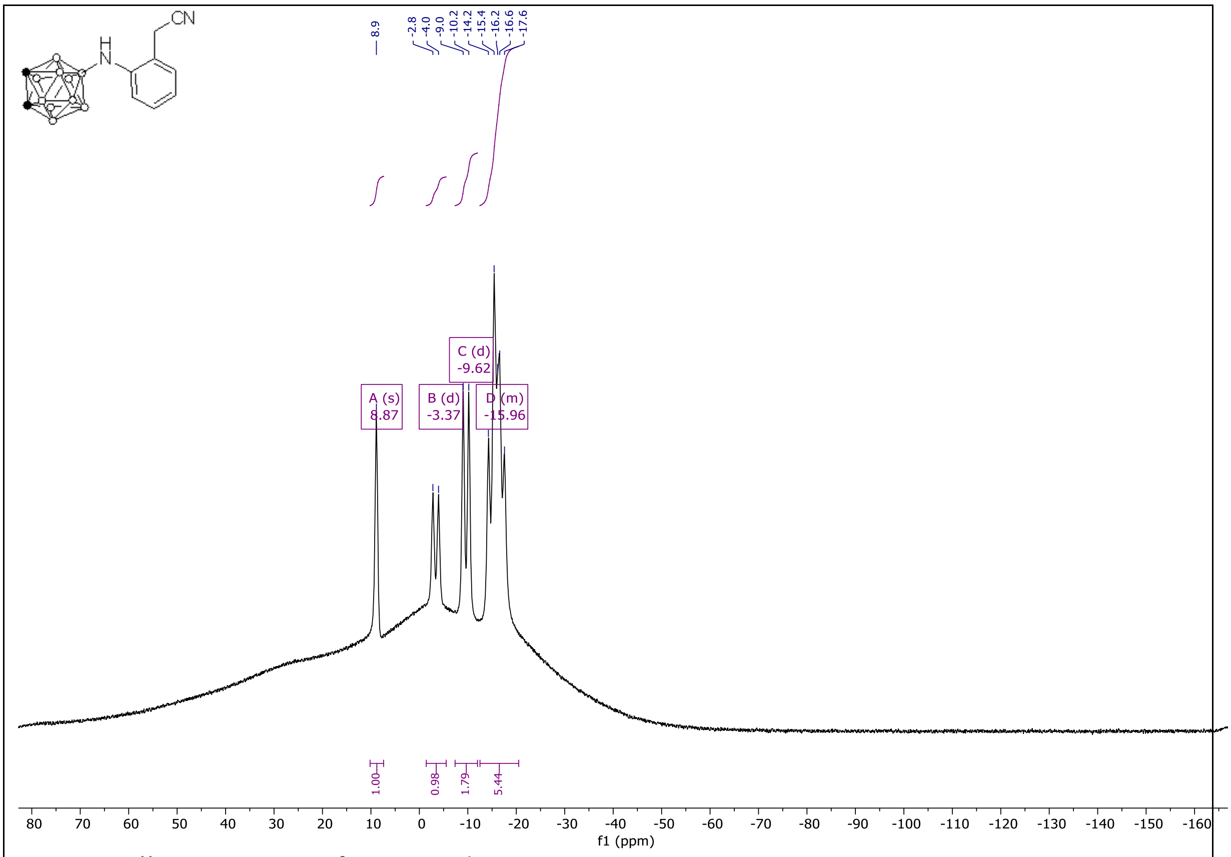

Figure S 43. ^{11}B NMR spectrum of **9** in DMSO-d_6.

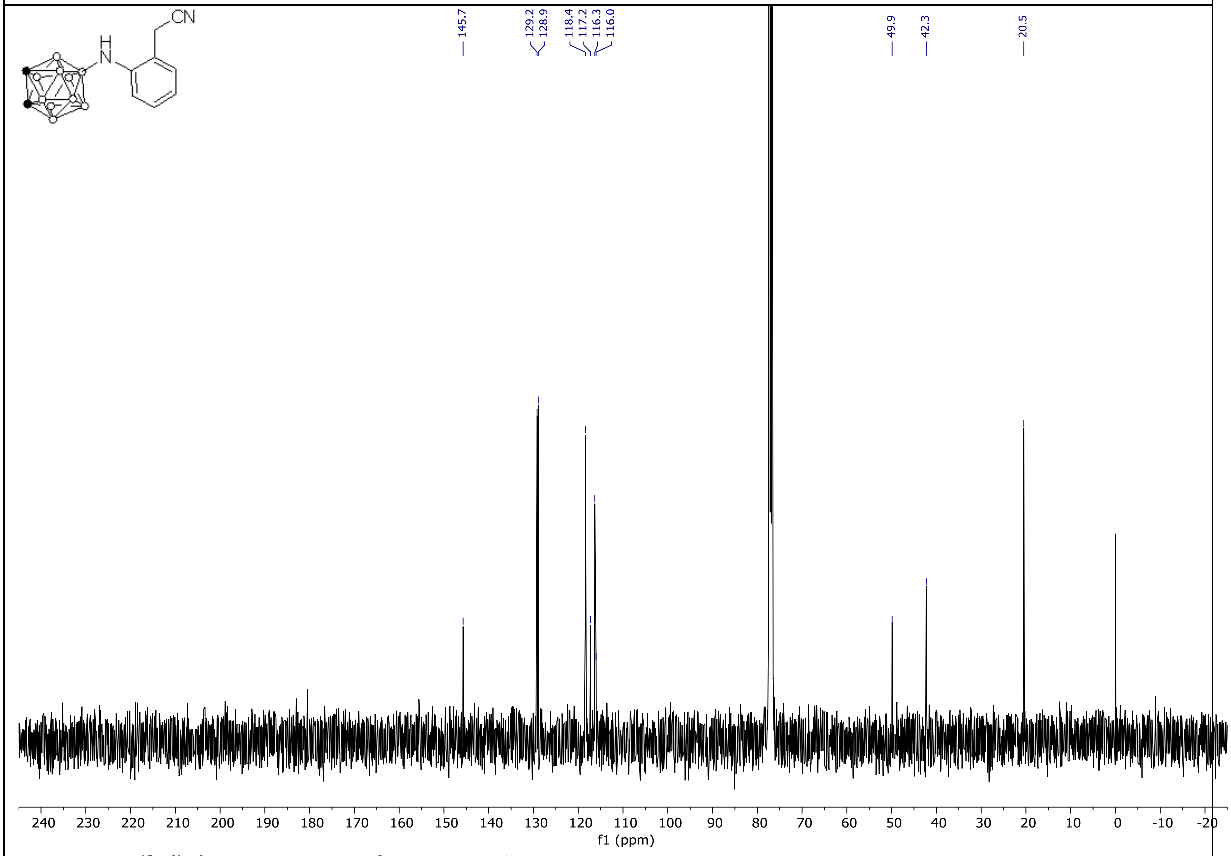

Figure S 44. $^{13}C\{^1H\}$ NMR spectrum of **9** in DMSO-d_6.

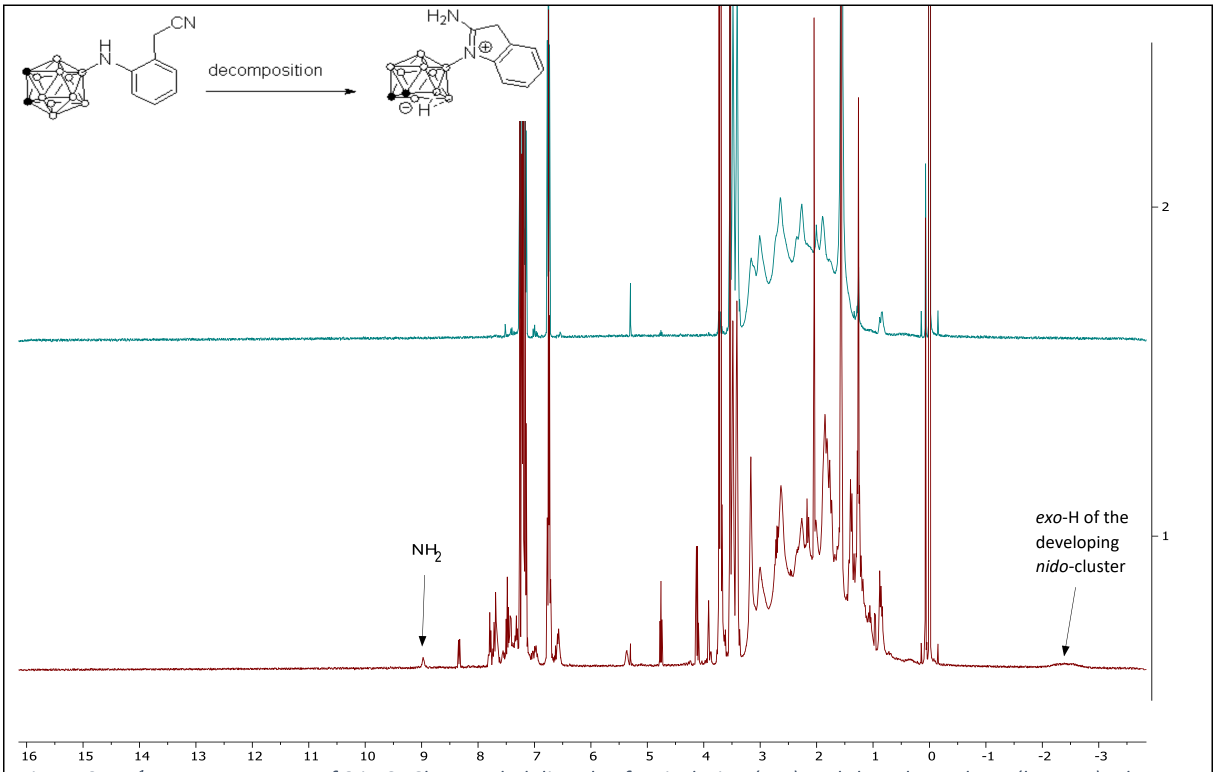

*Figure S 45. ^{1}H NMR spectrum of **9** in $CDCl_3$ recorded directly after isolation (top) and three hours later (bottom). The emerging signals of the nido cluster's exo-H and the amine group of the cyclisation product are indicted with arrows.*

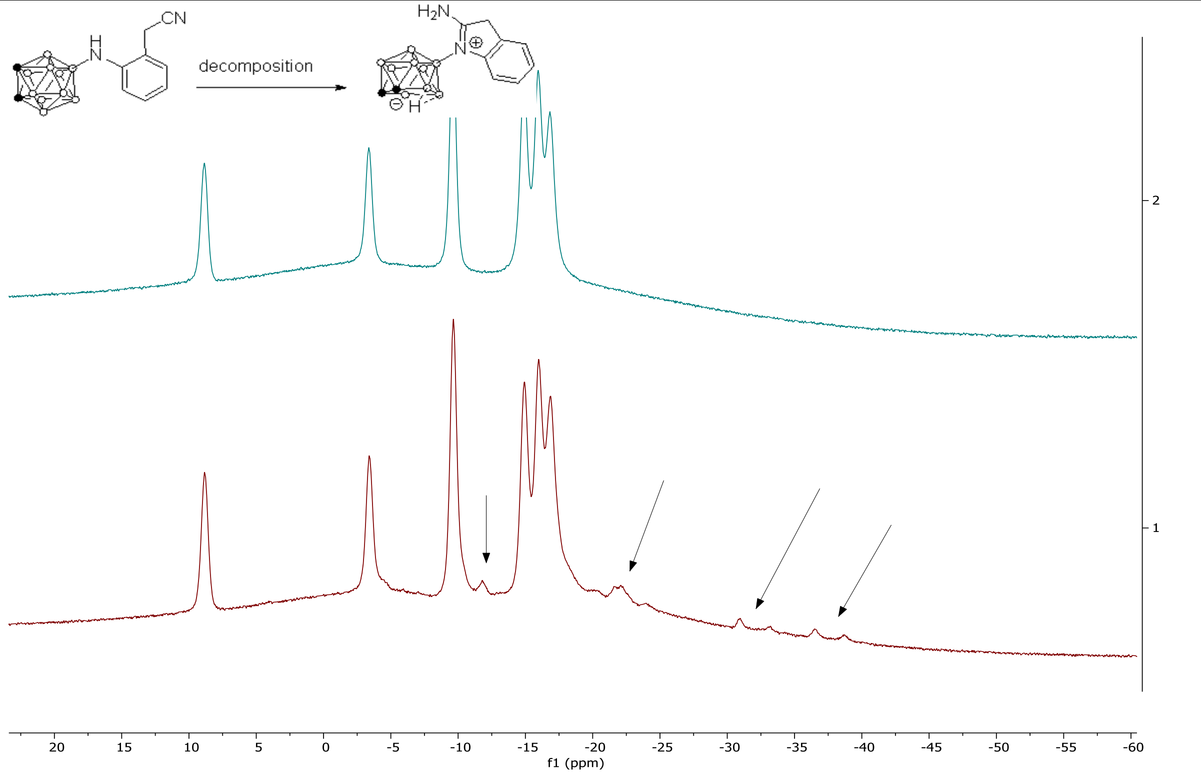

*Figure S 46. ^{11}B{^{1}H} NMR spectrum of **9** in $CDCl_3$ recorded directly after isolation (top) and three hours later (bottom). The emerging signals of the nido cluster's exo-H and the amine group of the cyclisation product are indicted with arrows.*

8 HR-ESI MS Data for Compounds *o*1, *m*1, *p*1, 2, 3 and 9

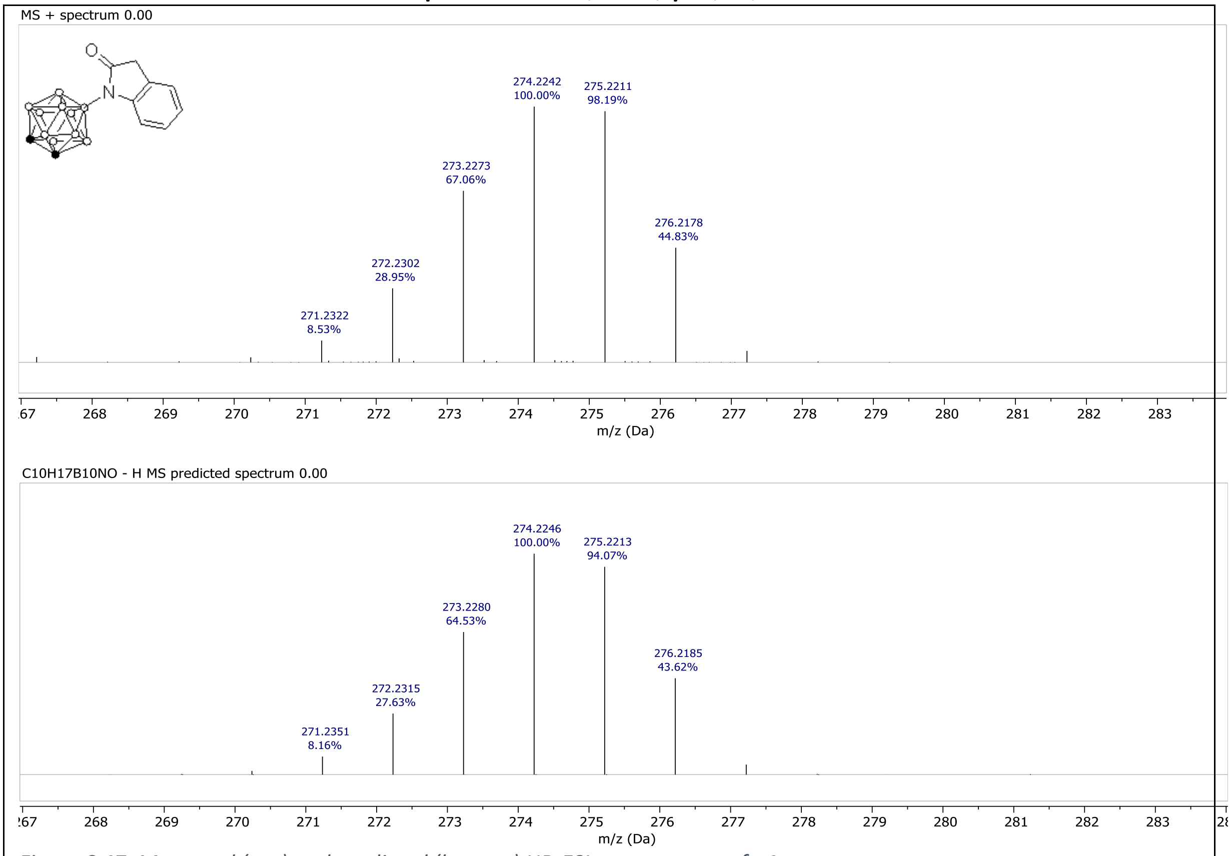

Figure S 47. Measured (top) and predicted (bottom) HR-ESI mass spectra of ***o1****.*

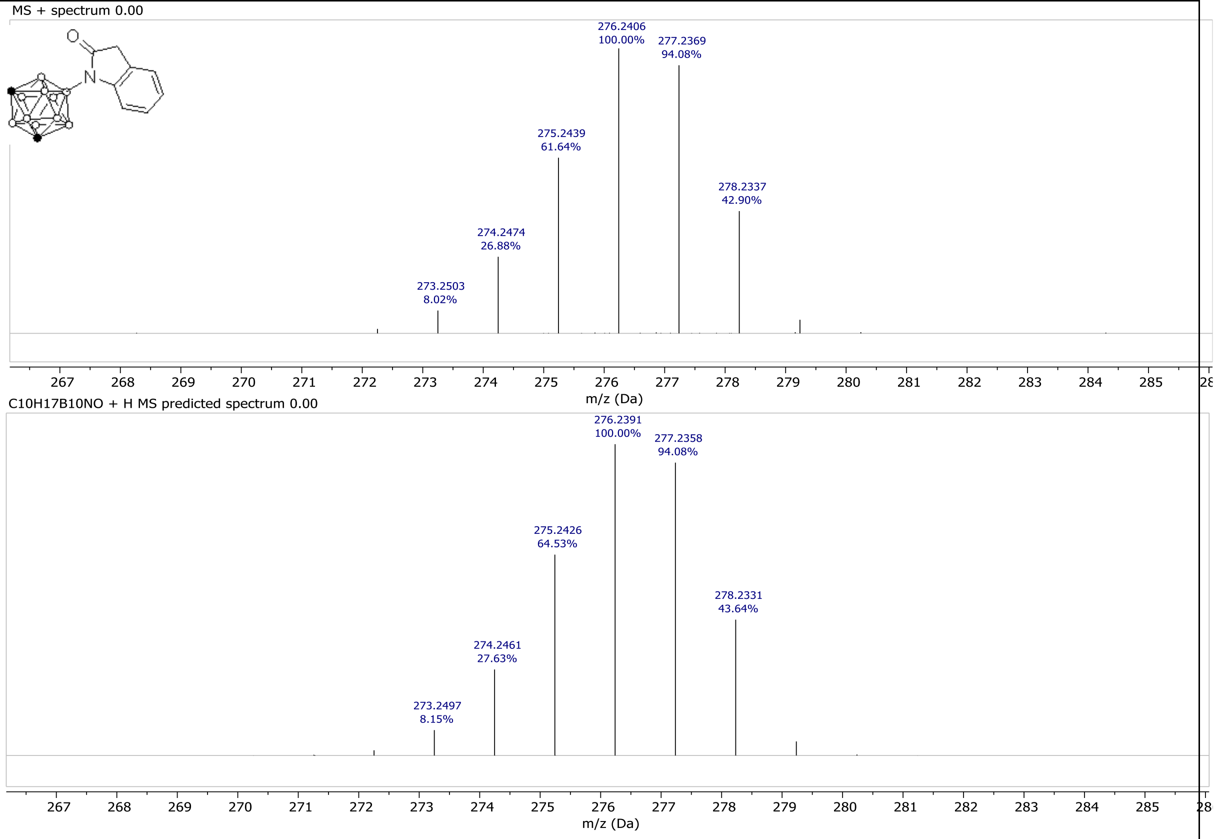

Figure S 48. Measured (top) and predicted (bottom) HR-ESI mass spectra of ***m1****.*

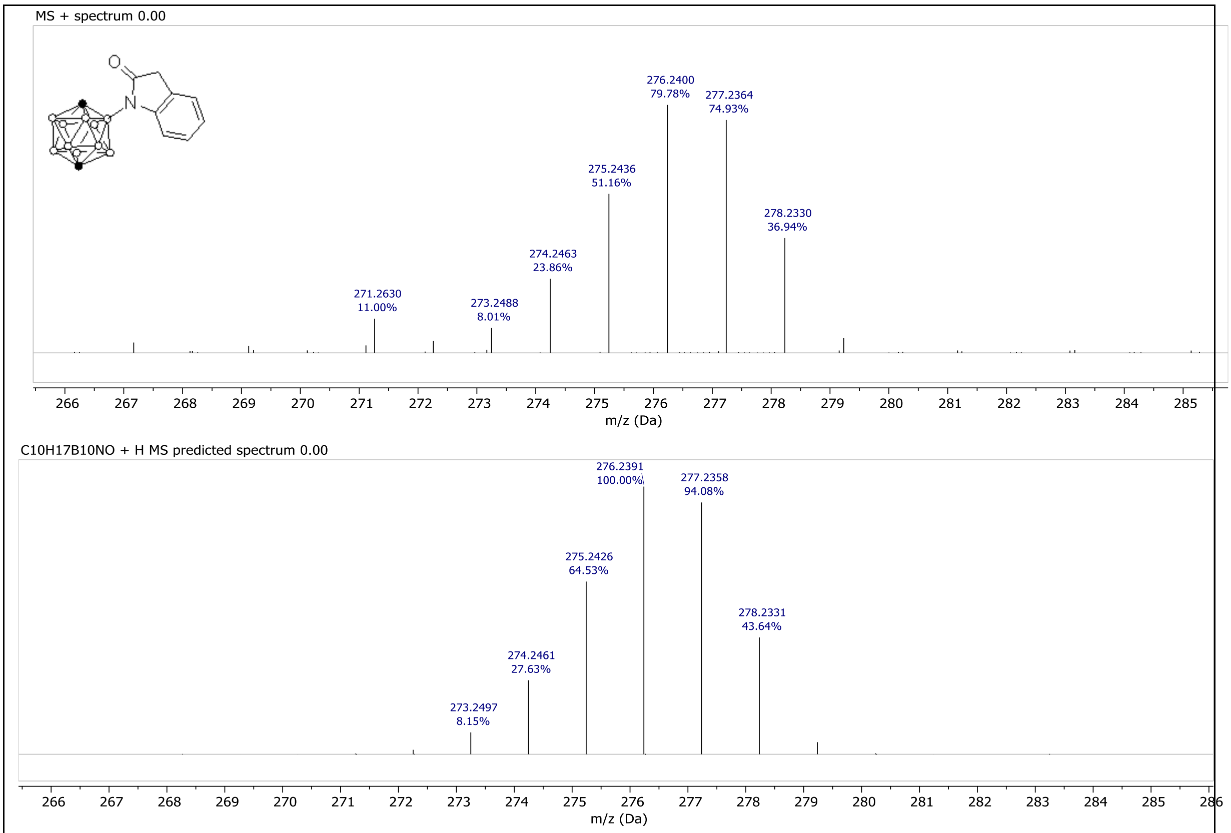

Figure S 49. Measured (top) and predicted (bottom) HR-ESI mass spectra of ***p1****.*

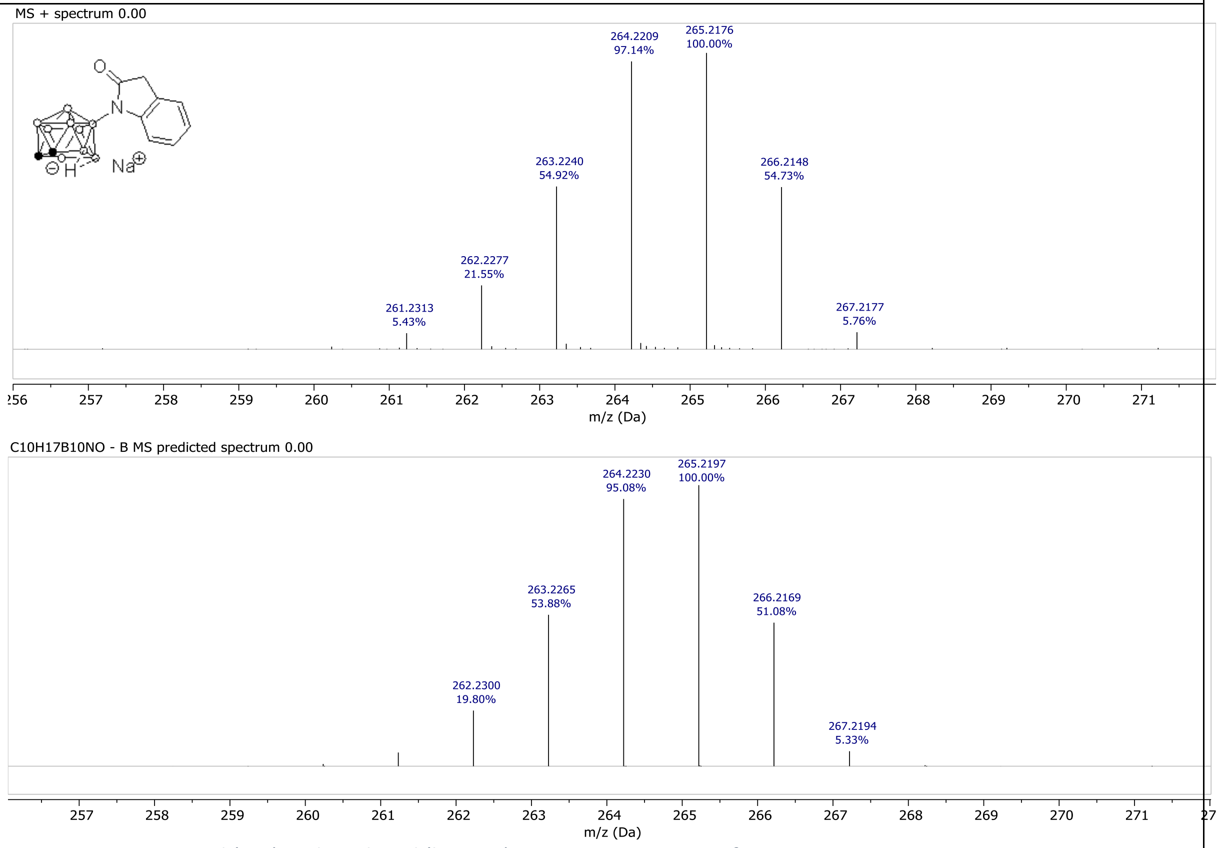

Figure S 50. Measured (top) and predicted (bottom) HR-ESI mass spectra of ***2****.*

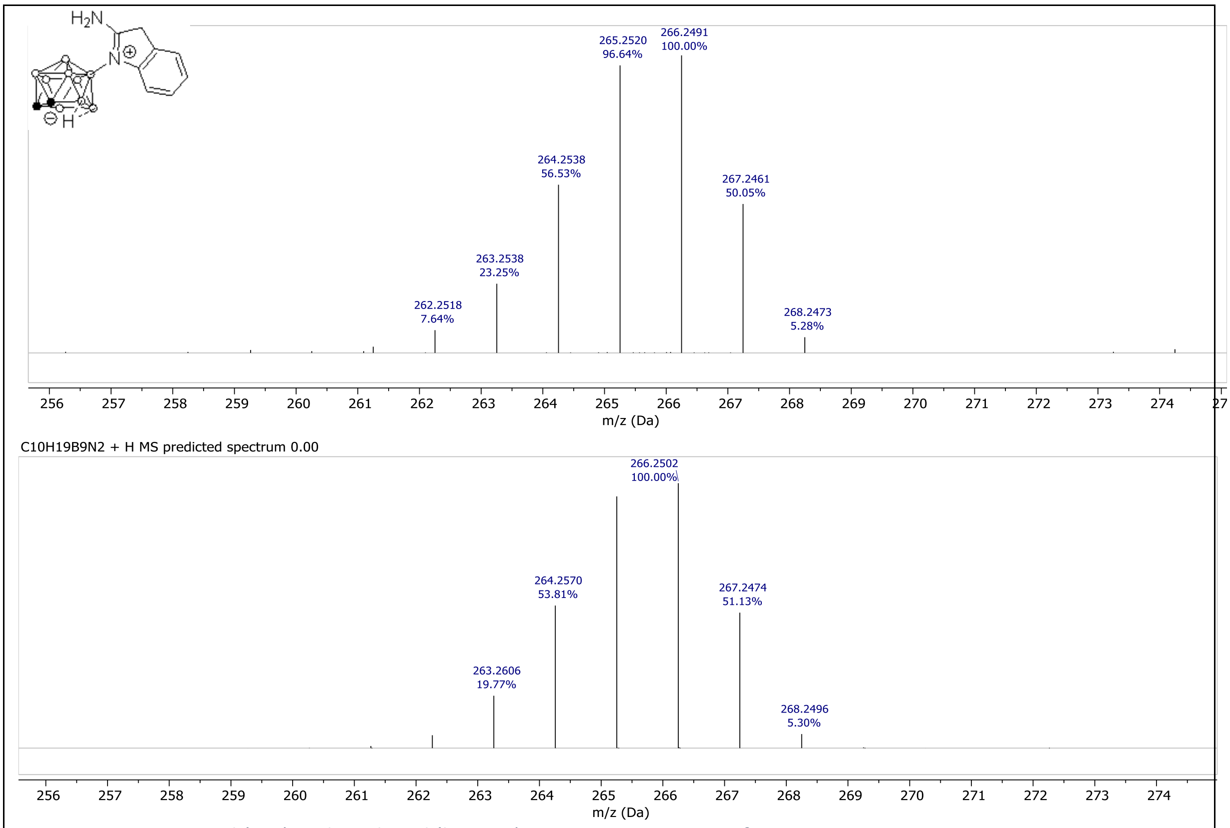

*Figure S 51. Measured (top) and predicted (bottom) HR-ESI mass spectra of **3**.*

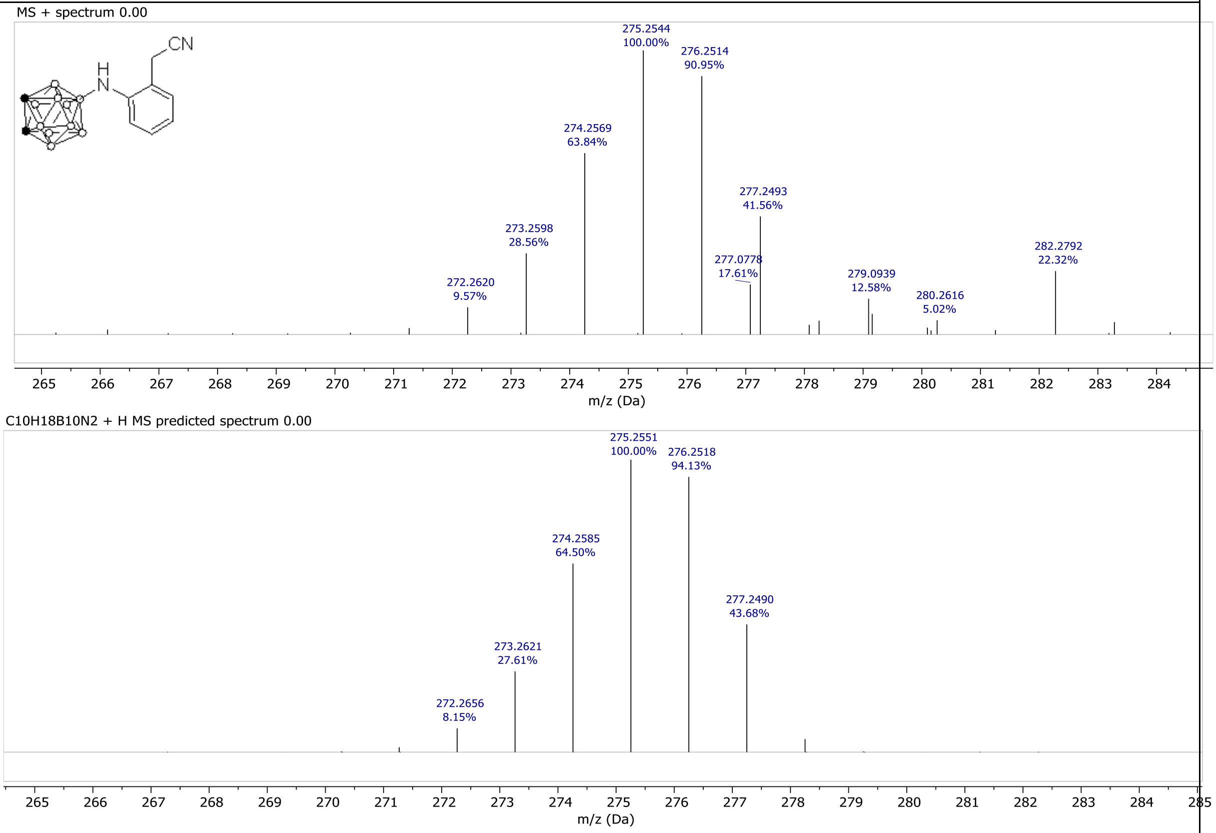

*Figure S 52. Measured (top) and predicted (bottom) HR-ESI mass spectra of **9**.*

9 HPLC Data for compounds *o*1, *m*1, *p*1, 2, 3 and CCF

Chromatogram

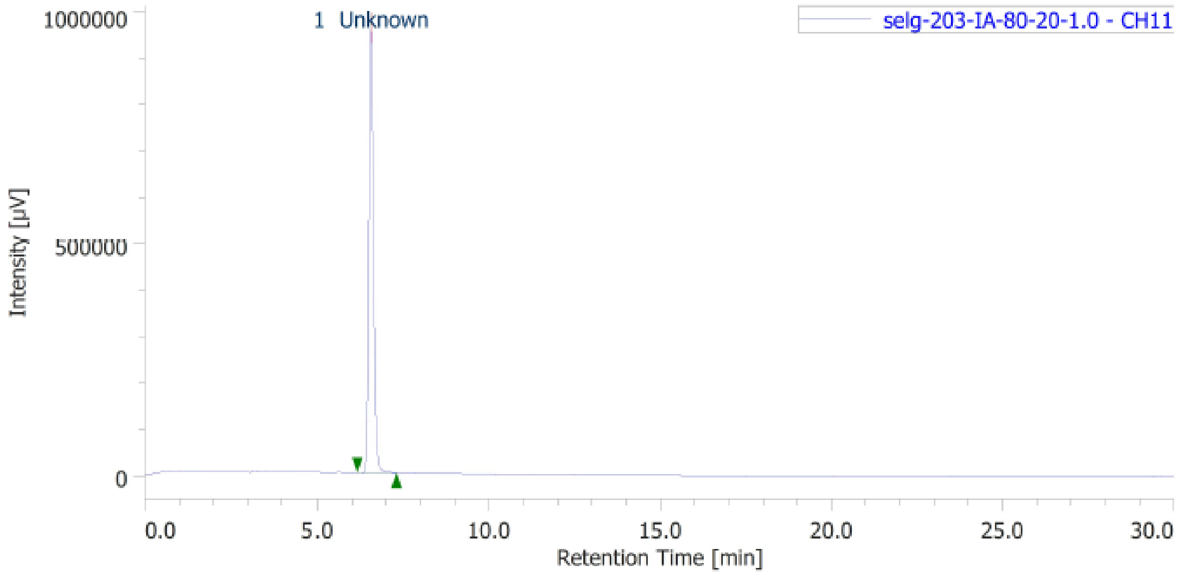

Chromatogram Information	
User Name	User
Date Modified	19.07.2023 07:35:18
Description	
HPLC System Name	HPLC (Melania)
Injection Date	18.07.2023 22:00:22
Volume	20.0 [µL]
Sample #	7
Project Name	Jun2023
Acquisition Time	30.0 [min]
Acquisition Sequence	18.07.2023 Tag2
Control Method	IA-30min-80A-20B-0C-0D-1.0
Peak ID Table	
Calibration Method	
Additional Information	

Channel & Peak Information Table	
Chromatogram Name	selg-203-IA-80-20-1.0-CH11
Sample Name	
Channel Name	246,0nm
Sampling Interval	200 [msec]
Peak Method	(Manual)

#	Peak Name	CH	tR [min]	Area [µV·sec]	Height [µV]	Area%	Height%	Quantity	NTP	Resolution	Symmetry Factor	Warning
1	Unknown	11	6.543	8876686	958322	100.000	100.000	N/A	12456	N/A	1.227	

Figure S 53. HPLC chromatogram of ***CCF****.*

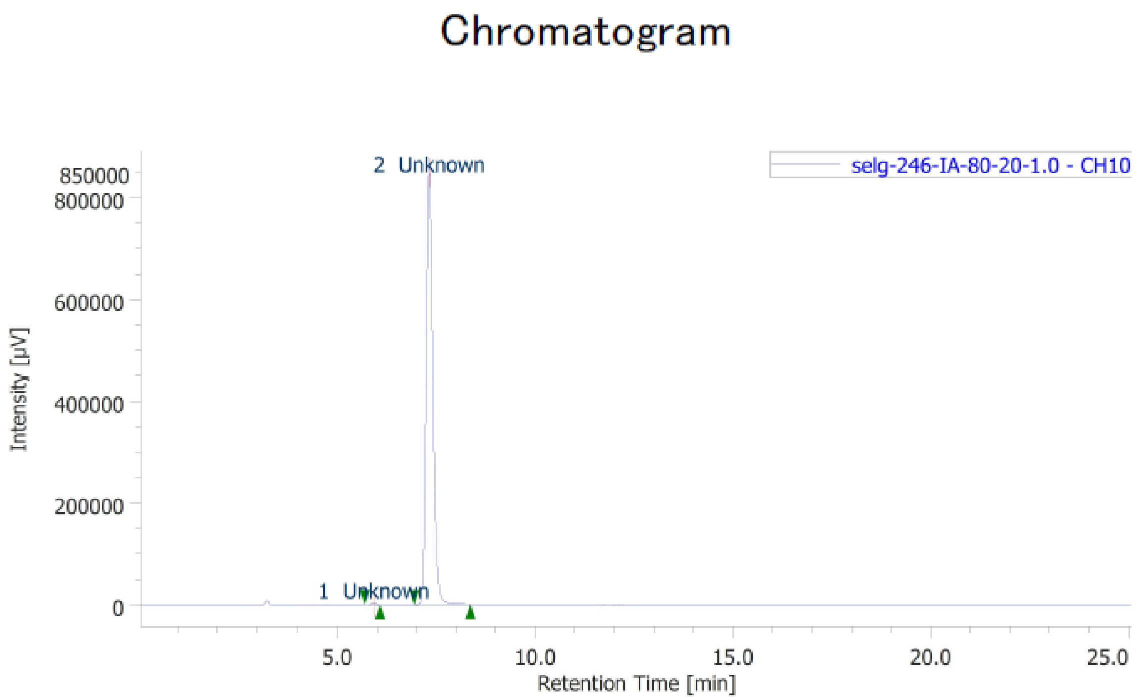

Chromatogram Information

User Name	User
Date Modified	19.07.2023 07:50:51
Description	
HPLC System Name	HPLC (Melania)
Injection Date	19.07.2023 02:32:44
Volume	20.0 [µL]
Sample #	14
Project Name	Jun2023
Acquisition Time	30.0 [min]
Acquisition Sequence	18.07.2023 Tag2
Control Method	IA-30min-80A-20B-0C-0D-1.0
Peak ID Table	
Calibration Method	
Additional Information	

Channel & Peak Information Table

Chromatogram Name	selg-246-IA-80-20-1.0-CH10
Sample Name	
Channel Name	230.0nm
Sampling Interval	200 [msec]
Peak Method	(Manual)

#	Peak Name	CH	tR [min]	Area [µV·sec]	Height [µV]	Area%	Height%	Quantity	NTP	Resolution	Symmetry Factor	Warning
1	Unknown	10	5.907	19596	2175	0.197	0.256	N/A	9871	5.275	0.964	
2	Unknown	10	7.313	9910530	848135	99.803	99.744	N/A	9705	N/A	1.239	

Figure S 54. HPLC chromatogram of ***o1****.*

Chromatogram

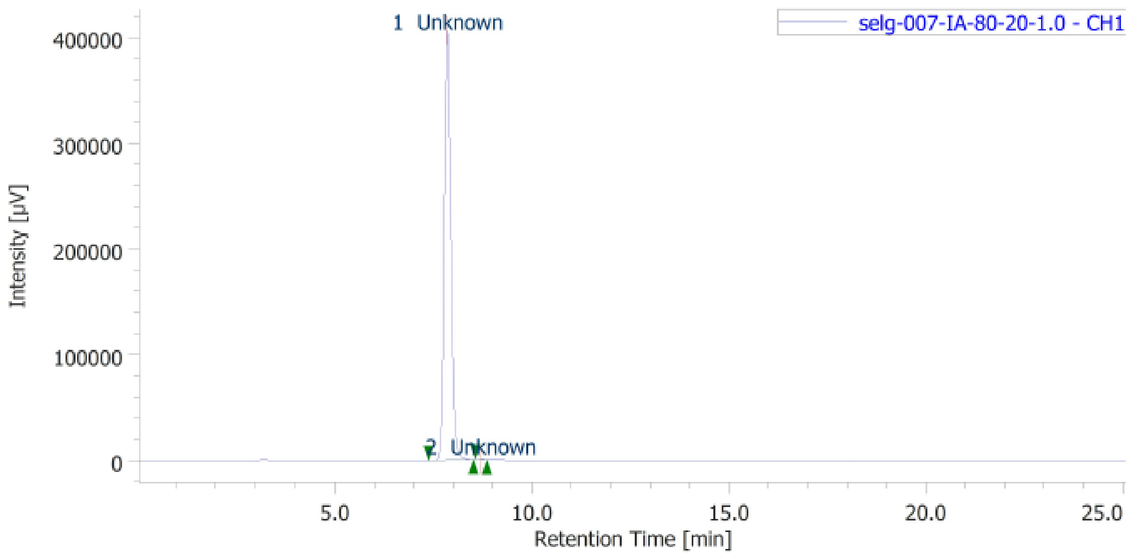

Chromatogram Information

User Name	User
Date Modified	19.07.2023 07:52:24
Description	
HPLC System Name	HPLC (Melania)
Injection Date	19.07.2023 03:18:08
Volume	20.0 [µL]
Sample #	15
Project Name	Jun2023
Acquisition Time	30.0 [min]
Acquisition Sequence	18.07.2023 Tag2
Control Method	IA-30min-80A-20B-0C-0D-1.0
Peak ID Table	
Calibration Method	
Additional Information	

Channel & Peak Information Table

Chromatogram Name	selg-007-IA-80-20-1.0-CH1
Sample Name	
Channel Name	CH1
Sampling Interval	500 [msec]
Peak Method	(Manual)

#	Peak Name	CH	tR [min]	Area [µV·sec]	Height [µV]	Area%	Height%	Quantity	NTP	Resolution	Symmetry Factor	Warning
1	Unknown	1	7.850	4669992	405838	99.939	99.928	N/A	11450	2.979	1.139	
2	Unknown	1	8.700	2843	294	0.061	0.072	N/A	15588	N/A	1.049	

*Figure S 55. HPLC chromatogram of **m1**.*

Chromatogram

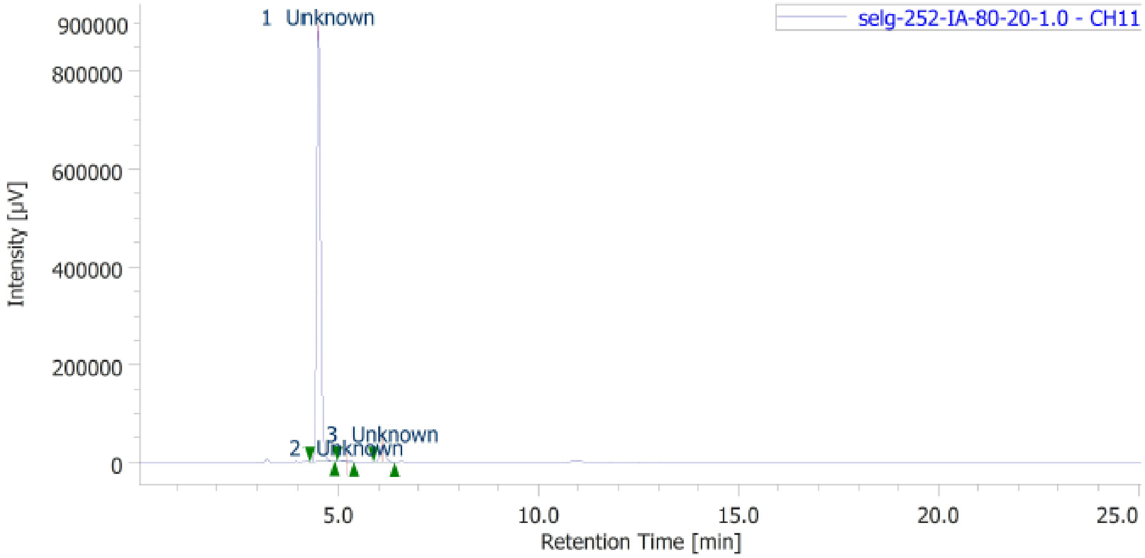

Chromatogram Information	
User Name	User
Date Modified	19.07.2023 07:54:02
Description	
HPLC System Name	HPLC (Melania)
Injection Date	19.07.2023 04:03:32
Volume	20.0 [µL]
Sample #	16
Project Name	Jun2023
Acquisition Time	30.0 [min]
Acquisition Sequence	18.07.2023 Tag2
Control Method	IA-30min-80A-20B-0C-0D-1.0
Peak ID Table	
Calibration Method	
Additional Information	

Channel & Peak Information Table	
Chromatogram Name	selg-252-IA-80-20-1.0-CH11
Sample Name	
Channel Name	233,0nm
Sampling Interval	200 [msec]
Peak Method	(Manual)

#	Peak Name	CH	tR [min]	Area [µV·sec]	Height [µV]	Area%	Height%	Quantity	NTP	Resolution	Symmetry Factor	Warning
1	Unknown	11	4.497	5922918	891755	95.045	96.413	N/A	11442	2.839	1.170	
2	Unknown	11	5.213	25695	2234	0.412	0.242	N/A	3806	3.059	0.948	
3	Unknown	11	6.090	283092	30942	4.543	3.345	N/A	10586	N/A	1.190	

Figure S 56. HPLC chromatogram of ***p1****.*

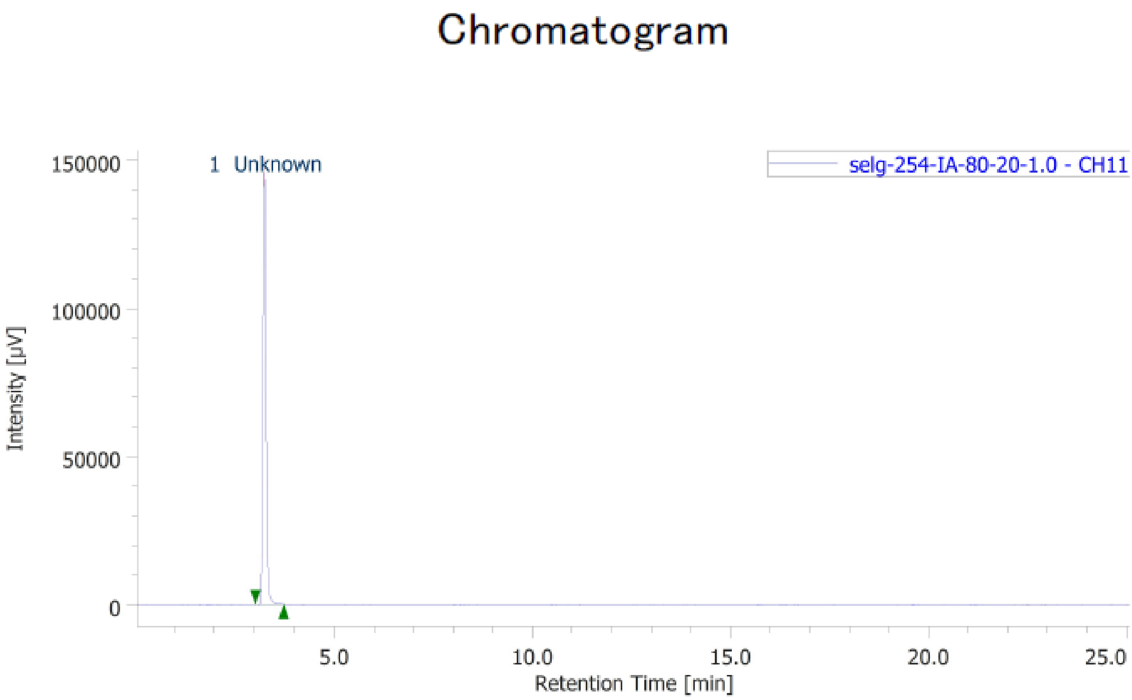

Chromatogram Information	
User Name	User
Date Modified	19.07.2023 08:00:15
Description	
HPLC System Name	HPLC (Melania)
Injection Date	19.07.2023 06:19:42
Volume	20.0 [µL]
Sample #	2
Project Name	Jun2023
Acquisition Time	30.0 [min]
Acquisition Sequence	18.07.2023 Tag2
Control Method	IA-30min-80A-20B-0C-0D-1.0
Peak ID Table	
Calibration Method	
Additional Information	

Channel & Peak Information Table	
Chromatogram Name	selg-254-IA-80-20-1.0-CH11
Sample Name	
Channel Name	209.0nm
Sampling Interval	200 [msec]
Peak Method	(Manual)

#	Peak Name	CH	tR [min]	Area [µV·sec]	Height [µV]	Area%	Height%	Quantity	NTP	Resolution	Symmetry Factor	Warning
1	Unknown	11	3.270	810737	145669	100.000	100.000	N/A	8458	N/A	1.144	

Figure S 57. HPLC chromatogram of ***2****.*

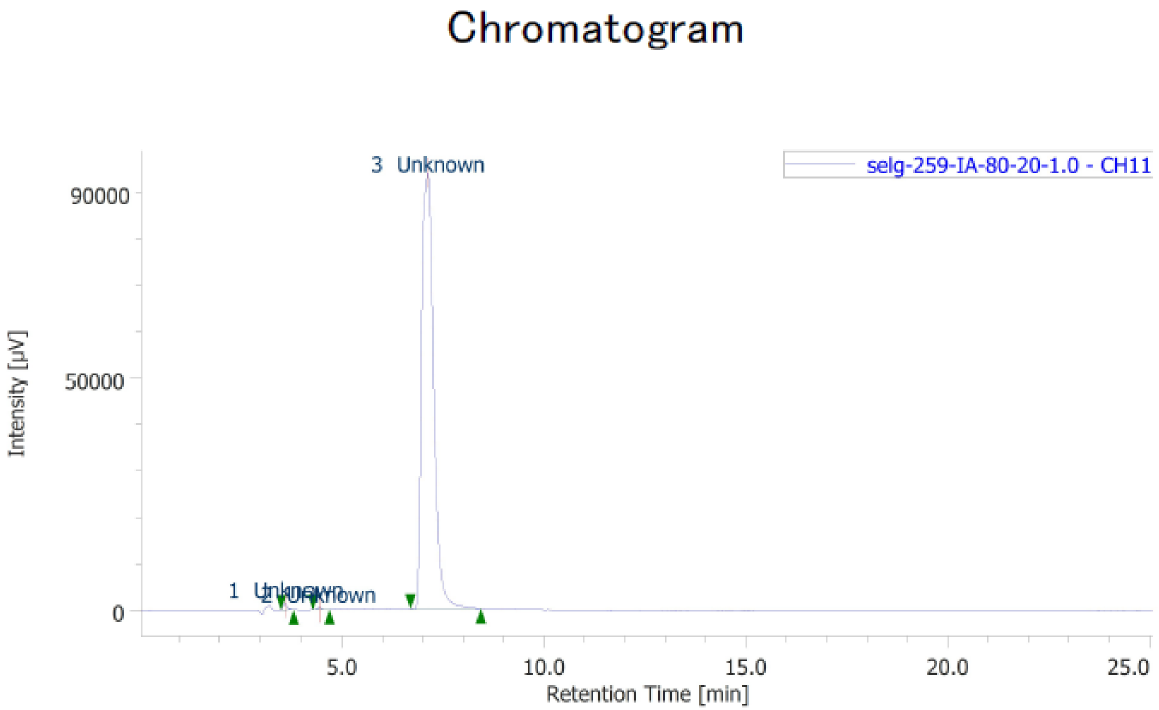

Chromatogram Information	
User Name	User
Date Modified	19.07.2023 08:02:01
Description	
HPLC System Name	HPLC (Melania)
Injection Date	19.07.2023 07:20:18
Volume	20.0 [µL]
Sample #	19
Project Name	Jun2023
Acquisition Time	30.0 [min]
Acquisition Sequence	18.07.2023 Tag2
Control Method	IA-30min-80A-20B-0C-0D-1.0
Peak ID Table	
Calibration Method	
Additional Information	

Channel & Peak Information Table	
Chromatogram Name	selg-259-IA-80-20-1.0-CH11
Sample Name	
Channel Name	253.0nm
Sampling Interval	200 [msec]
Peak Method	(Manual)

#	Peak Name	CH	tR [min]	Area [µV·sec]	Height [µV]	Area%	Height%	Quantity	NTP	Resolution	Symmetry Factor	Warning
1	Unknown	11	3.623	9392	1484	0.481	1.548	N/A	8004	4.872	1.391	
2	Unknown	11	4.430	3183	489	0.163	0.510	N/A	10858	7.542	1.131	
3	Unknown	11	7.120	1941550	93896	99.356	97.943	N/A	2729	N/A	1.269	

*Figure S 58. HPLC chromatogram of **3**.*
